# Effectiveness of school‐based programs to reduce bullying perpetration and victimization: An updated systematic review and meta‐analysis

**DOI:** 10.1002/cl2.1143

**Published:** 2021-04-05

**Authors:** Hannah Gaffney, Maria M. Ttofi, David P. Farrington

**Affiliations:** ^1^ Institute of Criminology University of Cambridge Cambridge UK

## Abstract

**Background:**

Bullying first emerged as an important topic of research in the 1980s in Norway (Olweus), and a recent meta‐analysis shows that these forms of aggression remain prevalent among young people globally (Modecki et al.). Prominent researchers in the field have defined bullying as any aggressive behavior that incorporates three key elements, namely: (1) an intention to harm, (2) repetitive in nature, and (3) a clear power imbalance between perpetrator and victim (Centers for Disease Control and Prevention; Farrington). There are many negative outcomes associated with bullying perpetration, such as: suicidal ideation (Holt et al.), weapon carrying (Valdebenito et al.), drug use (Ttofi et al.), and violence and offending in later life (Ttofi et al.). Bullying victimization too is associated with negative outcomes such as: suicidal ideation (Holt et al.), anxiety, low self‐esteem and loneliness (Hawker& Boulton). Therefore, school bullying is an important target for effective intervention, and should be considered a matter of public health concern.

**Objectives:**

The objective of this review is to establish whether or not existing school‐based antibullying programs are effective in reducing school‐bullyng behaviors. This report also updates a previous meta‐analysis conducted by Farrington and Ttofi. This earlier review found that antibullying programs are effective in reducing bullying perpetration and victimization and a primary objective of the current report is to update the earlier analysis of 53 evaluations by conducting new searches for evaluations conducted and published since 2009.

**Search Methods:**

Systematic searches were conducted using Boolean combinations of the following keywords: bully*; victim*; bully‐victim; school; intervention; prevention; program*; evaluation; effect*; and *anti‐bullying*. Searches were conducted on several online databases including, Web of Science, PscyhINFO, EMBASE, EMBASE, DARE, ERIC, Google Scholar, and Scopus. Databases of unpublished reports, such as masters' and doctoral theses (e.g., Proquest) were also searched.

**Selection Criteria:**

Results from systematic searches were screened thoroughly against the following inclusion criteria. To be included in this review, a study must have: (1) described an evaluation of a school‐based antibullying program implemented with school‐age participants; (2) utilized an operational definition of school‐bullying that coincides with existing definitions; (3) measured school‐bullying perpetration and/or victimization using quantitative measures, such as, self‐, peer‐, or teacher‐report questionnaires; and (4) used an experimental or quasi‐experimental design, with one group receiving the intervention and another not receiving the intervention.

**Data Collection and Analysis:**

Of the 19,877 search results, 474 were retained for further screening. The majority of these were excluded, and after multiple waves of screening, 100 evaluations were included in our meta‐analysis. A total of 103 independent effect sizes were estimated and each effect size was corrected for the impact of including clusters in evaluation designs. Included evaluations were conducted using both randomized (*n* = 45; i.e., randomized controlled trials/RCTs) and nonrandomized (*n* = 44; i.e., quasi‐experimental designs with before/after measures; BA/EC) methodologies. All of these studies included measures of bullying outcomes before and after implementation of an intervention. The remaining 14 effect sizes were estimated from evaluations that used age cohort designs. Two models of meta‐analysis are used to report results in our report. All mean effects computed are presented using both the multivariance adjustment model (MVA) and random effects model (RE). The MVA model assigns weights to primary studies in direct proportion to study level sampling error as with the fixed effects model but adjusts the meta‐analytic standard error and confidence intervals for study heterogeneity. The RE model incorporates between‐study heterogeneity into the formula for assigning weights to primary studies. The differences and strengths/limitations of both approaches are discussed in the context of the present data.

**Results:**

Our meta‐analysis identified that bullying programs significantly reduce bullying perpetration (RE: odds ratio [OR] = 1.309; 95% confidence interval [CI]: 1.24–1.38; *z* = 9.88; *p* < .001) and bullying victimization (RE: OR = 1.244; 95% CI: 1.19–1.31; *z* = 8.92; *p* < .001), under a random effects model of meta‐analysis. Mean effects were similar across both models of meta‐analysis for bullying perpetration (i.e., MVA: OR = 1,324; 95% CI: 1.27–1.38; *z* = 13.4; *p* < .001) and bullying victimization (i.e., MVA: OR = 1.248; 95% CI: 1.21–1.29; *z* = 12.06; *p* < .001). Under both computational models, primary studies were more effective in reducing bullying perpetration than victimization overall. Effect sizes varied across studies, with significant heterogeneity between studies for both bullying perpetration (*Q* = 323.392; *df* = 85; *p* < .001; *I* ^*2*^ = 73.716) and bullying victimization (*Q* = 387.255; *df* = 87; *p* < .001; *I* ^*2*^ = 77.534) outcomes. Analyses suggest that publication bias is unlikely. Between‐study heterogeneity was expected, given the large number of studies included, and thus, the number of different programs, methods, measures and samples used.

**Authors' Conclusions:**

We conclude that overall, school‐based antibullying programs are effective in reducing bullying perpetration and bullying victimization, although effect sizes are modest. The impact of evaluation methodology on effect size appears to be weak and does not adequately explain the significant heterogeneity between primary studies. Moreover, the issue of the under‐/over‐estimation of the true treatment effect by different experimental designs and use of self‐reported measures is reviewed. The potential explanations for this are discussed, along with recommendations for future primary evaluations. Avenues for future research are discussed, including the need further explain differences across programs by correlating individual effect sizes with varying program components and varying methodological elements available across these 100 evaluations. Initial findings in the variability of effect sizes across different methodological moderators provide some understanding on the issue of heterogeneity, but future analyses based on further moderator variables are needed.

## 1 PLAIN LANGUAGE SUMMARY

### 1.1 Interventions to reduce school bullying perpetration and victimization are effective

Bullying is a ubiquitous form of aggression in schools worldwide. Intervention and prevention programs targeting school bullying perpetration and victimization are effective, yet more research is needed to understand variability in effectiveness.

The main findings of our review are that bullying programs were effective in reducing bullying perpetration outcomes by roughly 18–19% and bullying victimization by roughly 15–16%. There are substantial variations in effects, and the reasons for these variations require further research.

### 1.2 What is this review about?

Bullying is defined as aggressive behaviors that occur repeatedly over time between two or more individuals. Typically, there is a clear power imbalance between victims and bullies, either socially or physically. Furthermore, bullying behaviors are those that are committed intentionally to harm the victim.

**What is the aim of this review?**

The aim of this review is to summarise findings from studies of the effectiveness of school‐based antibullying programs in reducing both bullying perpetration and victimization will be reported. The review summarizes 100 studies, with the largest number being from the United States.

### 1.3 What studies are included?

To be included in this review, primary studies must have evaluated a specific intervention program that targeted bullying perpetration and/or victimization outcomes in school‐aged children, that is, typically between four and 18 years old. Studies must have used two experimental groups of children, one that received the intervention, and one that did not, and applied quantitative measures of bullying behavior (perpetration and/or victimization) that coincided with our operational definition of bullying.

Our final meta‐analytic review includes 100 studies of the effectiveness of antibullying programs. The largest number of studies came from the United States, with most other studies from Canada and Europe.

### 1.4 What are the findings of this review?

Antibullying programs are effective in reducing bullying perpetration outcomes by roughly 18–19% and bullying victimization by roughly 15–16%.

Variability in the effectiveness of antibullying programs was associated with differences in methodological designs, types of programs and geographical regions. Interventions evaluated using age cohort designs collectively gave the largest overall effect for both bullying perpetration and bullying victimization.

Limitations of the results are similar to those of previous reviews; for example, the reliance of self‐reported measurements of bullying may suggest the change is in reports of bullying perpetration/victimization and not behavioral change.

### 1.5 What do the findings of this review mean?

The findings indicate that school‐based bullying intervention and prevention programs can be effective in reducing both bullying perpetration and victimization, although the effect is, overall, modest.

The effectiveness of antibullying programs is an important finding with implications for public health and educational policy. However, our review did identify that there are variations in the effectiveness of intervention programs. Future research is needed to explore the reasons for these variations.

### 1.6 How up‐to‐date is this review?

This report forms an update of an earlier review (Farrington & Ttofi, 2009). The review authors searched for studies published up to December 2016.

## 2 BACKGROUND

Bullying first emerged as an important topic of research in the 1980s, following the tragic suicides of young boys in Norway, the reason for which was attributed to bullying victimization (Olweus, 1993). Today, this form of aggressive behavior remains a prevalent problem among young people globally. For example, a recent meta‐analysis of 80 international studies discovered prevalence levels of 34.5% and 36% for bullying perpetration and bullying victimization respectively (Modecki et al., 2014).

Notably, bullying is a matter of public health, impacting the life outcomes of both bullies and victims, in varying ways (Arseneault et al., 2010; Masiello & Schroeder, 2014; Ttofi et al., 2012). Given its long‐term effects, it is imperative that effective intervention efforts are put in place in order to alleviate this troubling school phenomenon (Ttofi, 2015).

### 2.1 Defining school bullying

In order to adequately determine which interventions will effectively reduce bullying behaviors, it is important that researchers and educators start by accurately assessing the prevalence of involvement in school bullying (Swearer et al., 2010). There remains some degree of disagreement in relation to definitive cut‐off points for involvement in bullying (Solberg & Olweus, 2003; Swearer et al., 2010) and methods utilized for the assessment of bullying (Smith et al., 2002; Swearer et al., 2010). However, there is better agreement in regard to the defining criteria for school bullying.

Prominent researchers in the field have defined bullying as any aggressive behavior that incorporates three core elements, namely: (1) an intention to harm, (2) repetitive in nature, and (3) a clear power imbalance between perpetration and victim (Centers for Disease Control and Prevention, 2014; Farrington, 1993; Olweus, 1993). In other words, bullies are individuals who intend to cause harm to their victims through their actions, over a long period of time. Furthermore, victims of bullying are typically less powerful than bullies, or groups of bullies, and feel that they cannot easily defend themselves. This may be due to a physical or social power imbalance.

There are many forms of bullying, for example, school‐bullying, workplace bullying, sibling bullying and, most recently, cyberbullying. The present review is concerned only with face‐to‐face school‐bullying, namely, bullying that occurs in schools between individuals, usually aged between 4 and 18 years old. In the school context, bullying is a complex social phenomenon, that often does not happen between the bully and victim in isolation (Salmivalli, 2010). For example, individuals can be involved in bullying, not only as bullies, victims, or bully‐victims, but also as bystanders, defenders, or reinforcers (Zych et al., 2017).

Cyberbullying is another form of aggressive behaviors that may occur within a school community, and previous research has found a significant overlap between offline (i.e., school‐bullying or face‐to‐face bullying) and online bullying (Baldry et al., 2017). There is currently very little information about the effectiveness of intervention programs designed to reduce cyberbullying or whether school‐based programs that also target face‐to‐face bullying can impact online bullying concurrently.

### 2.2 The importance of addressing school bullying

School‐bullying is a strong risk marker for several negative behavioral, health, social, and/or emotional problems. A recent comprehensive review of systematic reviews highlighted that the impact of school‐bullying can occur concurrently with perpetration and/or victimization, but also later in life (Zych et al., 2015). Previous studies have found that bullying victimization is often followed by negative mental health outcomes such as: increased suicidal ideation (e.g., Holt et al., 2015); generalized or social anxiety, low self‐esteem and loneliness (e.g., Hawker & Boulton, 2000); psychotic symptoms (e.g., van Dam et al., 2012); depression (e.g., Ttofi et al., 2011a, 2011b); sleeping problems (Geel et al., 2016); and other psychosomatic symptoms (Gini & Pozzoli, 2013).

Bullying perpetration, on the other hand, has been linked to several negative outcomes such as: suicidal ideation and suicidal attempts (Holt et al., 2015); weapon carrying (Valdebenito et al., 2018); drug use (Ttofi et al., 2016); and violence and offending in later life (Ttofi et al., 2011b, 2012). Although involvement in school bullying is not necessarily a causal factor for undesirable life outcomes, research has found that there is an apparent association. It may be the case that the experience of school bullying functions as a stepping stone toward undesirable life outcomes (Arseneault et al., 2010).

Moreover, involvement in school bullying, as either a bully or a victim, has been found to correlate with factors such as low academic achievement (Strøm et al., 2013), truancy from school (Gastic, 2008), and drug use (Valdebenito et al., 2015). Such factors are common risk factors for youth offending and delinquency (Farrington & Welsh, 2008). Therefore, a bullying prevention program could serve as a crime prevention program, as well as a form of promoting public health.

## 3 OBJECTIVES

It is clear that school bullying is an important target for effective intervention and prevention. Bullying is an ethical problem as well as a developmental one: targeting school bullying facilitates the process of optimal psychological development but it also addresses the question of human rights, especially the rights of the child (Sercombe & Donnelly, 2013). The aim of this paper is to provide an up‐to‐date systematic and meta‐analytical exploration of the effectiveness of school‐based antibullying programs. As such, the present report updates an earlier systematic and meta‐analytic review (Farrington & Ttofi, 2009; Ttofi & Farrington, 2011), by including evidence from an earlier report, and all available evaluations of antibullying programs since 2009.

It is hoped that this new evidence base will assist policy‐makers and practitioners working in the field of bullying prevention. Farrington and Ttofi's (2009) review concluded that school‐based antibullying programs are effective in reducing both bullying perpetration (OR = 1.36; 95% CI: 1.26–1.47; *z* = 7.86; *p* < .0001) and bullying victimization (OR = 1.29; 95% CI: 1.18–1.42; *z* = 5.61; *p* < .0001). Their review had a major impact on the field of bullying intervention and prevention, and in the 9 years that have passed since its publication there has been a wealth of new research.

Therefore, the aim of the present report is to conduct systematic searches for new evaluations of antibullying programs, and also update earlier analysis by including their 53 evaluations.

## 4 METHODS

The initial stage of any meta‐analysis involves conducting a thorough and systematic search of all the existing and relevant literature (Lipsey & Wilson, 2001; Littell et al., 2008). Using predetermined keywords and strict inclusion/exclusion criteria, a systematic review aims to identify, screen, appraise, and synthesize all relevant empirical studies (Zych et al., 2017). In this way, systematic bias is avoided.

### 4.1 Inclusion and exclusion criteria

To be included in the present systematic review, a set of strict inclusion and exclusion criteria were employed to guide searches. These criteria were identical to those used in the previous meta‐analysis (Farrington & Ttofi, 2009). Specifically, to be included, primary studies must:

(1) Describe an evaluation of a school‐based antibullying program implemented with school‐age participants (depending on the site of evaluation, ages may vary between 4 and 18 years of age);
(2) Utilize an operational definition of school‐bullying that coincides with existing definitions (e.g., CDC, 2014; Farrington, 1993; Olweus, 1993);
(3) Measure school‐bullying perpetration and/or victimization using quantitative measures, such as, self‐, peer‐, or teacher‐report questionnaires; and
(4) Use an experimental or quasi‐experimental design, with one group receiving the intervention and another (control group) not receiving the intervention. Nonrandomized studies had to measure outcomes before and after the intervention.

As a result, the present systematic review excludes studies that evaluate the effectiveness of intervention programs targeting alternative forms of bullying, such as cyber‐bullying (e.g., Del Rey et al., 2015), general aggression (e.g., Leff et al., 2010), and school violence (e.g., Giesbrecht et al., 2011). Other studies were excluded because they measured bullying‐related nonbehavioral outcomes, for example, “attitudes towards bullying” (e.g., Earhart, 2011), or coping strategies for dealing with victimization (e.g., Watson et al., 2010).

In addition, studies conducted with special needs, delinquent, or psychiatric populations were excluded (e.g., Espelage et al., 2015), so that results could be generalizable to the wider mainstream school population. Studies using qualitative measures of effectiveness, such as participant perceptions of the effectiveness of the program (e.g., Fletcher et al., 2015), were also excluded.

### 4.2 Searches^1^

In order to identify potentially includable studies, Boolean searches were conducted using multiple combinations of the following keywords: bully*; victim*; bully‐victim; school; intervention; prevention; program*; evaluation; effect*; and *anti‐bullying*. A full description of the syntax used is provided in Appendix A.

Searches were conducted on several online databases, including, but not limited to: Web of Science,^2^ PsychINFO, EMBASE, DARE, ERIC, and Scopus. Google scholar (www.scholar.google.co.uk) was also searched. A full list of databases searched is provided in Table 1. EBSCOhost was used as a platform to search multiple databases concurrently and such databases are indicated in Table 1.

**Table 1:** Online platforms and databases manually searched

1. British Education Index*
2. Cochrane Controlled Trials Register
3. Criminal Justice Abstracts*
4. Database of Abstracts of Reviews of Effectiveness (DARE)*
5. Educational Resources Information Clearinghouse (ERIC)*
6. EMBASE*
7. Google Scholar
8. MEDLINE*
9. National Criminal Justice Reference Service (NCJRS)*
10. ProQuest
11. PsychINFO*
12. Scopus
13. Web of Science

*Note:* EBSCOhost was used as a platform to search multiple databases concurrently. Such databases are marked with an *.

Databases of unpublished reports (e.g., ProQuest Dissertations and Theses Solutions) were also searched to include gray literature in our review. This should help to minimize potential publication bias linked to larger or significant effect sizes (Easterbrook et al., 1991; McAuley et al., 2000). In addition, evaluation studies included by previous systematic reviews were scanned, based on the name of each program, for additional‐updated evaluation results (i.e., Cantone et al., 2015; Chalamandaris & Piette, 2015; Evans et al., 2014; Jiménez‐Barbero et al., 2012, 2016).

Studies included in the previous review (Farrington & Ttofi, 2009; Ttofi & Farrington, 2011), were also included in the present systematic review. Searches for the present review were conducted up to the end of December 2016,^3^ for empirical studies published during and since 2009.

### 4.3 Screening

Our searches of the literature produced approximately 19,877 reports that were screened for eligibility. Based on the title and abstract, a total of 474 primary studies were identified as relevant, were obtained and subjected to further screening. Studies were allocated to six categories based on their relevance to the current meta‐analysis. A description of each category is provided in Table 2. Screening was undertaken by the first author (H. G.), under the supervision of the second author (M. T.), in a collaborative format. H. G. reviewed eligible studies, and any queries were settled in discussion with M. T.

**Table 2:** Relevance scale categories used in screening

Category name	Description
Category 1: Theoretical (minor)	Studies were primarily cross‐sectional or experimental explorations of factors, constructs or concepts relating to bullying and/or bullying prevention and intervention and implications of findings are discussed in relation to research/development/future antibullying programs
Category 2: Theoretical (weak)	These studies focused more on antibullying programs specifically, either by providing an overview of their effectiveness, theory or implementation or systematically reviewing existing evaluation studies
Category 3: Descriptive	Studies provided an overview, narrative description of a specific antibullying program or bullying intervention/prevention strategy, however, no evaluation of the effect of implementing the program is presented
Category 4: Not included (strong)	These studies were more relevant to the present review, however, were excluded because they either had methodological issues, the outcomes were not related to a change in actual bullying behaviors (e.g., outcomes related to attitudes toward bullying), or measures related to a construct other than school bullying (i.e., cyberbullying, peer victimization, or peer aggression)
Category 5: Strong and included	These were evaluation studies of antibullying programs that met all the inclusion criteria for the current review

The initial wave of screening excluded 258 of these primary studies. At this stage, studies were excluded because they: (1) did not evaluate a specific antibullying program (Category 1; *n* = 107); (2) reviewed several different antibullying programs (Category 2; *n* = 108); or (3) did not report empirical quantitative data from an evaluation of a specific antibullying program (Category 3; *n* = 43).

**Figure 1:**
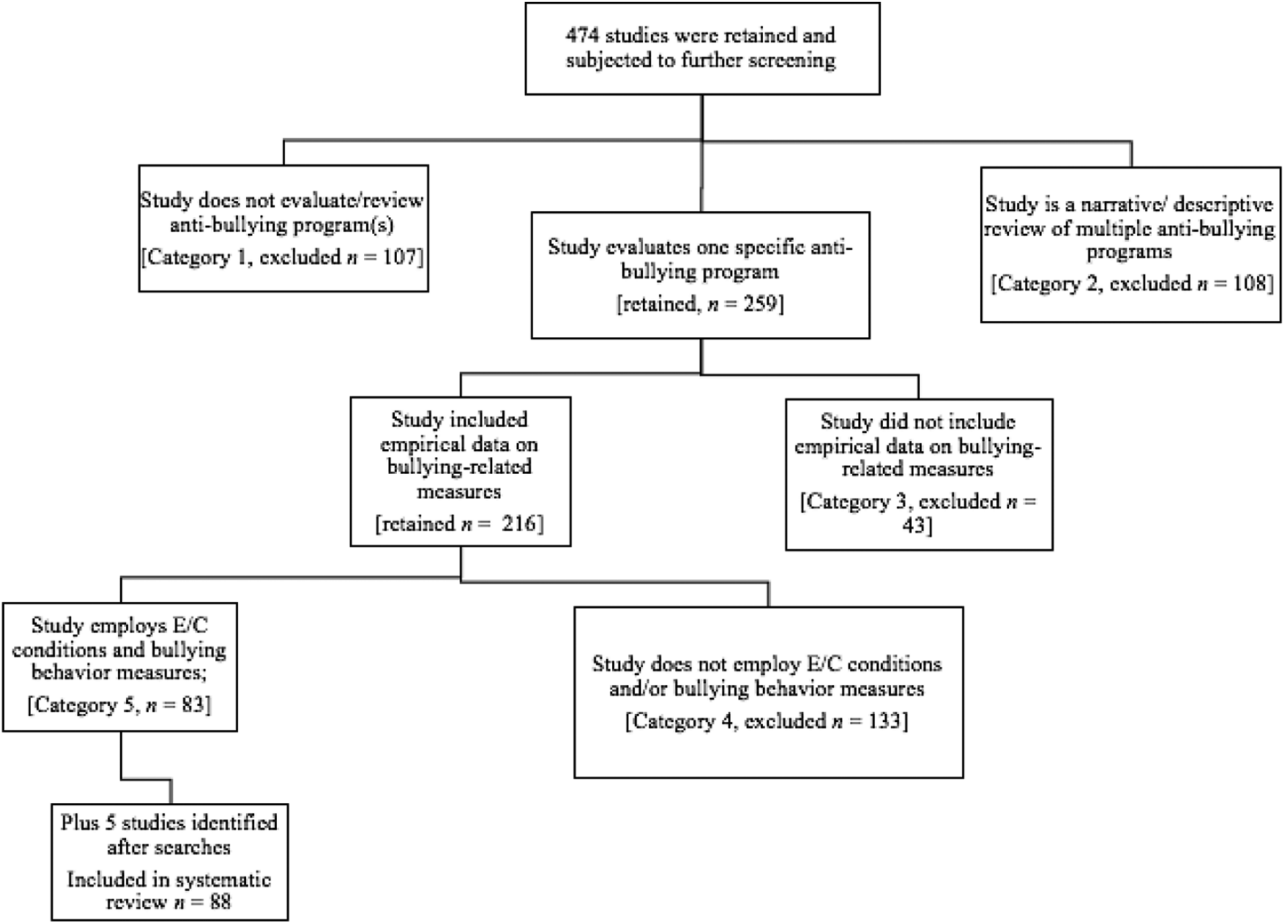
Screening of studies

A second wave of screening excluded a further 133 studies (Category 4; see Table 3). Primary studies were excluded at this stage because they: (1) reported irrelevant outcomes; (2) did not have an adequate control group; or (3) did not meet specified methodological criteria. The screening process is described in detail in Figure 1. In total, 83 studies published since 2009 were included in our updated systematic review (Category 5).

**Table 3:** Descriptions of category four studies

Study	Reasons for exclusion from meta‐analysis
Ahtola et al. (2013)	Explore teachers' perceptions of support from schools' principals in the KiVa program, and whether this predicted implementation adherence. Did not compare bullying outcomes of program
	*[Outcomes]*
Ahtola et al. (2012)	Examined the effects of the KiVa antibullying program on teacher perceptions of bullying, no outcome of bullying behaviors in students is included
	*[Outcomes]*
Al‐Samarri (2011)	Evaluated the effectiveness of the “Mythodrama” violence prevention program, on verbal and physical bullying, but did not employ a control group
	*[No control group]*
Azad and Amiri (2012)	Carried out an evaluation of the Olweus Bullying Prevention Program in a randomized controlled trial with Iranian primary school boys, however only abstract was published in English and did not provide enough details for meta‐analysis
	*[Other: Language]*
Allen (2010)	Evaluated a whole‐school bullying intervention initiative for the effectiveness in reducing bullying, however, did not employ a control group for comparison
	*[No control group]*
Amundsen and Ravndal (2010)	Assessed the effectiveness of the OBPP to reduce alcohol and substance use in adolescents, but no measure/outcome of bullying behaviors actually employed
	*[Outcomes]*
Athanasiades et al. (2015)	Evaluated the “Tabby Project,” a program designed as a prevention and intervention program for cyberbullying among adolescents. While measures of traditional bullying and victimization were also included, but only as predictors/correlates of cyber‐bullying and victimization. The evaluation data presented refers only to the effects of the intervention program on cyberbullying behaviors
	*[Outcomes; Cyberbullying]*
Beckman and Svensson (2015)	Evaluates the cost effectiveness of the Olweus Bullying Prevention Program, not the effectiveness of the program to reduce bullying
	*[Method]*
Beets et al. (2009)	Conducted and evaluated an intervention program for Hawaiian elementary‐school students for a number of outcomes, including violent behaviors, but no outcomes relevant to school bullying
	*[Outcomes]*
Beightol et al. (2012)	Re‐publication of Beightol et al. (2009). This report evaluates treatment effects on participant goals, empathy, self‐efficacy and resilience. Only qualitative data refers to bullying outcomes. Employed the “Anti‐bullying Initiative Survey” which does include six items regarding bullying behaviors, however did not administer this section
	*[Outcomes]*
Beightol et al. (2009)	Evaluates the effectiveness of an adventure‐based intervention, but main outcome is participants' “resilience,” implications for reducing bullying, but provide no empirical evaluation data
	*[Outcomes]*
Boulton (2014)	Conducted an evaluation of the teacher‐training component of the I DECIDE antibullying program, and its effectiveness at increasing teachers' perceived effectiveness, self‐efficacy and implementation of the program. Implications for the impact of the program on bullying are discussed, however no direct evaluation is conducted
	*[Outcomes]*
Bowes et al. (2009)	Conducted a process evaluation of the “Peers Running Organized Play Stations (PROPS)” intervention program. Outcome of interest was the implementation rate of the program by teachers, not the effect of the program on bullying behaviors
	*[Outcomes]*
Brenick et al. (2014)	Evaluation of a safety‐skills program for elementary school children. Study did include a measure of victimization, however only the outcome “safety skills knowledge” was analyzed pre‐ and posttest as an indicator of the effectiveness of the program. Additionally, the victimization measure refers to “participants' perceptions of the regularity of bullying…” and not their actual experiences of being victimized
	*[Outcomes]*
Bundy et al. (2011)	Evaluation of a program to develop physical and social skills in children who are overweight. Main aim of program was to increase physical activity levels of children, and authors suggest that such outcomes would decrease childhood obesity and as a result, bullying. However, do not employ any bullying‐related outcome measures to assess the impact of the program on bullying experiences/behaviors directly
	*[Outcomes]*
Burkhart et al. (2012, 2013)	Evaluation of a community‐based family violence intervention and prevention program, that included parent‐measures of early childhood bullying. However, was excluded because bullying measures were not specific enough to school bullying
	*[Outcomes]*
Cecil and Molnar‐Main (2015)	Explored the effect of implementer (e.g., teachers) characteristics, beliefs of self‐efficacy, and perceptions and attitudes toward bullying on OBPP implementation and fidelity
	*[Outcomes]*
Cerni Obrdalj et al. (2014)	Conducted an evaluation of a violence prevention program which involved family physicians (GPs). Included a measure of “frequency of experiencing violence at school,” however did not employ a control group to compare effect
	*[No control group]*
Chu et al. (2013)	Tailored intervention of victims of bullying suffering from anxiety and depressive disorders. Measures included a scale measuring impairment (on family/peer relations and academic performance) that occurs as a result of bullying. Outcomes of effectiveness are changes in psychological clinical symptoms as a result of victimization, and participant satisfaction with the intervention. No change in victimization is reported
	*[Outcomes]*
Cobb (2009)	Investigated the effectiveness of Disciplinary Alternative Education Programs (DAEPs) for improving academic performance of students who demonstrate challenging behaviors, for example, those that bully others
	*[Outcomes]*
Cooke et al. (2007)	Examined the impact of the violence prevention program “Second Step” on a number of outcomes, including bullying behaviors, measured by four items on the Modified Aggression Scale, did not employ experimental and control conditions
	*[No control group]*
Cornell et al. (2009)	Explore differences between schools that implement a violence prevention set of guidelines on constructs such as bullying, but no pre‐ and posttest measures, is a “nonexperimental” study
	*[Method]*
Cross et al. (2015)	Evaluation study of the “Cyber Friendly Schools” program for the prevention and intervention of cyberbullying. Outcome measure specifically refer to cyberbullying, no measure of traditional/offline bullying included
	*[Outcomes; Cyberbullying]*
Cross et al. (2012)	Report the results of a 3‐year evaluation study of the Friendly Schools, Friendly families program, however no control group is utilized as after the 2nd year of implementation, many schools wished to implement the program. Authors compare the effectiveness of the program across three different levels of implementation, low, moderate, and high
	*[No control group]*
Daugherty (2011)	Aimed to evaluate the effectiveness of the Olweus Bullying Prevention Program, but the main outcome of interest were teacher and school principals' perceptions of the effectiveness of the program. Survey does include an item referring to a decrease in bullying incidents, however, this is related to teacher and principal perceptions and opinions about whether or not bullying decreased, rather than actual records indicating they did
	*[Outcomes]*
Davis (2011)	Abstract outlines that the study evaluated the effectiveness of a social skills treatment program for children displaying problems behaviors such as bullying, aggression, and poor social skills. However, do not evaluate the program's effectiveness of altering these problem behaviors. Instead, assess the change in variables such as empathy, social skills, and motivation
	*[Outcomes]*
Del Rey et al. (2015)	Evaluation study of the cyberbullying intervention and prevention program “ConRed” specifically on cyberbullying behaviors. Thus, excluded from the present review as no measures of school bullying were employed
	*[Outcomes; Cyberbullying]*
DeNike (2014)	Abstract outlines that the report evaluated the effectiveness of just one part of the “No Bully System” antibullying intervention, the Solution Team, limited information is available, but do not refer to a comparison group in graphical representation of findings
	*[No control group]*
Dogini (2012)	Conducted a qualitative study to explore teacher and school staff opinions about the effectiveness of an antibullying intervention program
	Dissertation, only preview available
	*[Method]*
Drury (2014)	Investigated whether an antibullying program reduced “HIB” incidents (i.e., harassment, intimidation and bullying). Do not compare effect of intervention with a control group
	*[No control group]*
Earhart (2011)	Investigated the effect of implementing the “Promoting Positive Peer Relationships” program, however excluded as effectiveness of the program was measured using attitudinal outcomes of bullying rather than bullying behaviors
	*[Outcomes]*
Emfield (2015)	Evaluated the experiences of participants in an antibullying self‐defense training program. Qualitative data only about the participants' opinions and thoughts on the program, no quantitative measure of bullying outcomes
	*[Outcomes; Method; No control group]*
Espelage et al. (2015)	Randomized clinical trial of the Second Step: Student Success Through Prevention program in middle schools to reduce bullying. However, excluded from present review as sample utilized were disabled
	*[Sample]*
Farmer et al. (2010)	Conducted an evaluation of the “Rural Early Adolescent Learning Program (Project REAL), to explore the impact of the program on teachers” abilities to identify peer groups among their students and also identify the incidents of bullying occurring in peer groups
	*[Outcomes]*
Farrell et al. (2015)	Qualitatively explored participants in the “Second Step” violence prevention programs' implementation and perceptions of the skills they learnt during the program. No measure of actual bullying behaviors or victimization is utilized
	*[Outcomes; Method]*
Fletcher et al. (2015)	A qualitative study evaluating the implementation of an antibullying program, specifically, how young were involved and young peoples' experiences of the program
	*[Method]*
Frost (2012)	Examined the prevalence of school programs implemented in Kansas, including, bullying prevention, conflict resolution and peer mediation programs. Compare official records of school suspension for violence in relation to the type of program implemented. However, do not use any indicator of specific school bullying perpetration or victimization
	*[Outcomes]*
Fung (2012)	Tested the effects of an intervention with high‐risk reactive aggressors (i.e., bullies) over five time‐points in 1 year, however no control group was utilized
	*[No control group]*
Garandeau, Poskiparta, et al. (2014)	Using data from a previous evaluation study of the KiVa antibullying intervention program, the authors compared the impact of the “Confronting” and “Non‐Confronting” approaches on bullying victimization. Thus, compare intervention participants according to which arm they were assigned to, but do not compare either with control group
	*[No control group]*
Gibson et al. (2014)	Evaluates the outcomes of a bullying‐focused program, refer to outcomes such as fear of bullying and peer/teacher interventions in bullying
	*[Outcomes]*
Giesbrecht et al. (2011)	WITS violence prevention program, reduced levels of physical and relational victimization. Excluded because outcome variables are not specific enough to school bullying
	*[Outcomes]*
Goncy et al. (2015)	Investigates the influence of several aspects of teacher implementation of the OBPP, such as: *adherence; competence; and student responsiveness*, on student engagement with the intervention, not any change in their bullying behaviors as a result of the program
	*[Outcomes]*
Good et al. (2011)	Report presents a case study example of a school in Canada that implemented the “School Wide Positive Behavior Support” Program, using discipline referrals for bullying as an effectiveness indicator. However, do not employ a comparison school as a control
	*[No control group]*
Green (2015)	Examined the differences between discipline referral rates and academic performance before and after a schools' implementation of the Olweus Bullying Prevention Program. However, do not utilize a control school
	Dissertation, only preview available
	*[No control group]*
Gregus et al. (2015)	Describe two separate studies that tested the effects of a Lunch Buddy mentoring program. First study was with victimized elementary school children, and the second was with bully‐victim children. Excluded due to lack of control group
	*[No control group]*
Greytak and Kosciw (2010)	Present the results of a 1 year training program “Respect for All” for secondary school teachers in order to increase their abilities to intervene and be aware of LGBT bullying in their schools. Evaluated the effectiveness of the program for teachers' attitudes toward LGBT students and various variables relating to their self‐efficacy beliefs to intervene, but not on actual bullying behaviors of their students
	*[Outcomes]*
Greytak et al. (2013)	Evaluate a professional development program for teachers that aims to help them to develop better strategies and attitudes toward LGBT youth and prevent bullying. Do not evaluate the outcomes of this program in relation to actual bullying incidents in schools. Focus instead on teacher‐related outcomes, similar to Greytak and Kosciw (2010)
	*[Outcomes]*
Gyooyeong (2013)	Evaluated the effectiveness of a program designed for victimized adolescents. Looked at changes in ego‐resiliency, self‐esteem, somatic symptoms, aggression and social withdrawal in intervention and control group, but change in bullying behaviors/experiences was not an outcome
	*[Other; Language]*
Haataja et al. (2014)	This study evaluates the link between implementation fidelity of the KiVa antibullying program and its outcomes, do not actually explore the effectiveness of the program as a whole
	*[No control group]*
Hallam (2009)	Qualitative aspect of the evaluation of school staffs' (i.e., teachers, principals and nonteaching staff) perceptions of the effectiveness of the Social and Emotional Aspects of Learning program (SEAL) on a range of outcomes, including bullying. Quantitative student measures include measures of emotional and behavioral skills, perceptions of classroom and school ethos and their attitudes toward school, but not bullying behaviors
	*[Method (Teacher‐report); Outcomes (Student‐report)]*
Harshman (2014)	Mixed method study that explored differences in student perceptions of Internet safety and cyberbullying before and after participating in the “i‐SAFE” internet safety program. No outcomes of traditional bullying are employed
	*[Outcomes; Cyberbullying]*
Hatzenbuehler and Keyes	Evaluated the impact of antibullying policies that incorporate an antihomophobic element on suicide and attempted suicide in homosexual adolescents. However, do not explore the impact of these policies on reported bullying behaviors
	*[Outcomes]*
Hawe et al. (2015)	Replicated the Gatehouse project intervention in Canadian schools, and investigated the effects of program on a series of health risk behaviors, including bullying victimization. Excluded due to lack of inclusion of a control group
	*[No control group]*
Hervey and Kornblum (2006)	Evaluation of a violence prevention program, “Disarming the Playground,” on a variety of different outcomes. The behavioral measure included does include some aggressive items, but these are not specified as being related to bullying behaviors
	*[Outcomes]*
Hoglund et al. (2012)	Evaluated effectiveness of a community‐based, whole‐school prevention program “WITS Primary Program” for peer victimization. However, victimization measures are not specifically related to school bullying, thus, excluded from the current review
	*[Outcomes]*
Holden (2015)	Evaluated the effectiveness of the Olweus Bullying Prevention Program
	However, excluded from the present meta‐analysis as did not include a control group for comparison
	*[No control group]*
Hornblower (2014)	Evaluated an antibullying program implemented in an English secondary school, but did not include a control condition
	*[No control group]*
Huddleston et al. (2011)	Describe the implementation and evaluation of an individualized intervention for one adolescent middle school bully and investigated the impact on their bully behaviors, however no control student/group
	*[No control group]*
Hutchings and Clarkson (2015)	Presents results from the pilot implementation of the KiVa antibullying program in the UK. However, do not employ any control condition in order to evaluate the significance of any results
	*[No control group]*
Isaacs (2009)	Examined the impact of the OBPP in U.S. middle schools, however conduct a “single school” study, and thus, did not include a control school
	*[No control group]*
James (2011)	Conducted cross‐cultural comparisons of the effect of peer support approaches to bullying prevention. In two studies conducted in UK, compare quantitative measures of bullying as a result of program. Excluded on the basis that no control condition was employed
	*[No control group; Method]*
James et al. (2011)	Evaluation of an educational program to raise awareness of relational aggression/bullying in teenage girls, however, knowledge and attitudes of relational bullying and change in these constructs were the primary outcome of interest
	*[Outcomes]*
James et al. (2013)	Evaluated the applicability of the relational aggression educational program implemented by James et al. (2011), for boys, but main focus is knowledge and attitudes toward relational bullying
	*[Outcomes]*
Jeong‐Lan and Oh‐Hyun (2014)	Evaluated a school violence prevention program and its effectiveness to increase levels of empathy in school children. Do not refer to any bullying‐related outcomes
	Full text only available in Korean
	*[Outcomes; Other: Language]*
Jiminez‐Barbero et al. (2013)	Explored the effects of a school violence prevention program on a range of outcomes, such as attitudes toward violence and perceived violent victimization. Imply modifying attitudes toward violence can reduce prevalence of bullying, but no bullying measure
	*[Outcomes]*
Knights (2011)	Conducted an evaluation of the impact specialized schools for highly victimized adolescents, “Red Balloon Learner Centers.” However, the evaluation outcomes are clinical and academic‐related constructs, such as levels of anxiety/depression in RBLC participants and victimized children from Local Authority comparison schools. The only bullying‐related measure is concerned with establishing retrospective bullying experiences, and the severity of past bullying experiences
	*[Outcomes]*
Konishi et al. (2013)	Explored the association between schools implementing antihomophobic bullying policies and LGBT youths' alcohol and drug use, however, do not investigate the effect of these program on bullying/victimization experiences
	*[Outcomes]*
Langevin et al. (2012)	Examined the effects of an antibullying program specifically targeting bullying of children who have a speech impediment. Assess change in attitudes toward and knowledge of this type of bullying. Authors did conduct a measure of bullying behaviors, but only at pretest baseline. Thus, the effect of the intervention on bullying behaviors cannot be assessed
	*[Method]*
Layfield (2014)	An exploratory case study of one school's implementation and methods for reducing problem behaviors, such as bullying. No control school utilized
	Dissertation, only preview available
	*[No control group]*
Leadbeater and Sukhawatanakul (2011)	Evaluated the effect of the WITs program on elementary school children to reduce peer victimization trajectories. However, victimization outcomes do not relate to school bullying
	*[Outcomes]*
Leff et al. (2010)	Evaluates a program designed to reduce relational aggression in schools, discuss implications for bullying prevention in text, but main outcome is aggression
	*[Outcomes]*
Low et al. (2014)	Using data from a previous evaluation of the *Steps to Respect* program (Brown et al., 2011), this study assessed the predictors of implementation factors such as: engagement and adherence. Bullying victimization and perpetration are included as possible indicators, but the study does not compare these measures in relation to the effectiveness of the intervention
	*[Outcomes]*
Lucassen and Burford (2015)	Evaluated a sexuality diversity workshop in secondary schools and its potential impact to reduce school bullying. The effect of the program is primarily assessed through changes in participants valuing and understanding of sexually‐diverse individuals, no actual measure of bullying experiences utilized
	*[Outcomes]*
Macedo et al. (2014)	Implemented an evaluated the program “We are the Others” in a group of Portuguese students, did not employ a control group
	*[No control group]*
Malatino (2011)	Conducted an evaluation of the program “City Connects” on a range of social development outcomes, including bullying behaviors. However, no true control group is utilized. All participants had been exposed to the intervention, just at different “dosage” levels, that is, for longer/shorter periods of time
	*[No control group]*
McElearney et al. (2013)	Examined the effectiveness of a school counseling intervention in improving peer relationships in children identified as victims of bullying. Measures included the Strengths and Difficulties Questionnaire, and the Peer Problems subscale, but no direct measure of bullying behaviors/experiences utilized
	*[Outcomes]*
Mendes (2011)	Examined the effects of an antiviolence school program on the levels of bullying in a Lisbon school, however do not include a control condition
	*[No control group]*
Menesini and Nocentini (2012)	Conducted an evaluation of the efficacy of a peer‐led intervention program to reduce cyberbullying perpetration and victimization. Authors do not include any measures of traditional/offline bullying
	*[Outcomes; Cyberbullying]*
Migliaccio and Raskauskas (2013)	Evaluated a small‐scale video‐based bullying awareness program, but the main outcomes were changes in knowledge about and attitudes toward bullying behaviors and no measure of actual bullying behaviors was employed
	*[Outcomes]*
Minton et al. (2013)	Implemented and evaluated an antibullying intervention described as a “whole school/community development” program in Ireland primary and post primary schools on self‐reported involvement and experiences of bullying. Excluded due to lack of control condition
	*[No control group]*
Miyari (2013)	Implemented and evaluated a weight‐related “teasing” (or bullying) prevention program, but did not employ any control group
	*[No control group]*
Nakamura and Koshikawa (2014)	Conducted an evaluation of a social skills training and psychoeducational program for preventing bullying in Japan, however the full text was not available in English
	*[Other: Language]*
Nese et al. (2014)	Evaluated the Expect Respect intervention program, using a nonconcurrent multiple baseline design. All participants received the intervention, thus, no control group was used for analysis
	*[No control group]*
Newgent et al. (2010)	Carried out an evaluation of a psychoeducational program in order to determine the effect on several outcomes, including bullying behaviors. However, comparison groups were formed on the basis of pre‐test clinical symptoms, and all students received the intervention, thus, no true control group employed
	*[No control group]*
Newgent et al. (2011)	Conducted an evaluation of the teacher‐training elements of the “Bully Busters” universal prevention program. Effectiveness of the program was assessed by outcomes including teacher efficacy, skills and knowledge concerning peer victimization, and also their reports of students' peer victimization
	Full text unavailable, so assuming that is to be excluded because outcomes are peer victimization, not bullying
	*[Other: Unavailable]*
Nixon and Werner (2010)	Evaluation of the intervention program “Creating a Safe School” (The Ophelia Project) to reduce relation aggression and victimization in children. Thus, “relational aggression” and “relational victimization” are the primary outcomes, not specifically related to bullying
	*[Outcomes]*
Pack et al. (2011)	Conducted an evaluation of the Safe School Ambassadors program me, however outcomes of interest are participants' perceptions of the impact of the project. Did not employ a direct measure of actual bullying experiences
Park et al. (2014)	Effects of a “food‐therapy” program on bullying/school violence (crossover between terms used in Abstract)
	Full text in Korean
	*[Other: Language]*
Peagram (2013)	Evaluated the impact of the Bulldog Solution Intervention Model as a way to reduce bullying and aggression and increase empathy, and self‐esteem. However, measure of bullying is inadequate, student measure relates to being a bystander or witness to bullying ("I have seen bullying")
	*[Outcomes]*
Pepler and Craig (2011)	Do not directly evaluate the effectiveness of a specific antibullying intervention or prevention program. Authors examine the effects that the establishment and work of the “Promoting Relationships and Eliminating Violence Network (PREVNet)” Canadian research network has had on research on bullying and participation in antibullying initiatives
	*[Method]*
Phillips (2015)	Implemented a bullying prevention program in order to ascertain its effectiveness in changing educators' perceptions of bullying, thus, the main outcome evaluated was not bullying behaviors by students. Additionally, did not employ a control group
	Dissertation, only preview available
	*[No control group; Outcomes]*
Pister (2010)	Evaluated the “Working against Youth Violence Everywhere” program to prevent bullying and violence in schools, however unable to obtain full text
	*[Other: Unavailable]*
Poindexter (2015)	Implemented a short altruism‐based educational intervention to reduce bullying‐related attitudes and behaviors. Outcome measures of behaviors however, are defined as “pro‐social behavioral intentions,” and not actual engagement in, or experience of, bullying
	Dissertation, full text unavailable
	*[Outcomes]*
Ramierz and Lacasa (2013)	Conducted an evaluation of an antibullying program in Spanish primary schools but did not employ a control group
	Full text in Spanish
	*[No control group]*
Renshaw and Jimerson (2012)	Examined the impact of a bullying prevention curriculum for middle school students, however, effectiveness outcomes do not refer to bullying behaviors, but attitudes toward bullying and perceptions of bullying‐related support services within the school
	*[Outcomes]*
Rigby and Griffiths (2011)	Qualitative evaluation data from interviews with students and practitioners involved in the antibullying initiative “Method of Shared Concern” are reported, but there was no quantitative evaluation of effectiveness of program
	*[Method]*
Roberto et al. (2014)	Evaluated the effects of the “Arizona Attorney General's Social Networking Safety Promotion and Cyberbullying Prevention” presentation on cyberbullying perpetration and victimization. No measures of traditional bullying are employed
	*[Outcomes; Cyberbullying]*
Ross and Horner (2014)	Investigated the effect of the “School‐Wide Positive Behavior Interventions and Supports,” and measures employed did include 9 items that refer to bullying perpetration and victimization, however did not employ a control group
	*[No control group]*
Ross (2009)	Evaluated the single‐subject program Bully Prevention in Positive Behavior Support to reduce bullying behaviors. However, do not employ a control group
	*[No control group]*
Ross and Horner (2009)	Journal publication of Ross (2009) dissertation
	*[No control group]*
Rubin‐Vaughan et al. (2011)	Evaluated the effect of the “Quest for the Golden Rule” e‐learning antibullying program, but outcomes were attitudes and knowledge of bullying issues and effective intervention and coping strategies
	*[Outcomes]*
Santos et al. (2011)	Investigated the impact of a school violence prevention program widely implemented in Canada, “Roots of Empathy,” but targeted outcomes are mental health or generic aggression/violence related and not specified to refer to bullying
	*[Outcomes]*
Saurini (2011)	Explored the effect of a psychoeducational anger management program on bullying behaviors, but do not utilize a control condition
	*[No control group]*
Savich (2014)	Evaluation of the effect that a change in state bullying and cyberbullying policy had on the states' schools' implementation of antibullying programs, reporting bullying to the education board and changes in bullying policies within schools. Do also refer to rates of reported bullying incidents in schools, and how they changed according to prevention and intervention programs, but is not clear as to whether this is a result of the actual programs implemented, or due to the policy change
	Dissertation, only preview available
	*[Other: Unavailable]*
Scheithauer and Bull (2010)	Imply that text presents the results of a pilot evaluation of the “fairplayer.manual” school bullying preventative intervention program on prevalence of bullying, however, no control group was employed
	*[No control group]*
Shek and Yu (2013)	Evaluation of the Project P.A.T.H.S, an intervention program in Hong Kong for adolescent males' risky behaviors. School bullying is not an outcome
	*[Outcomes]*
Spiel et al. (2012)	Qualitative evaluation study of Austria's national school violence prevention program
	*[Methods]*
Splett et al. (2015)	Describes evaluation of intervention program for reducing relational aggression, not specific to bullying
	*[Outcomes]*
Srekovic (2015)	Effectiveness of a social intervention program for students with Autism Spectrum Disorder who were identified as being bullied, or at risk of being bullied. Conducted a peer network intervention, however, did not employ any control or comparison group
	*[No control group]*
Stallard and Buck (2013)	Evaluated an intervention program where the main outcome was reducing depression in participants, thus, bullying experiences and behaviors were not the primary outcomes. Qualitative focus groups conducted after the interview did review participants' perceptions of bullying issues covered in the intervention
	*[Outcomes]*
Steiger (2010)	Assessed the effectiveness of the “Solution Team” antibullying program for primary school children identifying as victims of bullying, however do not employ a control group for comparison
	*[No control group]*
Tanrikulu et al. (2015)	Evaluated the “Sensitivity Development” program to reduce cyberbullying behaviors in adolescents, no measure or outcome of traditional bullying is included
	*[Outcomes; Cyberbullying]*
Tokarick (2015)	Evaluated the effect of bullying prevention program on adolescent females' perceptions of bullying, thus, not actual bullying behaviors
	*[Outcomes]*
Tomic‐Latinac and Nikcevic‐Milkovic (2010)	Evaluated the efficacy of the UNICEF bullying prevention program in high school students. However, full text is published in Croatian
	*[Other: Language]*
Toshack and Colmar (2012)	Study evaluated a psycho‐educational program that aims to reduce cyberbullying with female primary school students. No measure of traditional bullying was included
	*[Outcomes; Cyberbullying]*
Vanderheiden (2013)	Evaluation study of a large‐scale antibullying program, however, abstract does not provide much information and unable to obtain full text so was excluded on this basis
	*[Other: Unavailable]*
Vannini et al. (2011)	Investigated the impact of the “FearNot!” virtual antibullying program in UK and German schools on participants' “defender” status. Thus, indicator of effectiveness was an increase in peer‐reported bystander intervention, not decreases in reports of bullying behaviors
	*[Outcomes]*
Velderman (2015)	Evaluation of a professional development program for teachers, and the impact the development program had on their knowledge of bullying related issues and implementation of antibullying plans. Do not however, evaluate the effectiveness in reducing bullying behaviors among their students
	*[Outcomes]*
Watson et al. (2010)	Examined the efficacy of the FearNot! bullying prevention program in UK and German schools, comparison is done cross‐nationally. However, effectiveness outcome is coping strategy knowledge in relation to bullying victimization, not actual reports of being bullied
	*[Outcomes]*
Westheimer and Szalacha (2015)	Chapter outlining the Welcoming School program for LGBT antibullying. Do outline an evaluation study, but none of the outcomes relate to bullying perpetration or victimization
	*[Outcomes]*
Wolfe et al. (2012)	Evaluated the classroom‐based intervention program, the “Fourth R program” which aims to decrease abusive and health‐risk behaviors in adolescents. No outcome of bullying is included, “peer resistance skills,” that is, ability to withstand peer pressure is the primary targeted outcome. During intervention, one of the pressures adolescents are pressed to comply with is a bullying scenario
	*[Outcomes]*
Wölfer et al. (2014)	RCT evaluation of a cyberbullying prevention and program, “Media Heroes.” Outcome measures refer only to cyberbullying behaviors, do include a measure of “aggressive behaviors” but are not specific to school bullying
	*[Outcomes; Cyberbullying]*
Wood (2012)	Evaluate the “implementation fidelity” of the Olweus Bullying Prevention Program, however do not employ a control comparison group.
	*[No control group]*
Wright et al. (2012)	Investigated the effectiveness of a bullying intervention program, The Ophelia Project, but outcome measure was relational aggression, not bullying behaviors
	*[Outcomes]*
Yamashiro (2013)	Qualitative evaluation using semi‐structured interviews with participants in the Anti‐Bullying Prevention Pilot Program (ABPPP)
	*[Methods]*
Young et al. (2009)	Appears to evaluate a bullying prevention approach adopted by school counselors in one school. Effectiveness is measured using discipline referral rates, however no control group was employed
	*[No control group]*

In addition, five studies were identified during searches conducted for a meta‐analytical review of cyberbullying prevention programs (Gaffney et al., 2018). These studies were missed during systematic searches for the current review (i.e., Kaljee et al., 2017; Ortega‐Ruiz et al., 2012; Ostrov et al., 2015; Silva et al., 2016; Solomontos‐Kountouri et al., 2016). One of these studies (i.e., Kaljee et al., 2017) has a publication date outside of the range of our searches. However, it was included because it was available online in 2016.

To provide the most up‐to‐date analysis of school‐based bullying prevention and intervention programs, therefore, a total of 88 newly identified studies are included in the present systematic review.

## 5 DATA EXTRACTION

After identifying studies eligible for inclusion in the present systematic and meta‐analytical review detailed information about the antibullying programs, sample involved, and evaluation design were extracted from primary studies. The following chapter outlines the coding framework applied in greater detail.

Table 4 also outlines each piece of information extracted. Information was extracted from primary studies under four main headings: (1) Descriptives, (2) Design, (3) Program, and (4) Outcomes. Additionally, the following section outlines information extracted from primary studies in order to create a risk of bias index. Table 5 outlines the items utilized to assess risk of bias for each of the methodological designs included in the present report. Details of the risk of bias results for each study is provided in Appendix B.

**Table 4:** Coding framework

Type	Information extracted	Example
Descriptive	Sample size Age of sample in years Grade(s) of sample or range Sex: % female and % male Location or country Publication year Publication type	Total *N, n* experimental, *n* control Mean age/range 2009 versus 2016 Journal article, book chapter, dissertation, report
Design	Evaluation method Measures Data collection timepoints Unit of allocation/randomization N clusters Matched‐groups	RCT, BA/EC, or age cohort design Name of instrument Timeframe Perpetration/victimization/both Type of report Baseline/postintervention/follow‐up
Program	Name of program Intervention length Core components Intervention aim and/or target N workshops Location of intervention Conflict of Interest Specificity	For example, OBPP or KiVa Peer, parent, and teacher involvement Involvement of external stakeholders Intervention activities Curriculum/structure/nonstructured High, low, possible risk High, low, medium specificity
Outcomes	Bullying at baseline for exp and control Bullying post‐intervention for exp and control Independent samples Type of outcome Multiple measures	Mean, *SD, N* N and % bullies and/or victims

Abbreviations: BA/EC, quasi‐experiments with before and after measures of bullying (nonrandomized); exp, experimental group; OBPP, Olweus Bullying Prevention Program; RCT, randomized controlled trial.

**Table 5:** Risk of bias tool

Name	Design	Risk category	Criteria
*EPOC tool items*			
Allocation sequence	RCTs	Low	Random component in sequence generation process is described (e.g., used a random number table)
		High	A nonrandom method is used (e.g., date of agreement to participate)
	BA/EC	Low	Matched‐pairs design used; units could not be randomized due to lack of specific intervention‐related resources (e.g., computer access) beyond evaluator control
		High	Unmatched design used or unit allocated as a result of specific request due to increased levels, or perceived high levels, of bullying. Units could not be randomized due to failure of schools to agree to participation if in control group/would be randomly assigned to condition
	AC	Low	No age cohorts were categorized as low‐risk, due to the nature of allocation to experimental and control conditions
		High	All age cohorts were categorized as high risk on this item, due to the nonrandom nature of allocation
Allocation concealment	RCTs	Low	Random allocation was conducted by external body; research team; or prior to screening, or after consenting to participate; Allocation was communicated using sealed envelopes
		High	Random assignment was managed by schools themselves; randomization occurred after participant screening; allocation was randomized prior to consent to participate, and was communicated to schools in information sheet
	BA/EC	Low	Schools were asked to agree to participation before being allocated to experimental or control condition
		High	Schools were asked to agree to participate *after* being told the experimental condition they were assigned to; schools specified they would participate on the basis of being allocated to a specific condition
	AC	Low	No age cohorts were categorized as low‐risk, due to the nature of allocation to experimental and control conditions
		High	All age cohorts were categorized as high risk on this item, due to the nonrandom nature of allocation
Baseline equivalence	ALL	Low	Baseline levels of bullying in experimental and control groups is reported and no significant differences are found; means and distribution of bullying is similar between experimental and control groups at baseline
		High	Baseline levels of bullying in experimental and control groups is reported and significant differences are found; means and distribution of bullying are different between experimental and control groups at baseline
Baseline characteristics	ALL	Low	Balance in participant demographics between experimental and control groups at baseline; matched pairs of units of allocation
		High	Imbalance in participant demographic between experimental and control groups at baseline; no information of baseline characteristics of participants is reported
Incomplete data	ALL	Low	Zero attrition is reported; attrition represents a low percentage of cases; missingness was equivalent across experimental and control groups; attrition was reported and an adequate strategy to deal with attrition was applied
		High	High percentage of attrition reported and no strategy to deal with attrition mentioned; list‐wise deletion was used to respond to attrition; attrition impacted the experimental and control groups unequally
Blind outcome assessment	ALL	Low	Individuals who were independent of intervention implementation collected outcome data; individuals collecting data were unaware of experimental condition
		High	Individuals who implemented intervention administered outcome measurement instruments; if individuals collecting data were aware of experimental condition or if observers in observational data were aware of experimental condition
Contamination protection	ALL	Low	Schools are unit of allocation to intervention or control group; measures taken to avoid cross‐over effects
		High	Classes, or individuals within schools are the unit of allocation to experimental or control group; no measures put in place to avoid cross‐over
Selective outcome reporting	ALL	Low	Outcomes proposed are outcomes that are reported
		High	Outcomes proposed are not the outcomes that are reported

Abbreviations: AC, age cohort design; BA/EC, quasi‐experimental design with before and after measures of bullying; RCT, randomized controlled trial.

This procedure was carried out by the first author in consultation with the second and third authors.^4^ There were a number of studies from the previous Campbell Collaboration report (i.e., Farrington & Ttofi, 2009) for which full texts were unavailable and thus, were excluded from several of the moderator analyses.

### 5.1 Descriptive

Various pieces of descriptive information were extracted from each of the 100 evaluations included in the present report. Information specific to the evaluation, such as the location or the start/end date, were recorded along with detailed information concerning the sample.

The total sample size and also the *n* of the relevant experimental and control groups were recorded. Age was extracted in two ways. First, where studies reported the mean age, or the age range (i.e., 8–10 years old) of participants this was recorded. Second, some studies did not report the age in years of participants, but we were able to record the school grade of included samples (i.e., Grades 4–6). Where reported, the % of females and males included in the sample was extracted.

We also coded descriptive information about the publication of the evaluation. Specifically, the type of publication and the publication year was recorded. The former represents a categorical moderator reflected whether or not the evaluation was published via the following channels, in order of hypothesized negative correlation with bias: (1) peer‐reviewed journal article; (2) chapter in an edited book/book; (3) governmental report or similar; (4) correspondence; and (5) unpublished masters or doctoral theses.

Correspondence was included to reflect data obtained from multiple evaluations of the Olweus Bullying Prevention Program (OBPP) sent to the second (M. M. T.) and third (D. P. F.) authors in preparation of their earlier Campbell review. Where evaluation data had been published in multiple formats, we favored the category associated with the least potential bias. For example, Domino (2011) reported the results of an evaluation of Take the LEAD program in a doctoral dissertation, but later published these results in a peer‐reviewed journal (i.e., Domino, 2013). In this scenario, the included study was coded as “article.”

### 5.2 Design

Included studies were further categorized according to several aspects of the research design used. We coded information regarding both the measures (i.e., instruments to measure bullying behaviors) and research design.

In relation to measurements of bullying, we recorded the timeframe (i.e., past 3 months or “ever”) in which participants were asked to report on experiences of bullying, the type of report used (i.e., self‐, peer‐, or teacher‐report), and data collection points (i.e., baseline, postintervention, 3‐month follow‐up, etc.). We also noted whether the measure was a continuous scale or a global item and whether bullying perpetration, victimization, or both, outcomes were measured.

As for the research design, we recorded information regarding the unit of allocation (or unit of randomization for RCTs; see below), the number of “clusters” included, whether groups were matched at baseline, and the number of experimental or control groups. For example, Elledge et al. (2010) included multiple control groups: matched controls and nonmatched controls.

Information about the evaluation methodology was also extracted from primary reports. The types of evaluation methodologies included in the present report are now described in further detail.

#### 5.2.1 Evaluation methodology

In order to optimize the comparability of effect sizes, primary studies included in a meta‐analysis should use the same, or at least conceptually similar, research designs (Wilson, 2010). Following Farrington and Ttofi's (2009) criteria, we searched for evaluations using any of the following four research designs:

(1) Randomized controlled trials (RCTs);
(2) Before‐after/quasi‐experimental‐control designs (BA/EC);
(3) Other quasi‐experimental designs; and
(4) Age cohort designs.

Each of these methodologies varied on four key elements: as randomization of participants (or clusters of participants); use of experimental and control groups; and administration of quantitative bullying measures before and after intervention.

For example, all studies coded as RCT had to include random assignment to experimental conditions (i.e., intervention and control groups) but did not have to use before and after measures of bullying outcomes. RCTs are considered to be the “gold standard” of experimental evaluations (Weisburd et al., 2001). Random assignment of a large number of units is used as a way in which evaluators can also randomize possible confounding variables between groups. As a result, we can infer that any observed differences result from the experimental manipulation (Farrington, 1983). The assumption is that randomization ensures that both observed and unobserved variables that may impact the results of an evaluation are also randomly distributed between groups. However, problems may arise if the unit‐of‐allocation, the unit‐of‐randomization, and the unit‐of‐analysis do not align.

Before‐after/quasi‐experimental‐control (BA/EC) designs, are conceptually similar to RCTs, but they do not involve random assignment to experimental conditions. Instead, participants or clusters of participants may be assigned to the intervention or control group on a self‐selected basis (e.g., Menesini et al., 2012), for convenience (e.g., Sapouna et al., 2010), or based on a greater need for intervention (e.g., Losey, 2009). Thus, BA/EC designs may be subject to selection biases (Farrington & Petrosino, 2001) that may reduce the validity of the results. These can be controlled if outcomes are measured before and after the intervention. Studies coded as BA/EC in the present report all used experimental and control groups but did not randomly assign participants to conditions. They also had to measure bullying outcomes before and after implementation of the intervention.

In contrast, studies categorized in the current review as using “other quasi‐experimental” designs utilized experimental and control conditions, without random assignment, but did not measure bullying behaviors before the intervention. Bullying outcomes were only measured after the implementation of an intervention in these studies. Therefore, selection bias is may be a threat to the internal validity of the results in such designs, which could have possibly attributed to pre‐existing differences between the groups (Farrington, 2003). For this reason, a decision was made to omit these designs from this updated meta‐analysis. Thus, relevant evaluations identified in the earlier Campbell Review and any new evaluations (since 2009) using this methodological design were excluded from the new meta‐analyses (see later).

In an age cohort design, students of a particular age *X* are initially assessed in the 1st year and serve as the control group for the evaluation of an intervention. Then, all students receive the intervention, and different students of the same age *X* (in the same school, in the 2nd year) serve as the experimental group (see Kärnä et al., 2013). This design, which is largely used in evaluations of the OBPP, deals with some selection effects, since it ensures that experimental and control children are matched on age and school, and it deals with some threats to internal validity (e.g., ageing and maturation). However, this design may be influenced by period and testing effects, and the experimental and control groups may differ on other uncontrolled variables.

Studies employing RCTs, BA/EC, and age cohort designs were included in the present systematic and meta‐analytic review. Because of the potential threat to internal validity, we excluded studies (*n* = 9) in the other quasi‐experimental design category because they are poorly controlled and vulnerable to selection effects. Additionally, the four studies included in the earlier review that used an “other quasi‐experimental” design were excluded from the present systematic review.

### 5.3 Program

Using a socio‐ecological systems theory framework (Bronfenbrenner, 1979) and the previous meta‐analysis (i.e., Farrington & Ttofi, 2009) as guidelines, information about the specific intervention program was recorded. General details about the intervention, such as the name of the program (where relevant) and the aim of the intervention (e.g., Silva et al., 2016) were noted along with more detailed information about the antibullying programs.

Intervention components at multiple levels of the socio‐ecological model (i.e., individual, peer, parent, and teacher, etc.) were recorded, such as work with peers, parental involvement, teacher training and whole‐school‐approach. Therefore, a brief description of each antibullying program based on this information is provided in Table 6.

**Table 6:** Systematic review results

Project	Antibullying program: key features	Participants	Research design
Randomized controlled trials (*n* = 45)			
Berry and Hunt (2009); Australia	*The Confident Kids Program*: CBT for anxiety management; target factors such as: self‐esteem, coping strategies, social skills, emotional regulation and internalizing behaviors. Eight weekly sessions lead by clinical psychologists	46 adolescent males (mean age = 13.04) who scored at least 1 *SD* higher than mean on a pre‐test anxiety measure and reported being bullied in the past month	Participants were assigned to groups based on their grade, and then these groups were randomly assigned to either intervention or waitlist control condition. Child‐ and parent‐report measures completed before, after, and at 3‐month follow up
Bonell et al. (2015); UK	*INCLUSIVE*: Whole‐school restorative antibullying program; action group of staff and students; needs assessment at baseline informed schools' intervention implementation. Core components: staff training in restorative practices and student social‐emotional skills curriculum	1017 year 8 students aged 12–13 years old in English secondary schools	Matched pairs of schools were randomly assigned to either the intervention (4 schools) or the control (4 schools) condition. Pre‐ and postmeasures of bullying were administered to all participants. Bullying perpetration measured by the self‐report AAYP violence scale and bullying victimization measured by the self‐report Gatehouse Bullying Scale
Brown et al. (2011); United States	*Steps to Respect*: Whole‐school program to reduce bullying by increasing staff efficacy, creating positive school climate, and increasing students' social and emotional skills. Classroom curriculum of 10 lessons implemented by trained teachers; individual bullies and victims received targeted intervention	4735 staff (*n* = 1307) and students (*n* = 2940) from public elementary schools. 128 staff members were teachers. 49% of students were male and 52% identified as white. The mean age of students was 8.9 years	34 matched school pairs where one of each pair was randomly assigned to the intervention condition, and the other to a waitlist control condition. Teacher‐report and self‐report measures completed before and after intervention
Chaux et al. (2016); Germany	*Media Heroes*: Cyberbullying prevention program; targets empathy, awareness and knowledge about bullying and cyberbullying; provides bystanders with effective intervention and prevention strategies	1075 students aged 11–17 (mean = 13.36) from five schools in Germany	Schools randomly assigned classrooms to one of three conditions: control; long‐version; or short‐version. Self‐report measures of bullying perpetration and bullying victimization were administered before and after the intervention
Cissner and Ayoub (2014); United States	*Fourth R: Strategies for Healthy Youth Relationships*: Dating violence prevention program; trained teachers implement 21‐lesson curriculum targeting: personal safety, healthy growth and sexuality, and substance use/abuse	517 7th grade students from 10 middle schools	Students from the 10 schools were randomly assigned to either the experimental or control condition, and all completed self‐report bullying measures (secondary outcome) at baseline, post intervention and 1‐year follow up
Connolly et al. (2015); Canada	*Youth led program*: High school students are trained to implement this school violence prevention program with middle school children; youth leaders were trained by mental health professionals; targeted students' knowledge and attitudes of peer aggression and victimization	509 7th and 8th grade students from Canadian middle schools, mean age = 12.37 and 51.4% were female	Four schools were randomly assigned to either intervention or usual practice control condition. All participants completed self‐report bullying measures (from the Safe School Survey) pre‐ and post‐intervention
Cross et al. (2011, 2004); Australia	*Friendly Schools Project*: Educational techniques based on Social Cognitive Theory; antibullying work implemented at whole‐school and community level, and also with students and their families; trained teachers implemented nine structured lessons	1968 4th grade students from schools in Perth. 51.1% of the intervention condition were female and had a mean age of 8.57 years. 48.3% of students in the control condition were female, and they had a mean age of 8.55 years	29 schools were randomly assigned to either intervention or standard curriculum control condition. Self‐report measures (OBVQ) of bullying perpetration and victimization was collected at 4 time‐points from all participants over the course of the 3‐year trial
Domino (2011, 2013); United States	*Take the LEAD*: Based on Social‐emotional learning and Positive Youth Development theories. Sixteen weekly lessons covered issues such as: self‐ and social‐awareness; self‐management; relationship skills; decision making; problem solving and leadership	323 7th grade suburban middle school students, with a mean age of 12.2 years and 93% were Caucasian	32 classrooms were randomly assigned to intervention or waitlist control group, and all participants completed self‐report bullying measures pre‐ and posttest
Espelage et al. (2013, 2015); United States	*Second Step: Student Success Through Prevention*: Social‐emotional learning middle school program; Trained teachers implement curriculum in 15 weekly classes, covering issues such as: empathy; communication; bullying; emotion regulation; problem solving; and substance abuse prevention	3658 students from 36 schools in Illinois and Kansas. Mean age was 11 years at the first time‐point, 1961 students received the intervention (52.1% male), and 1697 acted as controls (52.35% male)	36 schools grouped into matched pairs, and schools then randomly assigned to either the intervention condition or a waitlist control condition using a random number table. All participants completed bullying measures at three time points: Wave 1 (pre‐test); Wave 2 (posttest; Espelage et al., 2013); and Wave 3 (after 2 years of intervention). Bullying perpetration and victimization were measured using the self‐report Illinois Bully and Victim Scales
Fekkes et al. (2016); the Netherlands	*Dutch Skills for Life*: Universal school‐based prevention program for adolescents; delivered by trained teachers; 25‐lesson curriculum over 2 years; target: awareness and coping with emotions and feelings; problem‐solving; emotional regulation; bullying; friendship; sexuality; and substance abuse; activities included DVDs, role plays and group discussions	1394 students in grades 7–9 from 26 schools; aged 13–16 years old	Schools were randomized to the experimental condition (13 schools) or the control group (13 schools). Self‐reports of bullying perpetration and victimization were collected before the intervention (T0), after 1 year of implementation (T1), and at the end of the 2nd year of implementation (T2)
Garaigordobil and Martínez‐Valderrey (2015); Spain	*Cyberprogram 2.0*: Cyberbullying intervention program, traditional bullying also included; 19 lessons aim to raise awareness, outline the consequences of, and develop coping strategies relating to bullying and cyberbullying. Participants are also taught to develop positive social and emotional skills	176 secondary school students, aged 13–15 years old and 56.3% female. 93 students were in the intervention condition, and 83 were in the control condition	Classrooms from 3 different schools were randomly assigned to either the control or intervention condition and participants from both conditions completed self‐report bullying measures pre‐ and postimplementation
*Gradinger et al. (2014); Austria	*ViSC*: Training program led by professionals to increase students' sense of responsibility and competency in conflict; 13 structured lessons; covered topics such as: impulsivity; reflecting on behavior; and acting in a socially responsible manner	2042 students from 18 secondary schools, and 103 Grade 5–7 classrooms. 47.6% were female	13 schools were randomly assigned to the intervention condition, and five schools agreed to participate in the control condition. Internet‐based self‐report measures of traditional and cyber‐bullying were administered to all participants pre‐ and postimplementation
Holen et al. (2013); Norway	*Zippy's Friends*: Whole‐school program designed to increase coping strategies in order to reduce psychological problems; 24 weekly lessons given by trained teachers; curriculum based around concept of a character “Zippy” and his friends as they encounter several relationship problems	1483 2nd grade primary school children from 35 schools. 49.3% were female, and the mean age was 7.3 years	Schools were placed in matched pairs and randomly assigned to either the intervention or “business as usual” control condition. Teacher‐reported bullying measured by the Class Climate Survey at pre‐ and postintervention
Jenson et al. (2013, 2010); United States	*Youth Matters*: School violence program to increase school and peer norms against antisocial behaviors, such as, bullying; 10 modules that aimed to raise awareness, empathy about bullying and social skills	876 6th grade students from public elementary schools. Mean age was 9.82 years old, and 52% were female	Matched school pairs randomly assigned to intervention and control condition. Self‐report measures (OBVQ) administered at 2 time‐points: pretest (baseline) and posttest (12 month follow up)
Ju et al. (2009); China	*Chinese antibullying intervention program*: Action research framework; teachers designed and implemented a 5‐week intervention for the whole‐class, but also specifically for bullies and victims	354 3rd and 5th grade Chinese primary school children from one school. Two classrooms of each grade participated in evaluation	Two classrooms were randomly assigned to the intervention condition (one 3rd grade and one 5th grade) and the other two classrooms acted as controls (1 3rd grade and 1 5th grade). Chinese version of the self‐report OBVQ employed pre‐ and postimplementation
Kaljee et al. (2017); Zambia	*Teachers Diploma Program*: Situated supported distance learning program for educators; monthly community of practice meetings to review program content; target the interaction between psychological and social aspects of participants' lives; focus on self‐care, support skills, safe school environment, and positive inter‐school relationships	325 teachers and 1378 students from 20 experimental and 20 control schools. Mean age of students in 3rd and 4th grade was 10.9 years old and 55.8% were female	Waitlist randomized controlled design; students in classes in experimental schools randomly selected; students in classes in control schools randomly selected; both teacher‐report and self‐report measures administered before and after implementation
Kärnä et al. (2011b); Grades 4–6; Finland	*KiVa*: Whole‐school program that also targeted individual cases of bullying within a school; structured curriculum involving class and parent‐involved activities; antibullying computer program for students; training for teachers on classroom and bullying hotspot supervision/management	8237 students from grades 4–6 from 275 schools, 429 classrooms, aged 9–11 years old	78 schools were randomly assigned to intervention or control condition. All participants completed self‐ (OBVQ) and peer‐report (Participant Role Questionnaire) measures of bullying perpetration and victimization at baseline, mid intervention, postintervention
Kärnä et al. (2013); Grades 1–3; Finland	*KiVa*: see Kärnä et al. (2011b)	6927 students from grades 1–3 in 74 schools and 397 classrooms	74 schools were randomly assigned to intervention or control condition. All participants completed self‐ (OBVQ) and peer‐report (Participant Role Questionnaire) measures of bullying perpetration and victimization at baseline, mid intervention, postintervention
Kärnä et al. (2013); Grades 7–9; Finland	*KiVa*: see Kärnä et al. (2011b)	16,503 students from grades 7–9 in 73 schools and 1000 classrooms	73 schools were randomly assigned to intervention or control condition. All participants completed self‐ (OBVQ) and peer‐report (Participant Role Questionnaire) measures of bullying perpetration and victimization at baseline, mid intervention, postintervention
Knowler and Frederikson (2013); UK	*Emotional Literacy intervention*: 12‐week program led by trained professional; targeted students' emotional literacy skills; main concepts included: self‐awareness; self‐regulation; empathy; and social skills	50 primary school children, aged 8–9 identified as being involved in bullying behaviors using a peer nomination measure (Guess who measure)	Children assigned to intervention (*n* = 22; 18 male and 4 female) or waitlist control condition (*n* = 23; 21 male and 2 female). Guess‐Who peer nomination measure of bullying perpetration employed to all participants pre‐ and postintervention
Krueger (2010); United States	*School Bus antibullying intervention*: Intervention materials adopted from “Take a Stand, Lend a Hand, Stop Bullying Now!” online tools; DVD clips about bullying were shown to experimental students each day at the end of school	47 elementary school students that were assigned to one of two possible school buses	Randomly assigned students to either Bus A, who received the intervention, or Bus B, who were the control group. Data collected from all students prior to the intervention, and 5 days after
*Kyriakides, Creemers, Muijs et al. (2014); Multiple	*DASI*: Whole‐school antibullying European initiative; Targets school‐level factors, such as: school teaching policy, learning environment, and school evaluation. Cooperative committees formed of students, parents and teachers to tailor intervention curriculum to schools' needs	2948 participants from 15 schools in 5 different countries (Belgium, Cyprus, England, Greece, and Holland)	Schools were randomly assigned to either intervention (*n* = 1,456) or control (*n* = 1,492) groups, and all students completed the self‐report (OBVQ) measures of bullying behaviors before and after the intervention
*Kyriakides, Creemers, Papastylianou et al., 2014); Cyprus and Greece	*DASI*: Whole‐school antibullying European initiative; Targets school‐level factors, such as: school teaching policy, learning environment, and school evaluation. Cooperative committees formed of students, parents and teachers to tailor intervention curriculum to schools' needs	1345 Cypriote (*n* = 787) and Greek (*n* = 558) 6th grade students.	Randomly assigned schools to intervention or control condition, and all participants completed bullying measures (OBVQ) before and after implementation
Lewis et al. (2013); Li et al. (2011); United States	*The Positive Action program*: School well‐being program; Targets distal (school climate and teacher classroom management) and proximal (students' thoughts and feelings) factors to improve a range of health and behavioral outcomes	624 grade‐3 students were followed over 6‐year period	Matched school pairs randomly assigned to intervention or control group, in a longitudinal design with 8 waves of data collection. Self‐reported bullying‐related aggression measures employed at each time‐point
*Lishak (2011); United States	*Social Norms Project*: 12‐week program based on Social Norms Theory; student survey collected data on perceptions of bullying within the school; results relayed to participants through school‐wide assemblies and posters; specific interventions implemented	121 Grade 6–8 students at one public middle school. 85% identified as White/Caucasian. 28 students were allocated to the intervention condition, and 93 students acted as controls	Participants completed a self‐report web‐based questionnaire about several bullying‐related issues and both baseline and postintervention. Disciplinary referral logs were also utilized
*Low and Van Ryzin (2014); United States	*Steps to Respect*: Whole‐school program to reduce bullying by increasing staff efficacy, creating positive school climate, and increasing students' social and emotional skills. Classroom curriculum of 10 lessons implemented by trained teachers; individual bullies and victims received targeted intervention	2940 elementary school students aged 7–11 years old. 50.4% were male and 52.5% identified as being white	Randomly allocated matched school pairs to either intervention or waitlist control groups, and all participants completed pre‐ and postmeasures over the course of 1 year
McLaughlin (2009); United States	*CBT & CBT + media*: Standardized cognitive behavioral therapy (CBT) and an antibullying DVD. CBT was delivered in classrooms by a trained professional, and targeted bullying and aggression issues over 4 weekly lessons following a strict outline	68 6th grade students from 6 classrooms in 3 different schools. Mean age was 11.35 years old and 58.5% were female	Classrooms were randomly assigned to one of three conditions: (1) CBT only (*n* = 28); (2) CBT plus media, that is, the bullying DVD (*n* = 25); and (3) control group (*n* = 15). All participants completed self‐report measures of bullying perpetration and victimization (OBVQ) pre‐ and posttest
Nocentini and Menesini (2016); Italy	*KiVa*: Whole‐school program that also targeted individual cases of bullying within a school; structured curriculum involving class and parent‐involved activities; antibullying computer program for students; training for teachers on classroom and bullying hotspot supervision/management	2042 students from 13 Italian schools participated. 1039 students from 51 classes in seven schools participated in the intervention, and 1003 students from 46 classes in 6 schools participated as controls	Seven schools were randomly allocated to intervention condition, and 6 schools were randomly allocated to control condition. The Florence Bullying‐Victimization Scales self‐report measure of bullying perpetration and victimization were employed pre‐ and postintervention
Ostrov et al. (2016); United States	*Early Childhood Friendship Project*: Classroom‐based early childhood intervention; aims to reduce physical and relational aggression; target social‐psychological adjustment problems during development; include components on social modeling, problem‐solving and conflict resolution, modifying reinforcement contingencies, and social and emotional skills training	141 participants from six schools accredited for “Education of Young Children.” 47.5% were female (*n* = 67) and the mean age was 45.53 months old (approximately 3.79 years)	Six classrooms were randomly allocated to the intervention condition (*n* = 80) and six classrooms were randomly allocated to the control condition (*n* = 61). Bullying was measured using teacher‐ and observer‐report scale, the PBSM (Preschool Bullying Subscales Measure; Ostrov & Kamper, 2012)
Polanin (2015); United States	*Second Step*: Social‐emotional learning middle school program; trained teachers implement curriculum in 15 weekly classes, covering issues including bullying	55 students in the 5th grade at one middle school. Participants were aged 10 to 11, and 58% identified as Caucasian	Half of one of two classrooms were randomly assigned to the intervention condition or control. Self‐reported bullying perpetration and victimization were measured at 5 time‐points
*Şahin (2012); Turkey	*Empathy training program*: Program for children identified as bullies; 11 lessons following a curriculum that incorporated cognitive therapy techniques to increase students' empathetic skills and reduce bullying behaviors	38 students identified as bullies at baseline	Students were randomly assigned to one of four groups, and then two of the groups were randomly assigned to the intervention condition and the other two groups acted as a control group. Pre‐ and postmeasures were administered to all participants
Stallard et al. (2013); UK	*The Resourceful Adolescent Program*: Classroom‐based depression CBT program; 9 lessons outlined in a curriculum manual; core components include: psychoeducation; helpful thinking; personal strengths; problem solving; and support networks	1064 year 8–11 students in UK secondary schools identified at baseline as being “high risk” for depression. Participants were aged 12–16 years old	Year groups were randomly allocated to one of three possible experimental groups: (1) CBT intervention group; (2) Attention control group 1; and (2) control group 2. OBVQ administered at 3 time‐points (baseline, 6‐ and 12‐month follow ups) to assess change in bullying behaviors
Topper (2011); Study 1; United States	*Preventure*: Personality‐targeted CBT for high risk students in each of the four domains: hopelessness; anxiety‐sensitivity; sensation seeking; and impulsivity. Workshops were implemented by a trained professional	292 secondary school students from 9 different schools. Mean age was 14 years old, and 67% were female	Participants were randomly assigned to either intervention (*n* = 167) or control (*n* = 125) groups. Self‐report bullying measures (OBVQ) were administered at 4 time‐points: baseline and 6‐, 12‐, and 18‐month follow ups
Topper (2011); Study 2; United States	*Adventure*: extension of Preventure: Intervention followed a similar procedure to the preventure study, but CBT lessons were implemented by trained teachers	1089 secondary school students in years 9–11, from 18 different schools. 55.1% of participants were male, and the mean age was 13.71 years	Schools were randomly assigned to intervention (*n* = 625) or control (*n* = 464) condition, and all participants completed self‐report bullying (OBVQ) measurement instruments at baseline (preintervention) and 6‐, 12‐, and 18‐month follow up time‐points
Trip et al. (2015); Romania	*REBE and ViSC*: Dual components of Rational Emotive Behavioral Education and the ViSC social competence program; targets social‐emotional factors related to bullying and aggression	970 6th grade Romanian students from 11 different schools. Mean age was 11.82 years old, and 53% of participants identified as being male	Schools were randomly assigned to one of three potential conditions according to the order in which they were exposed to the intervention programs: (1) REBE then ViSC group (n = 385); (2) ViSC then REBE group (*n* = 270); and (3) control group (*n* = 315) who were not exposed to either program. Self‐reports of ever being bullied/ever bullied collected pre, during and post intervention
Tsiantis et al. (2013); Greece	*Greek antibullying program (2)*: School‐based program implemented by trained teachers and accompanying program manual; ongoing support from mental health professionals; 11 weekly workshops (90 min each); classroom activities included discussion groups, formation of class antibullying rules. Parent information sessions were also held	666 4th to 6th grade students from 20 elementary schools	Schools were matched based on prevalence levels of bullying and victimization. All participants completed the Greek version of the OBVQ (self‐report) pre‐ and postimplementation
Waasdorp et al. (2012); United States	*School‐wide Positive Behavioral Interventions and Supports*: Universal behavioral intervention program targeting school‐level factors; focuses on schools' discipline and behavioral management strategies to reduce bullying; bullying “hot spots” targeted for increased teacher supervision, and antibullying materials spread around the school	12,334 elementary school students from 37 U.S. public schools. 52.9% of participants were male and 46.1% identified as Caucasian	Schools randomly assigned to intervention or waitlist control condition, and teacher‐report (Teacher Observation of Classroom Adaptation‐Checklist) of bullying perpetration employed at pre‐ and postintervention
Wölfer and Scheithauer (2014); Germany	*fairplayer.manual*: 15‐week curriculum classroom‐based antibullying program delivered by either trained teachers or professionals. Aim to reduce bullying by increasing students' social and moral competencies. Lessons target: raising awareness, changing attitudes and encouraging bystander intervention	328 students in 7th to 9th grades from 2 German secondary schools. 51% were female and the mean age was 13.7 years old	Three class groups from each school were randomly selected and assigned to the intervention group. The remaining participants acted as waitlist control group. Pre‐ and post‐self‐report measures of bullying perpetration and victimization (OBVQ) were implemented 4 months apart
*Wurf (2012); Hong Kong	*Shared Concern*: Whole‐school antibullying program based on the Pikas method of Shared Concern; intervention involves teacher‐led restorative and nonpunitive conflict resolution between bullies and victims	549 year 7 students across 21 classes in 4 international secondary schools in Hong Kong	Schools were randomly assigned to one of four possible conditions: (1) whole‐school intervention; (2) standard curriculum and shared concern intervention in year 7; (3) shared concern only in year 7; and (4) control group. OBVQ administered pre‐ and posttest
*Yabko (2013); United States	*Ninja Mind Training program*: Web‐based intervention; CBT and mindfulness based; 4 weekly 35 min sessions; bullying‐related vignettes and materials and mindfulness exercises; reflection activities	32 6th to 8th grade students that were identified by teachers as victims of bullying, or who had not participated in school's existing program. 68.8% of students were male	Students were randomly assigned to the intervention or treatment‐as‐usual control condition and all completed bullying measures before and after the intervention
Yanagida et al. (2019); Austria	*ViSC*: Training program led by professionals to increase students'' sense of responsibility and competency in conflict; 13 structured lessons; covered topics such as: impulsivity; reflecting on behavior; and acting in a socially responsible manner	2042 secondary school students from 103 5th to 7th grade classrooms in 26 schools in Vienna. 1377 were in the intervention group and 665 were in the control group. 47.6% were female and the mean age was 11.7 years old	Thirteen schools were randomly assigned to the intervention group and 13 schools were randomly assigned to the control group. All participants completed outcome measures for bullying perpetration and victimization pre‐ and postimplementation
*Before‐after, experimental‐control designs (n = 27)*			
Battey (2009); United States	*The Bully Prevention Challenge Course Curriculum*: Activity‐based antibullying program implemented by Physical Education/Health teachers; intervention includes warm‐up activities, group discussions and raising awareness about bullying	249 7th grade students from two public middle schools	Intervention (*n* = 120) and control (*n* = 129) students all completed bullying measures pre‐ and postimplementation
Bull et al. (2009); Germany	*fairplayer.manual*: Weekly curriculum classroom‐based antibullying program delivered by either trained teachers or professionals. Aim to reduce bullying by increasing students' social and moral competencies. Lessons target: raising awareness, changing attitudes and encouraging bystander intervention	119 7th to 9th grade students from one German secondary school. 64 were female and the mean age was 15.13 years old	Three experimental groups were employed according to the duration of intervention they received: (1) received 10 weeks of the intervention over the course of 15–17 weeks; (2) received 10 weeks of intervention over 12 months; and (3) control group that were not exposed to intervention. All participants completed bullying measures, pre, post (+4 months) intervention and at a 12 month follow up
Elledge et al. (2010); United States	*Lunch Buddy mentoring program*: Victims of bullying are paired with a trained college mentor; mentors and mentees meet twice a week, over the course of 5/6 months; mentors sit with mentees during lunchtimes and provide social and emotional support	36 students from 4 primary schools, grades 4 and 5, whom teacher and peer report indices identified as being victims of bullying. Mean age = 10.36 years old	Employed three experimental groups: (1) intervention group (*n* = 12); (2) “Same” control group who were from the same school as the experimental group (*n* = 12); and (3) “Different” control group who were from a different school (*n* = 12). All participants completed bullying measurement instruments pre‐ and postimplementation
Finn (2009); United States	*Olweus Bullying Prevention program*: Whole‐school approach; Individual‐, peer‐, classroom‐, teacher‐, and school‐level factors included	801 3rd to 5th grade students from 4 elementary schools	Assigned 2 schools to intervention condition (*n* = 437) and 2 schools to control condition (*n* = 383). All participants completed the OBVQ pre‐ and postimplementation
*Harpin (2011); United States	*Lead Peace intervention*: Resiliency based program, aiming to provide students with the skills to prevent them from being bullied; curriculum targets factors at the environmental, personal and behavioral levels	218 students from 4 middle schools	Data was collected at four time‐points, baseline, and after each of the 3 years of implementation
Herrick (2012); UK	*Defeat Bullying*: Curriculum‐based antibullying program developed by the NSPCC; targets several key bullying‐related issues, such as, attitudes and feelings about bullying, diversity, safety and encouraging bystanders to prevent, or intervene in, bullying	69 year 5 students from 3 primary schools	Utilized a pre/post nonequivalent quasiexperimental design. School 1 received the intervention; School 2 received the intervention plus parental involvement; and School 3 acted as a waitlist control school
Joronen et al. (2011); Finland	*Drama program*: Based on drama and social cognitive theories; trained teachers implemented one drama session per month; themes included: bullying, friendship, loss of a friend, supporting a victim of bullying, tolerance and child abuse	190 Grade 4 and 5 students from 2 Finnish primary schools	Schools were purposively allocated to the intervention or control condition, and bullying was measured pre‐ and postimplementation of the intervention program
Losey (2009); United States	*OBPP*: Whole‐school program, also included individual‐, class‐, and community‐level factors; school conference held at beginning of program; detailed teacher handbook; parent/teacher meetings; class antibullying rules	699 high school students from 2 U.S. schools, 416 were female	Schools were allocated to intervention (*n* = 251 students) or control (*n* = 448 students) by the region's superintendent based on prevalence of bullying. All participants completed the Revised OBVQ pre‐ and posttest
Menard and Grotpeter (2014); Menard et al. (2008); United State	*Bully‐Proofing Your School*: Whole‐school program; Individual support also provided for bullies and victims; restorative nonpunitive disciplinary policies; classroom curriculum implemented by teachers; parent information	3,497 3rd to 5th grade students from 6 elementary schools, 52.1% were female	Assigned schools to either intervention or control conditions in a nonequivalent groups design. All participants completed bullying measures pre‐ and posttest over 5‐year period
Menesini et al. (2012; Study 1); Italy	*Noncadiamointrappola (Let's Not Fall Into a Trap); NoTrap!*: Web‐based peer‐led antibullying intervention; selected group of adolescents monitor an online antibullying forum; In‐class antibullying activities	386 secondary school students at 8 Tuscan schools, 20.3% were male, and the mean age was 16.29 years old. 9th to 13th grade students for intervention running from December 2009 to June 2010	Students were assigned to one of three potential groups: (1) control group, (2) intervention group, and (3) peer educators. Bullying measures were administered pre‐ and posttest (6 months apart)
Ortega‐Ruiz et al. (2012); Spain	*ConRed*: Cyberbullying prevention program; developed using evidence on effective antibullying intervention components; Involves several strategies: (1) proactive policies, procedures and practices; (2) school community key understandings and competencies; (3) protective school environment; (4) school‐family‐community partnerships	893 high school students, 595 were in the intervention group (45% female) and 298 in the control group (47.6% female). Students were aged 11–19, with a mean age of 13.8 years old	Researchers and teachers allocated classes of students to experimental or control groups; All participants completed the European Bullying Intervention Project Questionnaire (ECIPQ; Brighi et al., 2012) before and after implementation
Palladino et al. (2012); Menesini et al. (2012; Study 2); Italy	*NoTrap!*: Web‐based peer‐led antibullying intervention; selected group of adolescents monitor an online antibullying forum; in‐class antibullying activities	375 9th to 13th grade students at 4 Tuscan high schools for year December 2010 to June 2011	Students were assigned to one of three potential groups: (1) control group; (2) intervention group; and (3) peer educators. Bullying measures were administered pre‐ and posttest (6 months apart)
Palladino et al. (2016; Trial 1); Italy	*NoTrap!*: Web‐based peer‐led antibullying intervention; selected group of adolescents monitor an online antibullying forum; In‐class antibullying activities	622 9th grade students from 8 high schools in Tuscany during the school year 2011/2012. 22 classes in 5 high schools were allocated to the intervention condition (*n* = 451; mean age = 14.79; 57% male) and students from 9 classes in 3 high schools participated as controls (*n* = 171; mean age = 15.28; 69% male)	All participants completed the Florence Bullying‐Victimization scales at pre‐ and posttest. Scale measures the frequency of bullying perpetration and victimization experienced by respondents during the past 2 months
Palladino et al. (2016; Trial 2); Italy	*NoTrap!*: Web‐based peer‐led antibullying intervention; selected group of adolescents monitor an online antibullying forum; in‐class antibullying activities	461 9th grade students from 7 high schools in province of Lucca during the school year 2012/2013). 10 classes from 4 schools were assigned to the intervention condition (*n* = 234; mean age = 15.6; 28.6% male). Students from 10 classes in 3 schools acted as controls (*n* = 227; mean age = 15.57; 76.2% male)	All participants completed the Florence Bullying‐Victimization scales at pre‐ and posttest. Scale measures the frequency of bullying perpetration and victimization experienced by respondents during the past 2 months
*van der Ploeg et al. (2016); the Netherlands	*KiVa*: Whole‐school program that also targeted individual cases of bullying within a school; structured curriculum involving class and parent‐involved activities; antibullying computer program for students; training for teachers on classroom and bullying hotspot supervision/management; support group approach for victims of bullying	56 victims from 28 schools enrolled in the Dutch national implementation of the KiVa antibullying program. 30 were female and the mean age was 9.15 years old	Victims that received a support group were statistically matched to those that did not received a support group (*n* = 571). All participants completed bullying measures pre‐ and postimplementation
Pryce and Frederickson (2013); UK	*Anti‐Bullying Pledge Scheme*: Local antibullying initiatives implemented in UK schools; each school assigned an intervention facilitator; whole‐school intervention is tailored to each schools' specific needs	338 students from years 4, 5, and 6 classrooms in 4 UK primary schools. 160 were female and participants were aged 8–11 years old	Two schools were assigned to the intervention condition and two schools acted as a treatment as usual control group. Pre‐ and postdata collection was conducted with all participants
Rawana et al. (2011); Canada	*Strengths in Motion*: Strength‐based whole‐school antibullying intervention; enhancing individuals' strengths; designated intervention classroom within experimental school; room used as: (1) good start centre; (2) cool down and prevention; (3) good choices room; and the site of an ambassador's club	103 4th–8th grade students from 2 elementary schools; 50 were allocated to experimental condition (mean age = 11.04; 58% female) and 53 were placed in control condition (mean age = 11.53; 45.5% female)	All participants completed the self‐report Safe School Survey, which includes a measure of students' experiences of bullying perpetration and victimization, at baseline, post‐implementation (3 months later), and 8‐month follow‐up. Schools were allocated to experimental or control
Sapouna et al. (2010); UK and Germany	*FearNot!*: Immersive learning intervention; virtual‐learning; 30 min sessions for 3 weeks; bullying scenarios acted out by virtual reality characters; participants required to select appropriate reactions or responses of character	942 primary school students from the UK (*n* = 520) and Germany (*n* = 422). The mean age of UK participants was 9.36 years and in German schools the mean age was 8.34 years	Schools with up‐to‐date computer facilities required to administer the intervention were assigned to the intervention condition, while the other schools acted as a control group. Pre‐ and postintervention measures were employed with all participants
Silva et al. (2016); Brazil	*Skill‐based intervention*: Behavioral cognitive intervention based on social skills; 8 weekly classes for 50 mins led by clinical psychologists; groups were mixed by gender and bullying‐involvement status; targeted: civility, making friends, empathy, self‐control, emotional expressiveness, assertiveness, interpersonal problem‐solving; activities included role‐play, dramatization, positive reinforcement, modeling, feedback, videos and homework assignments	188 6th grade students from six schools. Mean age in intervention group was 11.28 years and the mean age in the control group was 11.21 years	18 classrooms were randomly assigned to intervention (*n* = 9 classes) and comparison (*n* = 9 classes) groups. All participants completed a self‐report measure of aggression and peer victimization before and after intervention
Sismani et al. (2014); Cyprus	*Daphne III*: International antibullying initiative; educate 5th and 6th grade primary school children about bullying and its many forms; 11 workshops following a structured curriculum manual	188 5th and 6th grade students from Cypriote primary schools	All students completed the OBVQ pre‐ and postintervention. Students were allocated to either the intervention group or control group
Solomontos‐Kountouri et al. (2016); Cyprus	*ViSC*: Training program led by professionals to increase students' sense of responsibility and competency in conflict; 13 structured lessons; covered topics such as: impulsivity; reflecting on behavior; and acting in a socially responsible manner	1652 students from 82 classes in 6 schools. Mean age was 12.6 years old and 48.9% of the sample were female	30 classes (*n* = 602 students) of 7th grade and 8th grade students were allocated to the intervention condition, and 52 classes (*n* = 1,050 students) were allocated to the control condition. Self‐report measures of bullying perpetration and bullying victimization were collected at three time‐points, before and after implementation, and follow‐up
Sutherland (2010); Canada	*Beyond the Hurt*: Peer‐led antibullying program; high‐school program involving four key components: (1) training of peer facilitators, (2) in‐class presentations, (3) teacher workshops, (4) and online training materials for teachers and parents	621 high school students in Canada. 47% were male and 93% reported being Caucasian	Schools were allocated to the intervention or waitlist control condition and bullying measures were conducted pre‐ and postimplementation in both groups
Toner (2010); United States	*Bully‐Proofing your School*: Whole‐school program; individual support also provided for bullies and victims; restorative nonpunitive disciplinary policies; classroom curriculum implemented by teachers; parent information	149 6th grade students from 2 suburban public elementary schools. School S—implemented BPYS (*n* = 58) and School U—control (*n* = 91)	Participants in experimental and control schools completed a self‐report measure of direct and indirect bullying perpetration and victimization, pre‐ and postimplementation
		63.8% of participants were female and 62.4% were White	
Williams et al. (2015); United States	*Start Strong*: School‐based teen dating‐violence prevention program; bullying included as secondary violence outcome.	1517 students from 8 middle schools. Sample was ethnically diverse with 23% identifying as White; 28% African‐American; and 33% Latino	Matched school pairs were created with one school from each pair being allocated to the intervention condition. The remaining schools formed the control group. Data collected pre‐ and postintervention
Wong et al. (2011); Hong Kong	*Restorative Whole‐school Approach*: Whole‐school antibullying program based on restorative justice principles; whole‐school nonpunitive antibullying policy and ethos implemented; curriculum lessons target: empathy, assertiveness, coping, problem‐solving and conflict resolution	1480 high school students from 4 “middle band” (based on academic ratings) schools in Hong Kong. Students were aged 12–14 years old	Three experimental groups were utilized: (1) Intervention group; (2) partial intervention group; and (3) control group. All participants completed pre‐ and postmeasures of bullying
Yaakub et al. (2010); Malaysia	*OBPP*: Whole‐school program, also included individual‐, class‐, and community‐level factors; school conference held at beginning of program; detailed teacher handbook; parent/teacher meetings; class antibullying rules	3816 students from 6 secondary schools in Malaysia	Three schools were assigned to the intervention condition, and the remaining three acted as a control group. Participants from both groups completed bullying measures pre‐ and postintervention
*Age cohort designs*			
Busch et al. (2013); the Netherlands	*Utrecht Healthy Schools*: Whole‐school health program; implement a healthy‐school policy; ensure healthy food options, smoke‐ and alcohol‐free sites and appropriate sports facilities; parent workshops and take‐home tasks; involve public health services	336 4th grade students aged 15–16 years old	Fourth grade students before the 3‐year intervention were compared with fourth grade students after the implementation
*Frey et al. (2009)^a^; United States	*Steps to Respect*: Whole‐school program to reduce bullying by increasing staff efficacy, creating positive school climate, and increasing students' social and emotional skills. Classroom curriculum of 10 lessons implemented by trained teachers; Individual bullies and victims received targeted intervention	624 students in grades 3–5	Extension of Frey et al. (2005) which was an RCT design. Used these figures as independent estimate of effectiveness
Kärnä et al. (2011a); Finland	*KiVa*: Whole‐school program that also targeted individual cases of bullying within a school; structured curriculum involving class and parent‐involved activities; antibullying computer program for students; training for teachers on classroom and bullying hotspot supervision/management	Approximately 200,000 students in 888 Finnish schools. 156,634 and 156,629 students comprised the control groups for victimization and perpetration respectively. 141,103 and 141,099 students comprised the intervention groups for victimization and perpetration respectively	Cohort‐longitudinal design with adjacent cohorts. All participants completed the Revised Olweus Bully/Victim Questionnaire
Limber et al. (2017); United States	*Olweus Bullying Prevention Program*: School level (e.g., staff discussion groups; bullying prevention coordinating committee); classroom level (e.g., classroom rules); individual level (e.g., supervision of students); and community level components	70,998 students from 210 schools in grades 3–11	Extended age cohort design. All students completed the self‐report OBVQ measure of bullying perpetration and victimization
Olweus; New National Cohorts 1 to 6 Norway	*OBPP*: School level (e.g., staff discussion groups; Bullying Prevention Coordinating Committee); Classroom level (e.g., classroom rules); individual level (e.g., supervision of students); and community level components	Six cohorts from a national implementation of the OBPP	Extended selection cohorts design; testing began in October 2001, and subsequent measurements at half‐year intervals
Purugulla (2011); USA	*OBPP*: School level (e.g., staff discussion groups; Bullying Prevention Coordinating Committee); Classroom level (e.g., classroom rules); individual level (e.g., supervision of students); and community level components	785 7th grade (*n* = 399) and 8th grade (*n* = 386) students in year one of evaluation and 847 *7th grade* (*n* = 417) and 8th grade (*n* = 410) students from one middle school	Age cohort design, with year 1 students acting as control for experimental year 2 students. All participants completed OBVQ measure of bullying and bullying‐related discipline records were also obtained
Roland et al. (2010); Norway	*Zero Program*: Preventive program; emphasis on school staff to ensure a zero tolerance to bullying; discussion groups about bullying occur in classes; restorative conflict resolution meetings take place between victims, teachers, parents and then, perpetrators	20,446 students in years 2–7 from 146 Norwegian schools	Age equivalent design; surveys were administered in Spring 2001 and 2004

In addition to specific program elements included in interventions, we also coded for possible sources of bias in evaluations and intervention development. Conflict of interest (COI) has previously been reported to impact evaluation results of many interventions and is a growing area of interest (COI; Eisner & Humphreys, 2012) with studies identified as having higher COI associated with larger overall effect sizes. Eisner and Humphreys outline many other possible sources of COI, such as financial gain to the evaluator, but this information was difficult to obtain for antibullying programs. Thus, a simple indication of potential COI was utilized.

We primarily focused on the overlap between individuals included as author/coauthor on the evaluation study, is also included on previous evaluations of the same program (e.g., NoTrap!; Menesini et al., 2012; Palladino et al., 2012, 2016), or is in fact referenced as the developer of that particular program (e.g., Tsiantis et al., 2013). If no reference to a publication relating to the specific program was included, we concluded that the author had developed the program, and thus, the evaluation was deemed high risk.

Program specificity refers to whether the intervention program was specifically targeting bullying outcomes, or if many other outcomes were also included. Targeted programs are suggested to be more effective than generalized programs that aim to reduce many different behaviors in one intervention. Highly specific programs (i.e., those that only included bullying outcomes and very few others) were coded as “high.” Thus, programs that were less specific and included many other outcomes in addition to bullying measures were considered “low.” A third category was created (i.e., “medium”) to include studies that did multiple other outcomes in addition to bullying outcomes, but these additional variables were bullying‐related.

### 5.4 Outcomes

We also extracted several pieces of statistical information from primary studies that was required for the estimation of effect sizes. Statistics for bullying behaviors, for example, means and standard deviations or sample sizes and percentage of bullies and/or victims, were extracted for experimental and control groups at baseline and immediately postintervention timepoints.

We also coded bullying data for additional follow‐up timepoints where this information was reported by primary studies. Data was extracted and recorded separately for independent samples (i.e., female and male, Palladino et al., 2016; older and younger, Baldry & Farrington, 2001) and different measures. For example, data for both self‐ and peer‐report measures were extracted from Beery and Hunt (2009) and for different forms of bullying (e.g., Frey et al., 2005).

### 5.5 Risk of bias

As per the Campbell Collaboration reporting guidelines, a risk of bias index was created for the purpose of the present report. The EPOC tool was utilized to assess the risk category of each study on several items relating to the methodological quality of evaluations. Following earlier Campbell review (e.g., Valdebenito et al., 2018) this tool was also used for nonrandomized studies as other risk of bias measurement instruments were considered inappropriate for nonscientific or medical trials.

The following section describes the procedure for addressing risk of bias in the present meta‐analysis. Each primary evaluation was measured on the following items: (1) allocation sequence (AS); (2) Allocation concealment (AC); (3) Baseline equivalence on outcomes (BE); (4) Baseline equivalence on participant characteristics (BC); (5) Incomplete outcome data (ID); (6) Contamination protection (CP); and (7) Selective outcome reporting (SOR). The applicability of these categories for each of the methodological designs included in the present report is outlined in Table 5. Each study was categorized as being high, low, or unclear (if insufficient information was available) risk on each of these EPOC items.

## 6 INCLUDED INTERVENTIONS

In total, 67 different school‐based antibullying programs were evaluated by primary studies included in our updated meta‐analysis. Descriptions of each of these interventions is provided in the following section of this report. These narrative reviews of included antibullying programs are based on the best available information provided by the primary studies. Twenty‐one of the evaluated antibullying programs were included (only) in the previous meta‐analysis (Farrington & Ttofi, 2009). A number of popular school‐based antibullying programs (n = 7; i.e., Bully Proofing Your School [BPYS], Friendly Schools, KiVa, OBPP, Steps to Respect, ViSC, and Youth Matters) had been re‐evaluated or additional publications since 2009. Hence, the majority of programs evaluated in our updated meta‐analysis (*n* = 40) are new bullying prevention and intervention programs.

The following sections provides detailed summaries of each antibullying program included in our systematic review. Descriptions marked with an * were taken from the previous review (Farrington & Ttofi, 2009). To provide the reader with a detailed overview of existing antibullying programs studies subsequently excluded from the meta‐analysis are also included here.

### 6.1 *Antibullying intervention in Australian secondary schools

This antibullying intervention consisted of several activities that aimed to increase awareness and identification of bullying, to promote empathy for targets of bullying and to provide students with strategies to cope with bullying (Hunt, 2007, p. 22). The intervention was based on an educational antibullying program, which was delivered by teachers. There was no specific training for teachers. Information about bullying was provided at parent and teacher meetings. Teacher meetings were held in conjunction with regular staff meetings while parent meetings were held after hours. A summary of the information covered at parent meetings was also published in the school newsletter in an attempt to target the wider parent population. Finally, the program includes a 2‐h classroom‐based discussion of bullying (offered by teachers) using activities from an antibullying workbook written by Murphy and Lewers (2000).

### 6.2 Anti‐Bullying Pledge Scheme (ABPS)

The ABPS describes a number of local antibullying schemes implemented in UK schools as a result of government recommendations and guidance (Pryce & Frederickson, 2013). Schools adopted a declaration of commitment, and intervention components followed a theoretical framework guided by the Theory of Planned Behavior (Ajzen, 1991).

The ABPS is a universal prevention program, that aims to reduce the prevalence of bullying perpetration and victimization in schools and increase students' perceptions of safety and support within the school environment (Pryce & Frederickson, 2013). Participating schools were assigned a facilitator, referred to as a “pledge supporter,” and a detailed intervention manual. The manual outlined the stages involved in implementing the ABPS program. The stages are as follows:

- Initial meeting with school management and the pledge supporter
- Intervention planning meeting
- School representatives make a declaration of commitment to the intervention
- Staff, student, and parent surveys are circulated
- Results from the surveys were collated and used to tailor intervention components to the individual schools' needs
- Ongoing visits and support from the pledge supporter throughout implementation.

### 6.3 *Be‐prox program

The Be‐Prox program was specifically designed to tackle bullying and victimization among kindergarten students. According to Alsaker and Valkanover (2001, pp. 177–178), the somewhat higher adult‐children ratio, the interest of preschool teachers in socialization, the greater flexibility as to scheduling and teaching, and the admiration of many preschoolers for their teachers are ideal conditions for the implementation of preventive programs against bully/victim problems. The basic principle of Be‐Prox was to enhance preschool teachers' capacity to handle bully/victim problems (Alsaker, 2004, p. 291). The program engaged teachers in an intensive focused supervision for approximately 4 months. Central features of Be‐Prox were the emphasis on group discussions, mutual support and co‐operation between consultants and teachers and between teachers and parents (Alsaker, 2004, pp. 292–293).

The teacher training was provided in six steps (Alsaker, 2004; fig. 15.1, p. 292). Initially, teachers were given information about victimization (step 1) and the implications of this information was discussed (step 2). During the third step, specific implementation tasks were introduced and the teachers worked in groups in preparation for the practical implementation (step 4). After this preparation, teachers implemented specific preventive elements in the classroom (step 5) for a specific period of time. After that, teachers met and discussed their experiences of the implementation of the preventive measures (step 6).

In eight meetings over a 4‐month period, issues related to the prevention of bullying were addressed. The main purpose of the first meeting was sensitization. Teachers were asked to describe any possible bully/victim problems in their schools and were then given information about bullying and other types of aggressive behavior. They were also presented with the main principles of the program. The importance of contact between kindergarten teachers and children's parents was also emphasized and teachers were advised to consider the possibility of organizing a meeting with parents. In the second meeting, the importance of setting limits and rules to preschool children was discussed. Teachers were invited to elaborate some behavior codes in their classroom in collaboration with the children and to be ready to present them during the third meeting. Also, as a second homework task, teachers were asked to organize a parent meeting.

During the third meeting, teachers discussed their experiences of implementing classroom rules against bullying. The main focus of this meeting was the need for consistent teacher behavior, the difference between positive and negative sanctioning and the use of basic learning principles in the classroom. The main focus of the fourth session was on the role and responsibility of children who were not involved in bullying and of bystanders in the prevention of victimization. Teachers were asked to draw some kind of personality profiles of passive and aggressive victims and of bullies and to present them to the rest of the group. After this task, teachers were presented with research findings regarding the characteristics of children who were or were not involved in bullying. As a homework task for the next meeting, teachers were asked to systematically observe noninvolved children and to develop some means of involving them in the prevention of victimization.

During the fifth meeting, research‐based information about motor development and body awareness among preschool children was presented to teachers. A discussion between teachers and program researchers of children's self‐perceptions of strength, of peers' perceptions of strengths of victims of bullies, and other motor characteristics of children, aimed to yield important insights. The overall discussion and exchange of information among teachers aimed to promote teachers' understanding about how to change these perceptions within the classroom setting. Specific goals to be achieved within the classroom were clearly set, such as training in empathy and body awareness among children, participation and involvement of noninvolved children and talks with all the children about the situation in their kindergarten. During the sixth meeting, time was given to reflect on the goals formulated at the beginning of the prevention program. Teachers were also given time to discuss their experiences with implementing the goals of the fifth meeting within the classroom settings. The last two meetings followed a similar format, with time given for reflection on goals achieved, problems dealt with, and an overall evaluation of the program.

### 6.4 *Befriending intervention

Befriending intervention was an antibullying program that relied mainly on a peer support model. The overall aims of the program were: (a) to reduce bullying episodes through developing in bullies an awareness of their own and others' behavior; (b) to enhance children's capacity to offer support to the victims of bullying; (c) to enhance responsibility and involvement on the part of bystanders; and (d) to improve the quality of interpersonal relationships in the class group (Menesini et al., 2003, p. 1).

The antibullying intervention was offered in five steps (Menesini et al., 2003, p. 5). During the first phase, which targeted the class level (class intervention), several activities were offered aiming to increase children's awareness of prosocial and helping behaviors and to promote positive attitudes toward others. Through work at the class level, the school authorities sensitized and prepared the whole school population for the new service that the school unit was about to implement. In this way, another goal was achieved, namely developing values and attitudes toward “peer support activities” in the whole school population.

During the second phase of the program, the “peer supporters” were selected. Approximately three to four supporters were allocated in each classroom and were selected based on a combination of techniques, such as self‐ and peer‐nominations. These children were then trained in special full‐day sessions or in regular meetings during school time (phase three) so that they knew how to deal with other children and how to facilitate interactions among other children. Teachers and other professionals (psychologists and social workers) took part in these sessions as well. The overall aim of this phase of the antibullying program was to help peer supporters to enhance their listening and communication skills since they would be the mediators in the interactions among children.

During the fourth phase of the program, peer supporters worked in their classes with the assistance and close monitoring of their teachers. The teachers in each class organized “circle meetings” during which the needs of specific children involved in bullying (target children) were identified. Target children were contacted and, after their consent and cooperation, were offered help by the peer supporters. Peer supporters were not only assigned to specific tasks involving the target children but were also supervised by the teachers so that they were given constant feedback on their on‐going work in the class.

During the final phase of the Befriending Intervention, the leading group of peer supporters were involved in training other children in the class, so that more children could be involved in the program (in the transmission of training and passing on the roles).

### 6.5 *Behavioral program for bullying boys

This program targeted male youth, from a low socio‐economic area, predominately inhabited by individuals of color, involved in bullying. The program was based on the findings of an in‐depth needs assessment within three schools and targeted a specific number of male students aged sixteen who (based on the results of the questionnaire that had been administered) were “considered to be a serious threat to the harmonious functioning of everyday school life” (Meyer & Lesch, 2000, p. 59). The theoretical basis of the program could be found in the Social Interactional Model for the development of aggression (Meyer & Lesch, 2000, p. 61) and involved a behavioral approach for tackling the problem of bullying. The program was implemented by psychology students for ten nonconsecutive weeks, with 20‐h‐long sessions held twice weekly at the school, during school hours.

The components of the 17‐session behavioral program included homework tasks, modeling, self‐observation, role‐plays, and a token economy system for reinforcing positive behaviors. According to the program designers “the chief contingency for behavioral change was the token economy system, using Wonderland Games tokens, chocolates and cinema tickets as reward for non‐bullying behavior” (Meyer & Lesch, 2000, p. 62). Each participant was monitored by himself and by a “buddy” who was selected in each session prior to the monitoring. Each session included an opportunity for feedback on the students' progress in the week, a discussion of a relevant applied topic, role‐playing, games, and drawing. The program designers pointed out the limitations of the intervention strategy. As they indicate (Meyer & Lesch, 2000, p. 67) “the program was too short and structured to address the issues that were disclosed in sessions, as the severity of the nature of the aggression in the schools and vast social problems was seriously underestimated.”

### 6.6 Beyond the Hurt

Sutherland (2010) implemented the Beyond the Hurt program, a peer‐led school‐based bullying intervention and prevention program, developed by the Red Cross. Beyond the Hurt is a high school program and emphasizes education, prevention and intervention to reduce prevalence of bullying perpetration and victimization. Sutherland (2010, p. 84) describes the four key components of the intervention: (1) education and training of peer facilitators, (2) in‐class presentations given by peer facilitators, (3) teacher workshops, and (4) online training material for teachers and community members.

This peer‐led program trains and educates select peer facilitators, who become the implementers of the intervention program within participating schools. These students are guided by a teacher and Red Cross professional throughout training and implementation of class presentations highlighting several bullying‐related issues. The overarching aim of the Beyond the Hurt program is to create a positive school and class climate in which students are encouraged to develop and maintain healthy prosocial relationships, and bullying perpetration and victimization are not supported. The program aims to promote antibullying attitudes among participants and encourage empathy and prosocial support for victims of bullying.

### 6.7 *Bulli and Pupe

Bulli and Pupe was an intervention program concerned with bullying and family violence. The program, developed by Baldry (2001), was “directed towards the individual and peer group, and aimed to enhance awareness about violence and its negative effects” (Baldry & Farrington, 2004, p. 3). The intervention package consisted of three videos and a booklet divided into three parts; each video was linked to one part of the booklet. Each part of the booklet was meant to take the form of an interactive lesson where professionals, experienced in school and juvenile processes, discussed three issues according to the structure of the manual.

The first part of the booklet, entitled “Bullying among peers,” emphasized teen violence among peers. The booklet presented vignettes and graphics that reported research findings on bullying in an attempt to raise students' awareness of this issue. The corresponding video showed teenagers talking about bullying based on their own experiences and judgments. The second part of the booklet, entitled “Children witnessing domestic violence,” analyzed the effects of domestic violence on children and the repercussions for school achievement and peer relations. In the accompanying video, children in a shelter for battered women were presented, talking about their personal experiences and emotions. Finally, the third part of the booklet, entitled “Cycle of violence,” dealt with the long‐term effects of violence on adults who were victims of violence in their childhood. The corresponding video consisted of an interview conducted with a 19‐year old boy who had a violent father.

The program was in the first place delivered in 3 days by experts who, together with teachers, discussed about bullying, read the booklet and analyzed its content. The program was taken over by teachers who once a week created a facilitation group and allowed children to discuss any problems they encountered with their peers. The program was more effective with secondary students because it required its participants to have good interpersonal and cognitive skills (Baldry & Farrington, 2004, p. 4).

### 6.8 The Bully Prevention Challenge Course Curriculum (BPCCC)

Battey (2009) implemented the BPCCC (Haggas, 2006) to students over two 45 min classes, on 4 days of one school week. The program was implemented by trained facilitators, whom included the schools' physical education/health teacher. The program commenced by providing participants with name tags and organizing some warm‐up physical activities. Next, the physical education/health teacher provided participants with information about bullying, such as, identifying and addressing bullying, who to talk to and where to seek support. Subsequent group discussions focused on empathy and understanding each other's differences. Audience participation activities also required the students to engage to represent the number of students whom had been a victim or bully.

### 6.9 Bully Proofing Your School

“Bully‐Proofing Your School” was a comprehensive, school‐based intervention program for the prevention of bullying (Menard & Grotpeter, 2014; Menard et al., 2008; Toner, 2010). The program involved three major components: (1) heightened awareness of the problem of bullying, involving a questionnaire to measure the extent of bullying and the creation of classroom rules related to zero tolerance for bullying; (2) teaching students protective skills for dealing with bullying, resistance to victimization and providing assistance to potential victims by teaching assertiveness skills; and (3) creation of a positive school climate where students were encouraged to work as positive and supportive bystanders (Menard et al., 2008, p. 7).

The primary targets of BPYS were elementary and middle school students. School staff were involved as both secondary targets of intervention (since changes in their behavior was a requirement for the construction of a positive antibullying school environment) and as agents delivering the intervention to students. Teachers were given information and strategies to help them recognize bullying incidents among their students and how to effectively deal with these behaviors (Menard & Grotpeter, 2014).

The intervention in the classes consisted of a classroom curriculum, which included seven sessions of approximately 30–40 min. Each session was delivered by a teacher or by mental health staff. After completion of the classroom curriculum materials, teachers were encouraged to hold weekly classroom meetings during which students could be helped to reflect on their behaviors. Parents were offered information through newsletters. Individual parents of students involved in bullying as either perpetrators or victims were given consultation (Menard & Grotpeter, 2014).

### 6.10 Chinese antibullying intervention

Ju et al. (2009) implemented an antibullying program in a Chinese primary school employing an action research framework. There were two main aims of this intervention program. First, the program aimed to reduce bullying perpetration and victimization both on students' way to, and from, school. Second, the study aimed to investigate practical intervention elements that could be applied nationwide to Chinese primary school children (Ju et al., 2009).

The initial step in this intervention was the training of teachers on the fundamental principles of action research. This training program targeted the following components of educational research: (1) research methodology in education; (2) knowledge of school bullying; (3) components of action research; and (4) intervention skills, such as brainstorming and role‐playing. Second, a 5‐week intervention program was designed and implemented by teachers in classrooms. Components that targeted both victims and bullies specifically were also incorporated into the intervention.

### 6.11 The Confident Kids program

The Confident Kids program is an antibullying intervention designed for early adolescent males who were experiencing anxiety as a result of being bullied at school (Berry & Hunt, 2009). The foundations of the program lie in cognitive‐behavioral therapy, employing both anxiety management techniques and antibullying elements. Based on the “Cool Kids Program” (Lyneham et al., 2003), this intervention program aims to reduce bullying victimization by targeting factors that increase the likelihood of victimization. Therefore, this program focuses primarily on issues such as: self‐esteem, coping strategies; social skills; emotional regulation; and internalizing behaviors.

The program was implemented over a period of 8 weeks, and included student and parent involvement. Students participated in weekly group sessions led by a team of assistant and qualified clinical psychologists. These sessions incorporated a combination of tasks including: skill demonstration; role‐playing; and group discussion. Homework was allocated after each session and participants were encouraged to apply skills acquired in real‐life settings between each session.

Sessions covered a variety of issues, including both cognitive‐behavioral anxiety management techniques and antibullying information. Seven core sessions focused on the following topics: psycho‐education; cognitive restructuring (2 sessions); graded exposure; adaptive coping strategies; improving social skills; and self‐esteem. A final session targeted relapse prevention and provided a general overview of the skills learned throughout the program. Parents participated in sessions that ran parallel to the student program. Group discussions targeted the strategies being taught to student participants and also possible parent factors that could influence effectiveness of intervention for their children, for example, parental anxiety.

### 6.12 Cyberprogram 2.0

Cyberprogram 2.0 is a cyberbullying intervention program that also incorporates elements on school bullying (Garaigordobil & Martínez‐Valderrey, 2015). The intervention is delivered over 19 sessions, and outlines the following four main goals:

- To outline and conceptualize bullying and cyberbullying, including identifying the different roles involved (e.g., bullies, victims, and bystanders).
- To illustrate the consequences of bullying and cyberbullying for all those involved
- To develop coping strategies in order to reduce bullying and cyberbullying behaviors.
- Developing positive social and emotional skills, such as empathy, active listening, anger management, conflict resolution strategies, and diversity tolerance.

A wide range of activities and techniques are used, such as, role‐playing, brainstorming, case studies, and guided discussion. The Cyberprogram 2.0 intervention followed a specific methodological framework, employing four key components for implementation. They are as follows: (1) inter‐session constancy: intervention was delivered in weekly 1‐h sessions; (2) spatial‐temporal constancy: intervention was delivered in the same place and at the same time each week; (3) constancy of adult facilitator: intervention was implemented by the same adult, who same psycho‐pedagogical training, each week; and (4) constancy in the session structure: sessions being with group instruction and activities. There is then a following reflection phase that is led by the adult.

### 6.13 Daphne III

Daphne III was an international antibullying initiative implemented and developed in association with numerous organizations. In this study (Papacosta et al., 2014), school antibullying programs were coordinated in Cyprus by the Association for the Psychosocial Health of Children and Adolescents (APHCA). Other influential “partners” included the Cyprus Ministry of Health, mental health services, Department of Child and Adolescent Psychiatry, Ministry of Education and Culture, and Educational Psychology services. Organizations from other European countries included: Child Line [*Vsi Vaiku Linija*], in Lithuania, and Nicolaus Copericus University, in Poland, were also involved.

The overarching aim of this initiative was to educate 5th and 6th grade primary school students about bullying, and the many different forms it can take (Papacosta et al., 2014). Teachers implemented the program in their classrooms, and were trained by psychology and mental health professionals. There were eleven workshops involved in the program that followed a structured curriculum manual. This manual also provided schools with suggestions and recommendations on ways in with they could prevent, and intervene in, bullying situations.

### 6.14 *Dare to Care: BPYS program

“Dare to Care; Bully Proofing Your School” was a modification of the “Bully Proofing Your School” program (Beran et al., 2004, p. 103), which in turn was modeled on the Olweus Program. This antibullying program placed emphasis on clinical support to victims and perpetrators of bullying in the form of individual and group counseling. It also enabled collaboration with community services. The essence of the program was to encourage accountability for creating solutions among all parties involved in the education system (Beran et al., 2004, p. 104).

The program included several steps. Program facilitators provided to school personnel information and training on issues related to bullying in schools (in a full‐day professional development workshop). This workshop aimed to ensure that the program principles would be reflected in the overall curriculum and would be sustained over time. Information was also given to parents. Then, students, parents and school staff collaborated in the development of a school antibullying policy. This policy had the aim of identifying caring and aggressive behaviors and consequences of those behaviors, but with a focus on reparation rather than punishment. The antibullying policy was posted throughout the school. Finally, the program involved the implementation, on behalf of the teachers, of a classroom curriculum that educated children about the nature of bullying and strategies to avoid victimization. The curriculum included discussion, role‐plays, artwork, books, videos and skits presented to school staff, parents, and other children.

### 6.15 Defeat Bullying

The Defeat Bullying program is a curriculum‐based antibullying program that was published by the National Society for prevention of Cruelty to Children (NSPCC, UK) in 2007 (Herrick, 2012). The program materials were available to download online, as part of a nationwide campaign to reduce bullying perpetration and victimization in UK schools. The overarching aim of the Defect Bullying program is to raise awareness and improve attitudes toward bullying, educate about bullying‐related feelings and emotions, and to develop effective intervention and conflict resolution strategies (Herrick, 2012, p. 85). Based on social identity theory (Tajfel & Turner, 1979), the program aims to establish an in‐class antibullying norm, so that students will be encouraged to adopt this norm, and thus, reduce levels of bullying perpetration and victimization.

There are five key lessons implemented throughout the program, and each incorporates a range of individual, class and group activities (Herrick, 2012). The lessons cover the following five themes: (1) understanding attitudes and values toward bullying; (2) educating about the feelings that occur as a result of bullying; (3) embracing diversity; (4) safety awareness; and (5) encouraging bystanders to get involved in antibullying strategies. The available intervention materials were also reviewed by groups of teachers, and any necessary amendments were incorporated. For example, Herrick (2012) describes that following teacher discussion groups, homework assignments relating to each lesson were developed and implemented. Parents of participating students were also invited to attend an antibullying workshop led by the researcher.

### 6.16 *Dutch antibullying program

The antibullying initiative in the Netherlands was inspired by the Olweus program (Fekkes et al., 2006, p. 639). The program was specifically designed to tackle bullying behavior by involving teachers, parents and students. It offered a 2‐day training session for teachers in order to inform them about bullying behavior and to instruct them about how to deal with bullying incidents in schools. During the intervention period, teachers had access to the training staff for additional advice. Intervention schools were supported by an external organization named KPC, which specialized in training school staff and in assisting schools in setting up new curricula and guidelines. The core intervention program included: (1) antibullying training for teachers, (2) a bullying survey, (3) antibullying rules and a written antibullying school policy, (4) increased intensity of surveillance, and (5) information meetings or parents.

During the intervention, there was careful dissemination of the antibullying program to intervention schools. Also, the researchers provided information about the number of intervention and control schools, which have used the above‐mentioned elements of intervention. Finally, intervention schools were supplied with the booklet “Bullying in schools: how to deal with it” and with a “Bullying Test,” a computerized questionnaire that children could complete anonymously in the classroom.

### 6.17 Dutch Skills for Life

The Skills for Life program is a Dutch universal school‐based behavioral and health prevention program for adolescents aged 13–16 years old (Diekstra, 1996; Gravesteijn & Diekstra, 2013). The program targets prosocial behavior, self‐awareness, social awareness, self‐control, interpersonal skills, and ethical decision making to reduce behavioral and health problems (Fekkes et al., 2016). The program is based on social learning theory and Rational Emotive Behavioral Therapy. As a result, the program aims to reduce bullying by enabling students to learn from each other in a classroom setting through behavioral modeling.

The program is implemented by teachers, who attend two 3‐day training workshops prior to implementation and receive “booster” training sessions throughout the intervention (Fekkes et al., 2016). The intervention is comprised of 25 lessons that are delivered over the course of two academic years. First, four lessons address awareness and handling of thoughts and feelings. Skills such as interpersonal problem solving, emotional regulation, and critical thinking are targeted. There are twelve additional lessons in the 1st year, and nine more lessons in the 2nd year of implementation. These generally focus on skills that are applicable to particular behavioral or health experiences. For example, lessons are aimed at: dealing with bullying; setting and respecting boundaries; substance use; norms and values; friendships; sexuality; suicidal ideation; and conflicts with peers and/or teachers. Various activities are utilized throughout the program, including, active enactment, DVDs, role play, discussion and feedback.

### 6.18 Dynamic Approach to School Improvement (DASI)

The DASI (Kyriakides, Creemers, Papastylianou, et al., 2014; Kyriakides, Creemers, Muijs, et al., 2014) was a whole‐school approach to bullying prevention implemented in several European countries, such as: Cyprus, Greece, UK, Belgium and the Netherlands. This approach draws factors from the educational effectiveness model (Creemers & Kyriakides, 2008, 2012). The intervention targets specific school factors, that is, (1) school teaching policy, (2) school learning environment, and (3) school evaluation. This framework was previously found to improve academic achievement (e.g., Kyriakides, 2008).

At the beginning of the intervention, the research team held training for participating school staff. The theoretical framework was introduced, and a detailed manual was provided. The aim of the handbook was to facilitate school stakeholders to develop strategies and action plans that were specific to the schools' needs (Kyriakides, Creemers, Papastylianou, et al., 2014). Support was offered to each school by the research team throughout the process.

Teacher surveys were distributed prior to implementation in order to highlight specific areas that needed improvement. The next phase of the intervention involved school stakeholders coming together to form cooperative committees with representatives of parents, students, and teachers. These committees then collaborated to develop action plans and strategies to address specific problems in their schools. Committees formulated plans to implement particular intervention components that best suited their specific needs. Therefore, the schools participating did not necessarily implement the same intervention components or activities. Schools were required to retain log books of activities undertaken.

Kyriakides, Creemers, and Papastylianou, et al. (2014) provide an outline of the intervention components implemented in one experimental school involved in their trial. For example, the following are identified as essential elements implemented in order to reduce bullying:

- “Student behavior outside the classroom”—involves developing clear and efficient antibullying policy, increased teacher vigilance in bullying “hot spots” and effective supervision of students.
- Improved school learning environment
- “Rewarding good behavior”—enforcing a system that acts as a nonpunitive approach to antibullying, by motivating students to behave in a prosocial manner.
- “Collaboration and interaction between teachers”—encouraging teachers to work together and communicate effectively about bullying issues in their schools.
- Other intervention components, including, encouraging and supporting peer bystanders; identifying and support “at risk” and vulnerable students; and creating student‐made videos about bullying issues.

### 6.19 *Ecological antibullying program

The Ecological antibullying program examined peer group and school environment processes “utilizing a systemic interactional model with evaluations at each level of intervention” (Rahey & Craig, 2002, p. 283). The overall aim of the program was the creation of a supportive and safe school environment in which firm limits against bullying were established. The specific goals of the program included raising awareness of the problem of bullying, increasing empathy, encouraging peers to speak against bullying and formulating clear rules against bullying.

The 12‐week program was based on the “Bully Proofing Your School” program which was designed to increase the understanding of bullying and decrease the incidence of bullying (Rahey & Craig, 2002, p. 285). The program elements included a psycho‐educational component implemented within each classroom, a peer mediation component and specialized groups for children involved in bullying.

At the school‐wide level, the psycho‐educational program was implemented by psychology students who received training sessions and manuals prior to intervention. Prior to the program, at a school assembly the program was introduced to students. The assembly signaled the formal beginning of the intervention. The classroom programs involved interactive educational approaches such as role playing and puppet techniques. The topics addressed were bullying and victimization, conflict resolution, empathy, listening skills and individual differences (Rahey & Craig, 2002, p. 286).

Individual programs for children involved in bullying were also part of the intervention. The relevant sessions consisted of social skills, listening, empathy training and supportive counseling. Each weekly session lasted 45 min. The program also included intervention at the teacher level. Teacher programs consisted of meetings with teachers to discuss bullying, intervention approaches, and student support for those directly involved in bullying. During the intervention, the program coordinators met with principals and teachers to offer support.

### 6.20 Emotional Literacy Intervention

Knowler and Frederickson (2013) evaluated the effectiveness of an emotional literacy intervention targeted on bullying behaviors to reduce bullying victimization in UK schools. Selected schools were previously implementing the Social and Emotional Aspects of Learning (SEAL; Department for Education and Skills, 2005) program. One of the themes included in the SEAL program is “Say no to bullying” (Knowler & Frederickson, 2013), however the overall program aims to improve students' social relationships, motivation, learning strategies, and holistic school improvement.

The specific emotional literacy intervention implemented and evaluated by Knowler and Frederickson (2013) involved teaching emotional literacy skills to small groups of students (Faupel, 2003). In the current evaluation, the intervention was delivered to groups of “low emotional literacy” and “high emotional literacy” groups distinguished by scores above, or below, median scores on the Emotional Literacy assessment‐pupil form (ELA‐PF; Faupel, 2003). The intervention program employed 12 weekly lessons and was implemented by trained teaching aids (Knowler & Frederickson, 2013). The program consisted of four main concepts: (1) self‐awareness, (2) self‐regulation, (3) empathy, and (4) social skills. Lessons employed a variation of behavioral and cognitive‐behavioral elements (Faupel, 2003).

### 6.21 Empathy training program

This intervention program was developed for children identified as bullies and aimed to increase their empathetic skills in order to reduce their bullying behaviors (Şahin, 2012). The empathy training program was implemented over eleven 75‐min sessions that were based on a curriculum lesson plan developed by the author. Several cognitive techniques were utilized throughout the program, such as: recognizing, evaluating and naming feelings; diadtic, experimental, modeling and role‐playing, in order to improve the students' cognitive abilities in relation to empathy. Each lesson required the students to work together to develop a slogan that emulated the content of the session. The following is an outline of the first 4 weekly lessons, and the associated slogan developed, (for a full outline see: Şahin, 2012, p. 1327; Table 2).

1. Week 1: Students were placed sitting in a “U” shape and asked to introduce themselves to the group. An activity called the “shadow game” (Altinay, 2003) and a psychodrama technique where children were asked to explain to the group what their name means to them, ensued. This was then followed by a short introductory lecture on empathy and empathetic skills using a diadtic approach.
  *Slogan: Be kind, loving and forgiving to each other to lead a happy life*.
2. Week 2: Session was focused on teaching the students about emotional states and creating awareness of emotional sensitivity. Pictures were used to teach students different emotional states, such as, happiness, sadness, anger, fear, courage, and hatred. Diadtic, demonstration and question‐answer techniques were employed. Students were then presented with a number of real‐life examples of each emotional state and were asked to identify the relevant emotion. A take‐home task was provided, where students had to obtain pictures from the media that highlighted different emotions.
  *Slogan: Living without the awareness of feelings is like driving a car with its brakes on*.
3. Week 3: Following the previous session, the outcomes of the take‐home task were discussed. The children were then shown a sketch of various different emotions, and were asked to compare the emotions seen to their own. Students were then given the opportunity to view their own facial expressions for different emotions in the mirror. This was to make them aware of their own feelings and emotions. The take‐home task required students to practice facial expressions associated with different emotional states in the mirror and make a record of what they see.
  *Slogan: One who claims to know everything about the universe but nothing about himself, actually knows nothing*.
4. Week 4: Focused on perception, and specifically, the factors that can affect perception. A short lecture was given, and then students were shown three illusions, and asked to write down what emotions they saw in the pictures. Then, in group discussion, the differences in the emotions identified by individuals were outlined. A take‐home task involved students comparing how they perceive an event (e.g., TV program) to how their parents perceived it.
  *Slogan: We can look at the same thing but view it differently*.

### 6.22 *Expect respect

Expect Respect was a school‐based program that aimed to promote awareness and effective responses to bullying and sexual harassment. The project was developed by Safe Place, the sole provider of comprehensive sexual and domestic violence prevention and intervention services in Austin, Texas (Rosenbluth et al., 2004, p. 211). The program targeted the involvement of all members of the school community in recognizing and responding to bullying and sexual harassment. The overall project design was inspired by the work of Olweus (Rosenbluth et al., 2004, p. 212). Expect Respect consisted of five core program components, namely a classroom curriculum, staff training, policy development, parent education and support services.

The classroom curriculum was based on 12 weekly sessions adapted from a specific manual called “Bullyproof: a teachers” guide on teasing and bullying for use with fourth and fifth grade students' (Whitaker et al., 2004, p. 330). The Bullyproof curriculum was designed to be taught in conjunction with literature typically read by fourth and fifth graders. Although the antibullying curriculum was designed to be implemented by teachers, within the framework of the Expect Respect program, it was jointly led by Safe Place Staff and teachers or school counselors (Whitaker et al., 2004, p. 331). The curriculum aimed to increase the ability and willingness of bystanders to intervene in bullying situations, thus reducing the social acceptability of bullying and sexual harassment. The Bullyproof lessons included writing assignments, role‐plays of how to intervene in bullying situations, class discussions and so on.

With regard to the staff training, a 6‐h training was provided to project staff, counselors, and fifth grade teachers. The training was given by the author of the specific manual and aimed to prepare school personnel to respond effectively to bullying incidents. In addition, 3‐h training sessions were provided once per semester for all personnel, including bus drivers, cafeteria workers, hall monitors and office staff. The training presentation included research on bullying and sexual harassment; strategies to enhance mutual respect among students; practice in using lessons from the curriculum; and methods for integrating the lessons into other subject areas including language arts and health.

School administrators were encouraged to develop an antibullying policy (policy development) in their school to ensure consistent responses by all staff members to incidents of bullying and sexual harassment. Principals were expected to present the policy to school staff, students and parents. In order to facilitate the overall procedure of policy development, Expect Respect staff provided an initial policy template to school administrators (Whitaker et al., 2004, p. 332) and each school was encouraged to expand this initial policy in accordance with the specific needs of their unit.

The Expect Respect program also included parent training. Educational presentations were offered to parents, twice a year, providing information about the project. The information given to parents through these meetings (as well as through parent newsletters sent home) was aimed at enhancing parents' strategies to help children involved in bullying as bullies, victims, bully‐victims, or bystanders.

Further support services were provided such as continuous assistance of school counselors by Safe Place staff. School counselors were given a specialized session on how to deal with students who were repeatedly involved in bullying as either perpetrators or victims. They were also provided with a comprehensive resource manual containing reading and resource materials on bullying, sexual harassment and domestic violence.

### 6.23 fairplayer.manual

The fairplayer.manual is a structured, curriculum‐based antibullying program for Grade 7–9 students (Bull et al., 2009; Wölfer & Scheithauer, 2014). The overarching aim of the intervention is to reduce bullying and relational aggression by improving students' social and moral competencies. The program focuses on raising awareness, changing attitudes, and encouraging bystander intervention.

The program is implemented over 15‐weekly 90 min lessons, and can be delivered either by trained teachers (Bull et al., 2009), or psychologists (Wölfer & Scheithauer, 2014). Intervention lessons employ cognitive‐behavioral techniques and target nine specific topics. The first introductory lesson introduces the program to students, and class antibullying rules are developed. Two following lessons are concerned with raising awareness about bullying‐related issues, such as, the various forms of bullying and the consequences associated with perpetration and victimization. One lesson subsequently focuses on improving students' understanding of their own and peers' feelings. A further two lessons highlight the numerous participant roles involved in bullying, for example, bullies, victims, outsiders (i.e., noninvolved), assistants, and re‐inforcers (Wölfer & Scheithauer, 2014). The latter roles describe different forms of bystanders, those who witness bullying and allow it to happen and those who reinforce bullying behaviors. Social dynamics in the classroom is also addressed in one intervention session. By addressing the different dynamics, networks and norms socially in the class, this lesson aims to improve the classroom climate and encourage co‐operation among students. Another intervention lesson models and promotes bystander intervention in order to encourage noninvolved children to become actively engaged with intervening in bullying situations that they may witness.

Following these core awareness‐raising and knowledge‐improving lessons, participating students undertake five social skill‐training session s. These lessons focus on developing social, emotional, and moral skills of participants, in order to combat bullying. Perspective taking, empathy, and moral dilemmas are just some of the issues that are included. Diversity is the topic addressed in one of the following lessons, where students learn to respect and appreciate diversity. Finally, a concluding lesson brings together all of the issues covered by the intervention and demonstrates ways in which participants can utilize skills and knowledge in their everyday lives.

### 6.24 FearNot!

The FearNot! (Fun with Empathetic Agents to achieve Novel Outcomes in Teaching; Sapouna et al., 2010) was an immersive learning intervention that aimed to reduce bullying victimization. Students from British and German primary schools participated in the virtual learning program for weekly 30‐min sessions over the course of three consecutive weeks. Participating schools were required to have adequate computer facilities in order to be able to run the program.

During intervention sessions bullying scenarios were enacted by male and female 3D animated characters. The content of these scenarios reflected the characters' genders, for example, scenarios involving male characters included more incidents of physical bullying, whereas female characters demonstrated more relational bullying. Following each of the bullying episodes, participants were asked to interact and provide the animated victim of bullying with a suitable coping strategy to prevent future victimization. The program then enabled students to see the outcomes of their suggested strategy. In some circumstances, the animated victim of bullying responded that they did not feel emotionally adequate enough to carry out the suggested coping strategy (e.g., not strong enough to stand up to the bully).

Based on previous research (e.g., Kochenderfer & Ladd, 2000), students were then provided with an indication of how successful their proposed coping mechanism would be in real‐world bullying scenarios. For example, students were provided with a score on a scale of zero (never successful) to ten (always successful; Sapouna et al., 2010). In addition to the computerized program, teachers in intervention schools were provided with a detailed intervention manual. However, during the FearNot! program, teachers were instructed only to assist students with issues of comprehension, and not to guide them on suitable responses to the bullying scenarios.

### 6.25 Fourth R

The Fourth R: Strategies for Healthy Youth Relationships is a dating violence prevention program that targeted bullying perpetration and victimization as secondary outcomes (Cissner & Ayoub, 2014). This curriculum‐based intervention program was based on social learning theory (Bandura, 1978), and was implemented in classrooms by trained teachers during health and physical education classes. Participating teachers completed an intensive 1‐day training session that provided them with the skills to implement the program effectively. Detailed manuals and lesson outlines/materials were provided, and the Fourth R curriculum was integrated into existing health and physical education curricula.

The Fourth R was designed as a 21‐lesson curriculum that incorporates a variety of activities and lessons. Role‐playing, individual, pair and group work, and detailed examples/scenarios of conflict are examples of Fourth R‐style tasks. Program lessons were categorized into the following 3 units: (1) Personal Safety and Injury Prevention; (2) Healthy Growth and Sexuality; and (3) Substance Use and Abuse. Each unit consisted of seven 45‐min lessons. The Fourth R was also designed to be implemented in either gender‐segregated or co‐ed classrooms.

### 6.26 *Friendly Schools Project

“Friendly Schools” was a theoretically grounded program. Its educational techniques (e.g., role modeling, drama activities, skills training, etc.) were based on notions derived from Social Cognitive theory, the Health Belief Model and Problem Behavior theory (Cross et al., 2004, 2011). An interesting aspect of this program is that it was based on the results of a systematic review (Cross et al., 2004, p. 187), which provided a set of key elements to be included in the final intervention strategy. The program targeted bullying at three levels: (a) the whole‐school community, (b) the students' families, and (c) the fourth and fifth grade students and their teachers.

With regard to the whole‐school intervention component, in each school, a Friendly Schools Committee was organized with key individuals (e.g., a parent representative, a school psychologist, a school nurse, teaching staff) who could co‐ordinate and successfully sustain the antibullying initiative. Each committee was provided with a 4‐h training, designed to build members' capacity to address bullying. Each member was provided with a specific strategy manual. The manual was a step‐by‐step guide on how to implement the antibullying initiative. It included among others the Pikas “Method of Shared Concern” and the “No Blame” approach (Cross et al., 2011; Pikas, 2002).

With regard to the family intervention component, this included home activities linked to each classroom‐learning activity. Parents were also provided with 16 skills‐based newsletter items (eight for each year of the intervention) that aimed to provide research information on bullying as well as advice to parents on what to do if their child was a perpetrator or a victim of bullying behavior.

Moving on to the Grade 4 and 5 classroom curricula, the Friendly Schools curriculum consisted of nine learning activities per year. The curriculum was offered by trained teachers in three blocks of three 60‐min lessons, over a three‐school‐term period. The learning activities aimed to promote awareness of what was bullying behavior; to help students to become assertive and talk about bullying with teachers and parents; and to promote peer and adult discouragement of bullying behavior.

Finally, the Friendly Schools program offered manuals to teachers. The teacher manuals were designed to be entirely self‐contained so as to maximize the likelihood of teacher implementation. Friendly Schools project staff also provided teacher training (a 6‐h course) for all intervention teachers.

### 6.27 *Granada antibullying program

This program was a pilot antibullying program with the following aims: (a) to establish children's involvement in bullying within different participant roles/categories; (b) to reduce the number of students involved in the phenomenon as bullies, victims and bully‐victims; (c) to increase the number of students who are categorized as noninvolved in bullying, through the enhancement of prosocial skills; and (d) to identify the threats to fidelity of the program and establish the validity of the pilot program with the possibility of replicating it in future (Martin et al., 2005, p. 376). Forty‐nine sixth graders from one Spanish primary school in Granada participated in the program.

The program designers gathered information about the social, educational and economic background of the school, of the students' families and the community in general. That was done during 3 meetings/seminars of 3 h each. Parents, teachers and members of the educational team attended those meetings. Through these meetings, it was established that the program should target interpersonal relationships of the children. It was decided that the program would be curriculum‐based as part of the normal program of the school. It was decided that the program would be implemented by one of the researchers because the teachers did not have enough qualifications to do it and because of lack of time and resources for teacher training. Parents and teachers were provided with information about bullying (a dossier/file) that they could use to discuss the problem of bullying with children. Also, teachers could attend the intervention program so that later they would be able to implement it by themselves. Parents were invited to attend some talks on bullying that would be given by the implementation team so that the program could be continued outside the school. The program was implemented for 5 months at the classroom level (30 sessions; 3 sessions per week with one tutor, i.e., one of the evaluators).

During the first 5 sessions, the tutor informed the children about peer bullying. Topics covered in the first 5 sessions involved issues such as concept of bullying, types of bullying, how to identify it, individual and group differences in bullying, and classroom rules against bullying. From the 6th to the 21st sessions, the program emphasis was on the emotional and social abilities of the children. Several topics were covered such as: identification and expression of emotions during bullying situations; communication abilities; ability to pose questions; ability of children to give and receive complements and complaints; ability to say no in life; ability to ask for a change of behavior; and ability to solve interpersonal problems. From the 17th to the 21st sessions, the program placed emphasis on mediation.

From the 22nd to the 25th sessions, the program emphasis was on human rights. Several topics were covered such as: freedom and equality, respect of private life, respect for other people's belongings, and respect for others' opinions. Similarly, from the 26th to 30th sessions, the emphasis was on moral education. During the whole program (sessions 1–30), there was also an emphasis on the inhibition of impulsivity and enhancement of reflexivity. For the enhancement of reflexivity, the program designers used a specific program called “Programa de Intervencion para Aumentar la Attention y la Reflixividad” [PIAAR] developed by Gargallo (2000) (see Martin et al., 2005, p. 378). This focuses on cognitive techniques that aim to inhibit impulsivity and enhance self‐control. The program also included role‐playing, peer mediation, guided discussion, brainstorming, and drawings.

The authors acknowledge several problems with the implementation of the program such as: little involvement by parents and teachers; implementation of the program lessons during recess time or during the physical education program; lack of time to cover all the topics; no second follow‐up because of difficulties of following the children; problems with the size and selection of the sample; the instrument they used; and possible contamination of results because of the way they categorized the children (Martin et al., 2005, p. 382). These pitfalls could easily be spotted. For example, the evaluators indicate that they implemented the program with the most aggressive sixth graders who had the worst interpersonal problems (Martin et al., 2005, p. 738). This made it difficult to know whether any changes in bullying in the experimental condition were attributable to the effectiveness of the program or to regression to the mean. Also, even though they distributed a self‐report questionnaire, they categorized children based on those questionnaires only after teachers' suggestions.

### 6.28 *Greek antibullying program (1)

The Greek antibullying initiative was a 4‐week intervention program that aimed to minimize both bullying and victimization. The conceptual framework of the Greek antibullying program was based on the theoretical model proposed by Salmivalli in 1999 (Andreou et al., 2007, p. 696), according to which changing an individual's behavior (e.g., the bully's behavior) entailed motivating not only the particular person but also the rest of the group members (participant roles' approach).

The program was embedded within the wider curriculum of the fourth‐, fifth‐, and sixth‐grade classrooms and consisted of eight instructional hours, each hour corresponding to one curricular activity. The curricular activities were presented to students by their classroom teachers who received training beforehand. The teacher training consisted of five 4‐h meetings and aimed to increase awareness of the bullying problem and its seriousness as well as to raise teachers' self‐efficacy in implementing the program (Andreou et al., 2007, p. 697).

The Greek antibullying curriculum was divided into three parts in accordance with the three main theoretical axes proposed by Salmivalli in 1999, namely: (1) awareness‐raising; (2) self‐reflection; and (3) commitment to new behaviors (Andreou et al., 2007, pp. 697–698).

In line with the first axis (awareness‐raising), small‐group and whole‐class discussions were conducted (over three instructional hours) that aimed to increase students' awareness of the bullying problem. Corresponding materials included a real snap‐shot from the playground, a story entitled “A new friend” and students' own drawings. In line with the second theoretical axis (self‐reflection), two instructional hours involving classroom discussions were conducted. These discussions placed emphasis on the participant roles that students took in the bullying process. Corresponding materials involved each students' completion of open‐ended sentences. Through this activity students were intended to reflect on critical issues around the causes, benefits, feelings, and consequences of adopting different roles. In line with the final axis (commitment to new behaviors), three instructional hours of small‐group and whole‐class discussions were conducted concerning different ways of approaching or solving the peer‐conflict situation and the formulation of class rules. Corresponding materials involved an open‐ended comic‐strip for group completion to find a solution to the bullying situation presented in the relevant story.

### 6.29 Greek antibullying program (2)

This antibullying program was implemented in Greek elementary schools during the academic year 2011/2012 (Tsiantis et al., 2013). The school‐based program incorporated many elements and was implemented by teachers. Participating teachers attended a 2‐day training seminar before implementation began. A teacher's manual (Tsiantis, 2011) was also provided and outlined the detailed and systematic procedures involved in the intervention. Throughout the program teachers were provided with additional support from two mental health professionals whom acted as program co‐ordinators.

The program comprised of 11 weekly workshops that were implemented for two 45‐min class periods (90‐min in total). Class activities included group discussions, games and the formation and signing of class antibullying rules (Tsiantis et al., 2013). Parent meetings were also organized to increase parent participation with the intervention. The first meeting provided parents with information about the intervention program and bullying issues. During the second parent session, students presented the achievements they had made during the intervention.

### 6.30 Inclusive

The INCLUSIVE program is a whole‐school restorative approach to bullying prevention and intervention (Bonnell et al., 2015). The program involves creating an “action group” within each participating school in order to combat bullying. These groups are comprised of a minimum of six students and six members of staff, with at least one representative from senior management, teaching, support, and pastoral staff. Each action group is appointed an external expert facilitator for the duration of the intervention. It is the facilitators' role to provide ongoing support and training to each member of the action group. Action groups were required to meet regularly throughout the intervention year, approximately once every half term.

The INCLUSIVE intervention was designed to include several core standardized intervention components, including staff training in restorative practices, and a student social and emotional skills curriculum. However, the program also allows for schools to adapt the intervention according to school‐specific needs. These needs were established using a needs assessment survey distributed to year 8 students prior to commencement of the intervention. This survey aimed to establish student views on bullying and aggression in their schools, while providing information regarding school engagement and connectedness, perceptions of safety/risks, social support and social skills, relationships, and teaching in personal, social and health (PSHE) classes. Results of the needs assessment survey were then employed by the action group to tailor the INCLUSIVE intervention to target specific needs. The action groups also utilized this information to review and improve schools' existing policies, procedures and schemes (e.g., peer mediation and “buddying” schemes).

In relation to the core components of the INCLUSIVE intervention, all school staff were provided with introductory training in restorative practices by their affiliated expert facilitator. A minimum of twenty school staff were also required to attend intensive training provided by a specialist training provider. Restorative practices, such as “Circle Time,” were taught to staff to improve school climate and student‐staff communication. This technique involves teachers and staff sitting together in a circle discussing various emotional, social, and curricular issues. Each member of the circle is considered a valued contributor, and all inputs are treated equally. Circle time aims to support student communication and promote positive relationships. Another restorative technique used in the INCLUSIVE program was “formal conferencing,” which aimed to deal with serious bullying and aggressive incidents directly. Formal conferencing involves bringing together teachers, parents and students to establish appropriate punishment and ways in which the harm caused can be repaired. This approach emphasizes a nonjudgmental and inclusive environment so that both victims and perpetrators of bullying and/or aggression are involved.

Year 8 students also completed 5–10 h of social and emotional skills training throughout the process of the INCLUSIVE intervention. These lessons were based on the Gatehouse Project curriculum and could be delivered as either stand‐alone modules or integrated into existing academic curriculums. Modules covered included: (1) Establishing respectful relationships; (2) Emotion management; (3) Understanding and creating trusting relationships; (4) Exploring others' needs and avoiding conflict; and (5) Maintaining and repairing relationships.

### 6.31 *KiVa

The name of this project is an acronym of the expression “Kiusaamista Vastaan” which means “against bullying.” The word “kiva” in Finnish means “nice” and this is why this acronym was chosen for the specific antibullying initiative in Finland. Regarding the overall perspective of the program, the KiVa project included a universal and an indicated intervention (Kärnä et al., 2011a, 2011b, 2013; Nocentini & Menesini, 2016; Salmivalli et al., 2007). The universal intervention referred to efforts made to influence the group norms while the indicated intervention referred to the way in which specific cases were handled in schools through individual and group discussions between the teacher and the students involved (Salmivalli et al., 2007, p. 6).

The KiVa program included a large variety of concrete materials for students, teachers, and parents. It also utilized the Internet and virtual learning environments (e.g., computer games against bullying) aiming in this way to enhance students' attitudes against bullying. Also, students received their own personal user ID, which they could use as a password before the completion of each web‐based questionnaire on bullying. KiVa included 20‐h student lessons, which were carried out by student teachers. The lessons involved discussions, group work, short films about bullying, and role‐playing exercises. After each lesson, a class rule was adopted, based on the central theme of the lesson.

A unique feature of the KiVa program was the use of an antibullying computer game. The game involved five levels and the teacher always activated the next level of the game after the relevant lesson was completed. Students were able to begin using the game after the third lesson; the second level of the program was played after the fifth lesson, and so on until the end of the school year. Each level of the computer game included three components that were named as “I know,” “I can,” and “I do.” In the first component, students were informed about basic facts on bullying. In the second component, the “I can”‐component, students moved around in the virtual school and faced different challenging bullying incidents. Finally, the third component was used to encourage students to make use of their knowledge and skills in real life situations.

Another important element of the KiVa project was the teacher training. Teachers were also provided with vests that they could use during playtime while supervising the school yard. This simple technique aimed to enhance teachers' visibility in the schoolyard and to signal that bullying was taken seriously in the school. Also, all teachers carrying out the KiVa program could seek advice from a web‐based discussion forum, where they could share experiences and ideas about bullying with other colleagues.

Within the school framework, the program also facilitated the use of a peer support group for victims of bullying. The classroom teacher was expected to arrange a group with 2–4 classmates—those who were pro‐social and had high status in the class—who were expected to provide support to victimized students, thus sustaining healthy peer relationships. An interesting element in the KiVa program is that it incorporated both punitive and nonblame approaches when dealing with perpetrators of bullying. Half of the school teams were instructed to use more punitive approaches (e.g., what you have done is wrong and it has to stop right now) while the rest of the school teams were instructed to use no‐blame approaches in their discussions with children (e.g., “your classmate is also having a hard time and this is why he behaves like that; what could we do to help him?”). There was also co‐operative group work among experts when dealing with children involved in bullying.

Finally, the KiVa program involved parents. A parents' guide was sent to the home and provided information about bullying and advice on how parents could be involved to reduce this problem. Information nights for parents were also organized and provided.

### 6.32 Lead Peace Intervention

The Lead Peace intervention is based on a resiliency conceptual framework (Resnik, 2000), thus, aims to reduce youth problem behaviors using an assets‐based approach (Harpin, 2011; Sieving & Widome, 2008). The intervention was developed as a school‐based “service learning and health education” program to reduce risk of violence and school failure in middle school students (Sieving, 2006). Developed from the Points of Light Youth Leadership curriculum for 9th to 12th grade students (Sieving, 2006), the program was adapted for use with Grade 6–8 students (Harpin, 2011).

The core curriculum targets factors on three levels: (1) environmental (e.g., adult resources and supports, family norms and behaviors, peer norms and behaviors, school/community opportunities and social connectedness); (2) personal (e.g., attitudes, beliefs, perceived norms, emotional distress); and (3) behavioral (e.g., social and emotional skills, coping behaviors, school performance). The program aims to reduce risky health and social behaviors (e.g., interpersonal aggression, physical fighting, bullying) in order to promote positive and reduce risky behaviors. The curriculum is implemented for 3 years, and can be delivered in two “doses”: (1) Lead Peace program (basic)—includes 15–20 intervention lessons each year; or (2) Lead Peace plus program—includes 30 intervention lessons, 15–20 additional community service hours, and health education and family outreach activities.

### 6.33 Lunch Buddy Mentoring program

The Lunch Buddy mentoring program was a school‐based antibullying program that aimed to reduce bullying victimization in elementary school children (Elledge et al., 2010). The program was based on previous research that suggests youth mentoring can be utilized as an effective prevention technique (Dortch, 2000). In comparison to peer‐mentoring antibullying program, the Lunch Buddy program employed college student mentors based on prior success of college student mentoring aggressive children (Cavell & Hughes, 2000).

Mentors were provided with training prior to implementation of the program and participated in weekly meetings throughout the program. Children were identified as potential participants using a self‐ and teacher‐report victimization index. The self‐report School Experiences Questionnaire (Kochenderfer & Ladd, 2000) and teacher ratings of child victimization due to physical, verbal and relational aggressive were combined to create this index. School principals also collaborated with counselors to identify potentially suitable candidates. Eligible participants were then matched with same‐sex college student mentors, based on the availability of mentors during the mentees scheduled lunchtimes. Mentors visited the mentees twice a week, over the course of 5–6 months. During these visits mentors were required to sit with their mentee and their peers during lunchtime. Each mentor was also required to complete a log sheet after each visit.

### 6.34 Media Heroes

Chaux et al. (2016) evaluated the effectiveness of the cyberbullying prevention program “Media Heroes” [*Medienhelden*] on reports of traditional school bullying. The Media Heroes program is based theoretically on the Theory of Planned Behavior (Ajzen, 1991) and the social context of participant roles in bullying (Salmivalli, 2010). The program aims to reduce cyberbullying perpetration by enhancing empathy, increasing awareness and knowledge about what constitutes cyberbullying, the safety risks associated with Internet activity, and by providing assertive and useful methods in which bystanders can intervene in cyberbullying (Chaux et al., 2016).

There are two versions of Media Heroes: (1) a short version implemented over four 90‐min lessons that take place in one school day; and (2) a long version that is implemented over 15‐weekly 45‐min lessons (Schultze‐Krumbholz et al., 2012). Intervention activities include, role‐playing, class debates, news and film content, group learning and student‐parent presentations (Chaux et al., 2016). Measures of both traditional‐ and cyber‐bullying were implemented in this evaluation, due to the significant overlap in the prevalence of these behaviors.

### 6.35 NoTrap!

Noncadiamointrappola (Let's Not Fall into a Trap), or NoTrap!, is a web‐based antibullying program that has been developed, implemented and refined over several studies (Menesini et al., 2012; Palladino et al., 2012, 2016). Initially implemented in two Italian schools in 2008, the program involves students actively engaging in the development of a website promoting antibullying (Menesini et al., 2012). A selected number of students per school are provided with training and enroll as online peer‐educators. These students acted as online moderators of an antibullying forum, regulating discussion threads and responding to users' questions and concerns (Menesini et al., 2012). In addition, peer‐educators also conducted face‐to‐face awareness raising workshops and meetings with their classmates, to highlight the key issues surrounding traditional‐ and cyber‐bullying (Palladino et al., 2016).

Subsequent editions of the NoTrap! program incorporated additional elements based on findings from previous evaluations. For example, Palladino et al. (2012) placed more emphasis on: (1) victims' roles and victim support, (2) involving bystanders, (3) greater involvement of teachers in antibullying activities, and (3) creation of a Facebook group to supplement online materials. The third revision of the NoTrap! program incorporated standardization of the face‐to‐face antibullying activities led by peer educators (Palladino et al., 2016). New peer‐led activities involved group work that targeted empathy and problem‐solving skills (Palladino et al., 2016).

### 6.36 *Olweus Bullying Prevention Program

The OBPP was a multilevel program aiming at targeting the individual, the school, the classroom and the community level. Apart from marked mass‐media publicity, the program started with a 1‐day school conference during which the problem of bullying was addressed between school staff, students, and parents. This signaled the formal commencement of the intervention. Two different types of materials were produced: a handbook or manual for teachers (entitled “Olweus” core program against bullying and antisocial behavior') and a folder with information for parents and families. The program also included: (1) CD‐program that was used for assessing and analyzing the data obtained at the pre‐test period, so that school‐specific interventions could then be implemented; (2) a video on bullying; (3) the Revised Olweus Bully/Victim Questionnaire and (4) the book “Bullying at school: what we know and what we can do.”

The antibullying measures mainly targeted three different levels of intervention: the school, the classroom and the individual. At the school level, the intervention included:

- Meetings among teachers to discuss ways of improving peer‐relations; staff discussion groups.
- Parent/teacher meetings to discuss the issue of bullying.
- Increased supervision during recess and lunchtime.
- Improvement of playground facilities so that children have better places to play during recess time.
- Questionnaire surveys.
- The formation of a coordinating group.

At the classroom level the intervention included:

- Students were given information about the issue of bullying and were actively involved in devising class rules against bullying.
- Classroom activities for students included role‐playing situations that could help students learn how to deal better with bullying.
- Class rules against bullying.
- Class meetings with students.
- Meetings with the parents of the class.

At the individual level the intervention included:

- Talks with bullies and their parents and enforcement of nonhostile, nonphysical sanctions.
- Talks with victims, providing support and providing assertiveness skills training to help them learn how to successfully deal with bullying; also, talks with the parents of victims.
- Talks with children not involved to make them become effective helpers.

An interesting feature of the OBPP is that it offered guided information about what schools should do at both the intervention and the maintenance period. The Olweus program demands significant commitment from the school during the 'introductory period' which covers a period of about 18 months. Later the methodology acquired by the staff and the routines decided by the school may be maintained using less resources … Yet, even for the maintenance period, the program offers a point by point description of what the school should do to continue its work against bullying in accordance with Olweus methodology (Olweus, 2004c, p. 1). Also, at the school level training was offered to the whole school staff, with additional training provided to the coordinators and key personnel. These were responsible for coordinating the overall antibullying initiative in their school. The program also included cooperation among experts and teachers (e.g., psychologists) who worked with children involved in bullying.

### 6.37 Positive Action program

The Positive Action Program is a generalized school‐based “well‐being” program (Lewis et al., 2013). The program targets both distal (e.g., school climate and teacher classroom management) and proximal (e.g., students' thoughts, feelings, and self‐efficacy) facets are targeted in order to impact a range of health‐ and behavioral‐related outcomes (Li et al., 2011). The program is based on three core elements.

First, the Positive Action philosophy. Based on the theory of self‐concept (Combs, 1962; Purkey, 1970; Purkey & Novak, 1996) and a Positive Psychology (Frederickson, 2000; Seligman & Csikszentmihalyi, 2000) approach, the philosophy emphasizes positive feelings about the self, to encourage positive behaviors toward others (Flay & Allerd, 2010). Second, the Thoughts‐Actions‐Feelings Circle concept is used throughout the program to illustrate the reinforcing cycle of thoughts, feelings and actions. This is delivered to outline that positive thoughts lead to positive actions, positive actions in turn lead to positive feelings, which then reinforce positive thoughts. Third, a strict six‐unit curriculum that involves daily lessons, interactive learning and social‐emotional skill development.

The PA curriculum is designed to be adapted for kindergarten to Grade 12 students, and is based on six key concepts: (1) self‐concept; (2) social and emotional positive actions for managing oneself responsibly; (3) positive actions relating to a healthy body and mind; (4) honesty with oneself; (5) getting along with others; and (6) continuous self‐improvement (Lewis et al., 2013). The intervention program also involves teacher, parent/family and community training. Schools implementing the PA program receive support from developers throughout implementation by training, manuals, school‐wide climate development, counselors, family classes, and individual consultations for staff with a PA implementation coordinator.

### 6.38 Preventure and Adventure CBT

The Preventure and Adventure intervention programs were part of two 2 year longitudinal projects that targeted adolescent alcohol use and bullying behaviors (Topper, 2011). Intervention components were primarily personality‐targeted cognitive behavioral therapy (CBT) for “high risk” students. Participants were screened prior to taking part in the intervention for four individual personality domains: (1) hopelessness; (2) anxiety‐sensitivity; (3) sensation seeking; and (4) impulsivity. Students who were classified as being “high risk” on any of the four domains were invited to participate, and assigned to one of four potential intervention workshops. These intervention sessions were CBT‐based and were aimed at each of the four personality domains. Thus, a student who scored highly on the impulsivity measure was assigned to the impulsivity‐focused CBT session. For participants that scored above the mean on multiple measures, they were assigned to the session that corresponded to the personality domain that they deviated the most from standardized scores.

High risk students in each school were randomly assigned to either the intervention or control condition, as were “low risk” students, for comparison. The Preventure study took place between 2005 and 2007, and either a chartered counseling psychology, an experienced special needs teacher, or a master‐level research assistant implemented intervention workshops. In comparison, the Adventure study took place between 2007 and 2009, and although the intervention sessions followed the same procedure, they were implemented by trained teachers in each school.

### 6.39 *Pro‐ACT+E program

Pro‐ACT+E was a universal, multidimensional program that aimed to prevent bullying in secondary schools (Sprober et al., 2006). It involved a cognitive‐behavioral approach to the problem of bullying and victimization by building up prosocial behavior. The program was universal: it did not involve specific work with perpetrators or victims of bullying. However, it included both teacher and parent training and a 2‐h classroom discussion with students about violence problems. The program offered curriculum materials that aimed to increase awareness in relation to the problem of bullying and placed emphasis on specific issues such as classroom management and classroom rules against bullying.

### 6.40 *Progetto Pontassieve

The program was delivered in a period of 3 years, and it consisted of two main parts. During the 1st two years it was delivered more at the school level whereas the 3rd year was more at the class and individual level (Ciucci & Smorti, 1998). During the 1st year a training course for teachers took place addressing psychosocial risks for children and bully‐victim problems. At the end of the training, a study was conducted to reveal how serious was the problem of bullying and what were its characteristics. The 2nd year of the intervention included a counseling service for each individual who was affected by bullying.

The intervention took place in the 3rd year and was based on the use of two different methods: Quality Circles, where pupils had to cooperate to find practical solutions to their problems, with the use of the Interpersonal Process Recall which consisted of the recording of one Quality Circle and discussion about it. The other method used was Role Playing conducted in small groups with subsequent class discussions, which helped students to examine possible strategies to face and overtake bullying problems. The aims of both of these methods were to make students aware that they could intervene in an efficient way to reduce bullying.

### 6.41 *Project Ploughshares for Peace

Project Ploughshares Puppets for Peace (P4 program) was an antibullying program that aimed to educate elementary school students about bullying and conflict resolution (Beran & Shapiro, 2005, p. 703). The P4 program used puppets and a 30‐min script. Using three‐feet, hand‐and‐rod puppets, two puppeteers enacted a story that involved direct and indirect bullying, as well as a successful resolution to this scenario. These behaviors occurred among two female puppets and a male puppet friend.

After watching the play, students were invited to identify the bullying behaviors. During the discussion, four main strategies—presented as “4 Footsteps”—to deal with bullying were suggested to pupils: (1) ignore, (2) say stop, (3) walk away, and (4) get help. The show took approximately 45 min and aimed to increase children's awareness about which behaviors could be categorized as bullying and to show various strategies that children who were bullied and/or who witnessed bullying could use to discourage it (Beran & Shapiro, 2005, p. 703).

### 6.42 Rational Emotive Behavioral Education (REBE) and ViSC

Trip et al. (2015) implemented a dual program consisting of REBE (Trip & Bora, 2010) and ViSC social competence (Strohmeier et al., 2012) elements. These components were combined to address both social and emotional factors involved in bullying and positive youth development (PYD). This program approaches bullying from a sociological perspective, including factors on the individual, family, peer, classroom, and school levels (Espelage & Horne, 2008; Swearer & Espelage, 2011).

ViSC social competence program is a systemic approach to antibullying that targets students, teachers and parents (Strohmeier et al., 2012). Implemented by teachers in the classroom, the program comprises several intervention units that aim to: (1) foster empathy and perspective training, (2) enhance responsibility, and (3) improve students' behavioral responses to bullying (Trip et al., 2015, p. 733).

REBE elements employed by Trip et al. (2015) on the other hand, target specific elements of aggression that are lacking in the ViSC units. Based on the theory of Rational Emotive Behavioral Therapy (Ellis, 1962), the REBE elements of the intervention program target the difference between desire and reality (Trip & Bora, 2010) and anger. The REBE program activities target specific elements of anger, specifically, anger triggers, personal experiences of anger and the consequences of anger (Trip et al., 2015).

### 6.43 Restorative Whole‐school Approach (RWsA)

The RWsA (Hopkins, 2004; Morrison, 2002) was a school‐based antibullying initiative that employs a restorative justice inspired philosophy. Hence, the program focuses on creating a positive school environment to prevent bullying in the long‐term, rather than a short‐term disciplinary and punishment approach (Wong et al., 2011). The program had three core goals: (1) to create a positive and harmonious school learning environment; (2) implement an interactive classroom curriculum; and (3) encourage an effective partnership between teachers, students, parents and relevant professionals.

A whole‐school antibullying nonpunitive ethos and policy is implemented as the core of the intervention (Wong et al., 2011). This policy aims to establish a positive school environment in order to combat bullying‐related risk factors. The curriculum lessons incorporated elements on various issues, including, empathy, assertiveness, coping, problem‐solving, and conflict resolution.

### 6.44 Resourceful Adolescent Program (RAP)

The RAP is a classroom‐based CBT intervention designed for adolescents aged 12–15 years of age (Stallard et al., 2013). The program is a depression prevention program, however, bullying problems were included as secondary outcomes. The program incorporates a detailed manual and student workbooks, and was implemented over nine sessions, of approximately 50–60 min each. The core components include: psycho‐education, helpful thinking, identifying personal strengths, keeping calm, problem solving, support networks, and keeping the peace. The program was designed to flexible and adaptable to participating schools' varying busy timetables.

### 6.45 *S.S. Grin

The Social Skills Group Intervention (S.S. GRIN) was a school‐based program that aimed to help children enhance their social skills. S.S. GRIN was designed as a social‐skills training intervention for peer‐rejected, victimized, and socially anxious children. It could be applied to an array of problems that are social in nature (e.g., aggression, low self‐esteem, depression, social anxiety, social withdrawal) not just bullying (DeRosier & Marcus, 2005, p. 140). The authors argued that the program went beyond the most common social‐skills training (De Rosier & Marcus, 2005, p. 141) by emphasizing the cognitive aspects of relations and emotions. That is, children were not only taught prosocial skills, but they were also taught, on the cognitive level, how to identify negative perceptions and behaviors in an effort to help children to regulate their own emotions as well as enhance their coping skills.

Overall, the program was a combination of social‐learning and cognitive‐behavioral techniques, used to help children build social skills and positive relationships with peers. It was a highly structured, manualized program (DeRosier, 2004, p. 197) with a number of sessions containing scripts and activities to undertake. Each session included didactic instruction combined with active practice such as role‐playing, modeling and hands‐on activities (De Rosier, 2004, p. 197). The children participated in group sessions for eight consecutive weeks. Each session lasted approximately an hour. The groups were led by each school's counselor and an intern, who were trained and supervised by one of the program instructors (De Rosier & Marcus, 2005, p. 143).

### 6.46 School‐based Drama program

This school‐based antibullying program was based on drama (Owens & Barber, 1998) and social cognitive theories (Bandura, 1978). The main aim of this project was to design and implement a drama‐based program to improve social relationships and social/emotional well‐being in children, which in turn may help to reduce bullying (Joronen et al., 2011). Targeted concepts included: empathy; social competence; student‐teacher interaction; child–parent interaction; and recognition of values/emotions.

This program was developed by the combined efforts of researchers, drama experts and teachers. It was implemented in‐class by trained teachers and school nurses over a period of 6 months. Teachers and school nurses attended a 2‐day seminar and received two drama handbooks, however, there was no manual or fixed program outline provided. Support was provided through email communication between teachers and researchers for the duration of program implementation. Teachers conducted one drama session per month with their class. These sessions covered a variety of topics, including, bullying, friendship, loss of a friend, supporting a bullied peer, tolerance, and child abuse.

### 6.47 School‐wide Positive Behavioral Interventions and Supports (SWPBIS)

SWPBIS was a universal behavioral intervention program that targets school‐level factors in order to improve school climate and promote positive student and staff behaviors (Waasdorp et al., 2012). Instead of following a specific antibullying curriculum, SWPBIS aimed to reduce bullying by targeting schools' discipline and behavioral management strategies. A SWPBIS team in each school organized and facilitated the intervention implementation.

These teams were responsible for developing a set of “positive expectations” for the school. These were a number of statements that outlined what the school expected in relation to student and staff behavior, for example, “be responsible, respectful, and ready to learn” (Waasdorp et al., 2012, p. 150). Posters highlighting the expectation statements were then displayed all around participating schools, both in classrooms and outside of classrooms, and are positively reinforced using reward systems. Furthermore, data from student surveys and discipline referrals were employed throughout the intervention to inform teachers of potential bullying “hot spots” that require increased supervision and monitoring. School staff also received training on classroom management and how to respond consistently and effectively to bullying. Additionally, students identified as being “high risk” or vulnerable to bullying behaviors or victimization were provided with selective intensive intervention.

### 6.48 School bus antibullying intervention

This intervention program was a universal antibullying program designed to reduce the prevalence of bullying behaviors on school buses (Krueger, 2010). The program was purposefully developed and utilizes materials and content from the “Take a Stand, Lend a Hand, Stop Bullying Now!” tools that are available free of charge.

The intervention was implemented with elementary school children over five consecutive days, during the final 20‐min of the school day. Lessons were delivered by the school's social worker and principal to two groups (kindergarten to 2nd grade students, and 3rd to 5th grade students) of participants. The program followed this format from days 2–5, however, on day 1, all participants completed the introductory lesson together. The school‐bus antibullying program primarily utilized DVD materials from the “Take a Stand” content. These video clips depicted cartoon characters engaging in different bullying scenarios.

On day 1 (i.e., the introductory lesson) an overview of school bullying and related issues, including bystander intervention, was provided to participants. The associated DVD clip depicted a male character physically bullying another child in the playground while other students watched. Participants then discussed the clip in groups, and were introduced to the “Three Steps to Stop Bullying Chart.” This technique involves three steps, *Stop, Help*, and *Tell*, that bystanders can take if they witness bullying.

On each subsequent day, a new DVD clip was shown to participants and the *Stop, Help*, and *Tell* concepts were revisited. The school's social worker or principal led discussion groups by posing questions to the students concerning the feelings and emotions experienced by the victim of bullying, potential coping strategies that the victim could use, and possible bystander behaviors. Participants also shared their previous experiences with similar situations. Furthermore, using the *Stop, Help*, and *Tell* paradigm, participants brainstormed potential ways to tell a bully to stop behaving in a certain manner, ways to help the victim and appropriate trusted adults that they can tell about the situation.

### 6.49 Second Step

The Second Step: Student Success Through Prevention is a middle school Social‐Emotional Learning (SEL) program that aims to reduce bullying, peer victimization, physical aggression, homophobic name‐calling and sexual violence (Espelage et al., 2013, 2015). The intervention curriculum is taught in‐class by trained teachers. Lessons are interactive and engaging, requiring students to take part in whole‐class, small group and individual work. A take home task is also given after each lesson to reinforce skills learned. DVDs are also used to accompany and enrich lesson content.

The 6th grade Second Step curriculum involves 15 weekly lessons on various social and emotional skills and bullying‐related topics. The following outlines the curriculum: (1) empathy and communication—five lessons; (2) bullying—two lessons; (3) emotion regulation (e.g., coping with stress)—three lessons; (4) problem‐solving—two lessons; and (5) substance abuse prevention—four lessons.

Each lesson has clearly outlined learning objectives to reduce problem behaviors and increase prosocial behaviors. For example, lessons on bullying target the peer context by increasing knowledge, improving attitudes, and encouraging bystander intervention in order to reduce bullying perpetration and victimization. Students are educated about the differences between types of bullying, importance and responsibilities of bystanders in preventing bullying and a number of positive bystander behaviors are modeled. The 7th grade Second Step curriculum involves a similar lesson structure, with some slight changes. The intervention is delivered over 13 weekly lessons, and cyber‐bullying and sexual harassment issues are incorporated into bullying modules.

### 6.50 Shared Concern

Wurf (2012) assessed the effectiveness of the whole‐school approach to bullying intervention and prevention, with a particular emphasis on Pikas' (2002) nonpunitive method of shared concern. The Pikas method of Shared Concern is a teacher, or counselor, implemented intervention, that is divided into five key stages. First, the intervener identifies the students involved in bullying and talks with them individually. These discussions aim to provide nonpunitive and constructive options for both bullies and victims (Wurf, 2012). The second and third stages involve providing empathy and ongoing support to the victims of bullying. Finally, the fourth stage incorporates a mediation session between bullies and victim(s). A conflict resolution approach to prevent bullying is agreed upon and implemented by all involved. The fifth and final stage occurs during the follow‐up period, whereby the teacher or counselor monitors the involved students to ensure that the bullying has stopped.

### 6.51 *Short Intensive Intervention in Czechoslovakia

The antibullying intervention in Czechoslovakia was inspired by the OBPP and borrowed elements from it, such as the Olweus videocassette on bullying (Rican et al., 1996, p. 399). The Olweus bullying questionnaire was used to measure several aspects of bullying within the schools. A peer nomination technique was also used to identify bully and victim scores. The relevant results from both measurement scales were presented to teachers in the intervention schools to increase awareness of the problem of bullying. The program researchers discussed with the teachers “possibilities of an individual approach to the bullies as well as to the victims” (Rican et al., 1996, p. 399).

As another intervention element, teachers were instructed to introduce relevant ethical aspects into the curriculum where possible: the ideal of knighthood was suggested for history classes and the ideal of consideration for the weak was introduced in sentences used for dictation and analysis (Rican et al., 1996, p. 400). Another element of the intervention involved the use of a method called “class charter.” Specifically, children were asked to indicate how they would like their teachers and other classmates to behave toward them as well as how students should behave toward teachers and among themselves. The final aim of this classroom activity was the construction of a set of rules and principles, which was then signed by all pupils in the classroom and placed there in a visible position. Finally, the Olweus video‐cassette on bullying was shown to children and was used as a means of promoting the antibullying idea in the school.

### 6.52 *Short Video Intervention

This antibullying strategy, involved a single viewing of an antibullying video, entitled Sticks and Stones, and aimed to examine its effects on secondary school students' views of, and involvement in, bullying. The program aimed to examine both attitudes toward bullying and the actual behavior since “it would not be unreasonable to propose that these attitudes will influence actual behavior” (Boulton & Flemington, 1996, p. 334). The program involved only one school that had no prior antibullying policy.

The video presented pupils (either in groups or on their own) talking about bullying, their views about this phenomenon and their personal experiences of bullying. The video also involved a number of bullying scenes (see Boulton & Flemington, 1996, p. 337 for examples).

### 6.53 Social and Emotional Training (SET) intervention

This intervention program was a school‐based SET mental health program for Swedish school children (Kimber et al., 2008). The SET program was primarily focused on mental health, but also targeted other aspects of participants' lives, such as bullying. Both internalizing and externalizing aspects of child mental health are addressed.

Trained teachers delivered the program over the course of two academic years. Intensity of program implementation varied according to the age of students. Junior students (i.e., grades 1–5) received the program in 45‐min sessions twice a week, while senior students (i.e., grades 6–9) completed one 45‐min session per week. Program developers provided each participating teacher with detail manuals for implementing the program with each grade and grade‐specific student workbooks. Role‐playing and modeling tasks covered many themes, including: social problem solving; conflict management; dealing with strong emotions; and resisting peer pressure. Teachers were also supervised once a month during the 1st year of implementation, and students were encouraged to practice skills both at school and at home.

### 6.54 Social Norms Project

Lishak (2011) implemented an antibullying program based on social norms theory (Perkins, 2003) with middle school students. The program was implemented over a period of 12 weeks and was developed based on student responses to an anonymous web‐based survey and student discipline and suspension reports (Lishak, 2011). Student surveys collected information regarding perceptions of bullying in the school and results were then relayed to participants via weekly lessons, assemblies, posters, and media content throughout the school. Data from school discipline, suspension and visitation logs were collated to estimate the prevalence of bullying and school violence.

### 6.55 *Social Skills Training (STT) program

STT was a program specifically designed to support “chronic victims” of bullying (Fox & Boulton, 2003, p. 237). The general aim of the program was to help children improve their social skills, therefore reducing a child's individual risk of victimization (Fox & Boulton, 2003, p. 234). The program involved an 8‐week course during which children learnt how to use both problem‐solving and relaxation skills, how to think positively, how to modify their nonverbal behavior and how to use some verbal strategies such as “fogging” and “mirroring” (Fox & Boulton, 2003, p. 235).

During the program, victims of bullying were gathered in groups of five to ten and were exposed to the aims of the program for 1 h/week. Two trainers delivered the 1‐h sessions throughout the program. The 1st week was dedicated to children introducing each other and listening each other's problem. The next two sessions dealt with issues of friendship and aimed to help children form strong friendships (e.g., having conversations; asking to join in), while the fourth session dealt with issues of body language: teaching children how to modify their nonverbal behavior in a way that would protect them from being victimized. During the fifth session children learned how to be assertive while in the next two sessions children were taught how to deal with the bully. The eighth session signaled the end of the program.

### 6.56 *SPC and CAPSLE program

This evaluation compared the effects of two intervention packages with a treatment‐as‐usual condition (Fonagy et al., 2009). Nine schools were randomly allocated to the two experimental and one control (treatment‐as‐usual) conditions after a stratified allocation procedure, which was used to stratify schools based on the percentage of low‐income students (indicated by students' free‐ and reduced‐lunch status). In the experimental conditions, the full intervention was offered for 2 years (the efficacy phase) with a limited 3rd year of intervention (the maintenance phase).

The first experimental condition involved a “School Psychiatric Consultation” (SPC), a manualized protocol that aims to address mental health issues of children with disruptive behavioral problems, internalizing problems, or poor academic performance. SPC was a school‐level intervention focused on individual children. Three child psychiatry residents, supervised biweekly by a senior child psychiatrist, delivered mental health consultation following the SPC manual for 4 h/week. The psychiatric residents attended weekly school resource meetings and consulted directly with teachers, parents and other school personnel, through classroom observations and meetings, providing 140 consultations for 65 students in year 1 and 97 consultations for 45 students in year 2.

The second experimental condition involved the implementation of CAPSLE (“Creating a Peaceful School Learning Environment”), a manualized psychodynamic approach addressing the cocreated relationship between bullies, victims and bystanders. In contrast to SPC, CAPSLE represents a whole‐school intervention approach. It aimed to modify the educational and disciplinary school climate. A CAPSLE team drawn from school staff in the pilot project led implementation in the two intervention years using a training manual. In year 1, teachers received a day of group training, students received nine sessions of self‐defense training, and the CAPSLE team consulted with school staff monthly. Year 2 started with a school‐wide half‐day refresher self‐defense course, and consultation continued with counselors, teachers and adult/peer mentor programs. In year 3 (the maintenance phase), self‐defense training continued as in year 2.

CAPSLE includes several antibullying materials that can be used by teachers such as a Teacher Discipline Manual (used in the teacher training), a Student Workbook, Buttons and Magnets and Patches (used as a way of reinforcing of desirable student behavior), Parent Warning Notes (notifying parents about specific problem behavior of the child) as well as antibullying videos that can be used during the physical education lessons (and videos that can be used by parents). CAPSLE also includes the Gentle Warrior Program, a 12‐week curriculum specifically designed for physical education teachers. For CAPSLE, intervention fidelity was assessed using a teacher self‐report measure that required teachers to state the frequency with which various CAPSLE program components were implemented.

### 6.57 Standard CBT and CBT plus media program

This intervention program combined elements of standardized CBT and DVD bullying‐related materials in order to reduce bullying perpetration and victimization among elementary school children (McLaughlin, 2009). The standardized CBT lessons were delivered by a trained counselor, and focused on bullying and aggression relation issues. Two experimental groups were employed, one of which received only the CBT lessons, and the other completed the CBT lessons and were shown the bullying DVDs.

The program was implemented over 4 weekly lessons that followed a strict outline. In week 1, the lesson focused on defining bullying, identifying bullying roles and different forms of bullying, and exploring the possible characteristics of bullies, victims, and bystanders. Week 2's lesson was concerned with establishing the consequences of bullying for all those involved, including the bully, victim and bystanders. Empathy for victims of bullying was also developed. Activities included creating feeling lists, and participating in role plays. Lesson three aimed to promote bystander intervention by developing awareness and knowledge of appropriate responses to bullying, suitable ways to intervene, and promoting assertiveness. Classes are taught using educational and informative posters. The final lesson, in week 4, aimed to outline the gender differences in bullying, why these occur, and ways to combat gender‐specific forms of bullying. In their classes, students establish class antibullying rules and are taught about the support available in school to stop bullying.

In addition, students in the CBT + media experimental group watched three DVDs that highlighted the issues outlined in the weekly lessons. The DVDs that were shown are as follow: (1) *Let's Get Real*, which shows young people talking about their personal experiences of bullying; (2) *The Deepest Hurt*, that depicts girls role‐playing various scenarios of relational aggression; and (3) *The Broken Toy*, a dramatization of the damage bullying can cause. Following the videos, students engaged in group discussions led by the counselor about the issues illustrated in each DVD.

### 6.58 *Stare bene a scuola: Progetto di prevensione del bullismo

This intervention was based on the curriculum activities and the whole school approach because it tried to involve all people in a school (Gini et al., 2003). The program was delivered to 6 schools and included several activities. Teachers were first trained in 3 days on “cooperative learning” and in particular on the Jigsaw technique. Teachers then had an on‐going supervision once every 15 days. The intervention in the class lasted 4 months with two meetings a week. The intervention was directed toward the following areas: (1) awareness of the body and what it feels; (2) emotional awareness; and (3) bullying awareness. These areas were dealt with in each of the sessions, starting from the first one. For each thematic area, several activities were conducted and several methods were used.

### 6.59 Start Strong

“Start Strong: Building Healthy Teen Relationships” was a school‐based curriculum focused teen dating‐violence prevention program (Williams et al., 2015). The program was implemented over 2 years in four experimental schools (that implemented the program) and four comparison schools (that did not implement the program). Schools were matched based on: school size, percentage of students eligible for free school lunches, race/ethnicity, and socioeconomic status. The effectiveness of the program was measured for outcomes that included the perpetration and victimization of teen dating‐violence, bullying and sexual harassment.

### 6.60 *Steps to Respect

The Step to Respect program aimed to tackle bullying by: (1) increasing staff awareness; (2) fostering socially responsible beliefs; and (3) teaching social‐emotional skills so as to promote healthy relationships (Frey et al., 2005, p. 481). The program included staff and family training manuals, a program guide and lesson‐based curricula for third‐ through sixth‐grade classrooms (Hirschstein & Frey, 2007, p. 7).

Components at a whole school level consisted of an antibullying policy and procedures, staff training and parent meetings, all aiming at sharing understanding of bullying and its consequences and increasing adult awareness, monitoring, and involvement. At the classroom level, the proposed activities consisted of teaching friendship skills, emotion regulation skills, identifying types of bullying, teaching prevention strategies and peer group discussion. The aim was to improve peer relations and reduce the risk of victimization, assess level of safety and recognize, report and refuse bullying. At the individual level, students involved in bullying were approached and coached based on the “Four‐A Responses”: affirm behavior, ask questions, assess immediate safety and act.

The S to R training manual consisted of an instructional session for all school staff and two in‐depth training sessions for counselors, administrators, and teachers. There were also videos accompanying the program. With regard to staff training, there were two levels of training: all school staff received an overview of the program goals and principal aspects of the program (program guide). Teachers, counselors, and administrators received additional training in how to coach students involved in bullying, based on behavioral skills training, cooperative learning and role‐playing.

The student curriculum comprised skills and literature‐based lessons delivered by third‐ through sixth‐grade teachers during a 12–14‐week period. The intervention consisted of 10 semi‐scripted skills lessons with topics such as joining groups, distinguishing reporting from tattling and being a responsible bystander.

Finally, with regard to the parent intervention, administrators informed parents about the program and the school's antibullying policy and procedures. Parents could also benefit from other resources such as letters provided to them and newsletters describing whole‐school antibullying activities undertaken at school.

### 6.61 Strengths in Motion (SIM)

The SIM (Rawana et al., 2011) program was a strength‐based whole school antibullying intervention. There were several components involved in the program, all of which centered around a strength‐based approach. This technique involves highlighting and enhancing individuals' strengths in order to develop positive mental health (Duckworth et al., 2005). In the context of the present evaluation, Rawana et al. (2011) requested that each participating school allocated one room as a designated intervention resource room. In the first instance, this room acted as a “Good Start Centre” (p. 287) where new students to the school were provided with two half‐day orientation sessions prior to starting school. Part of these orientation sessions was individualized strength assessments. It was predicted that by providing new students with guidance on how to best use their strengths to integrate successfully into school life the likelihood of future bullying and victimization would be reduced.

The second use of the intervention room was as a “Cool Down & Prevention,” where students experiencing behavioral or emotional problems could go to calm down. Staff were on hand to prevent the behaviors from escalating and offer helpful advice. The room also acted as an alternative to suspension from school, whereby students could be mandated to spend a certain number of days in the “Good Choices Room.” An ambassador's club for students identified as being at high risk for bullying perpetration or victimization was also held in the resource room. Finally, mental health professionals provided student and parent workshops and staff received tailored training on the strength‐based approach to bullying prevention and intervention.

### 6.62 Take the LEAD (TTL)

The TTL (Domino, 2011, 2013) program was designed to increase the social competencies of participants in order to reduce bullying behaviors. The intervention is based on SEL and PYD theories.

Various social and emotional skills are targeted during the 16‐weekly lesson curriculum, including: (1) Self‐awareness; (2) Self‐management; (3) Social‐awareness; (4) Relationship skills; (5) Decision making; (6) Problem solving; and (7) Leadership. Trained teachers taught TTL lessons during normal class periods on a weekly basis. Participating teachers were trained on the skill‐based curriculum by the developers of the TTL program. During training, teachers were taught about specific learning objectives and goals of the intervention program, and also about the lesson plans and activities involved in “Take the LEAD.” Information evenings for parent were also held as part of the TTL intervention and aimed to raise parents' awareness of key social‐emotional issues.

Each of the sixteen TTL lessons involved specific learning objectives and goals. Lessons involved a combination of knowledge and skill development and an application component, so that participants were given the opportunity to apply skills in real‐world settings. For example, the “Communication skills” lesson aimed to “explore elements of communication that enhance interpersonal skills and foster positive relationships (Domino, 2013, p. 432). During this lesson students brainstormed ideas about effective and positive communication techniques and were then required to practice these skills (e.g., eye contact, active listening and showing empathy) in pairs. Finally, participants were required to practice these techniques in an interview with a classmate, and later with a parent.

### 6.63 *Toronto antibullying program

The Toronto antibullying program was inspired by the OBPP (Pepler et al., 2004, p. 125). It was based on the understanding that bullying is a problem that extends far beyond the individual children; it involved the peer group and the teachers, as well as the parents of children (Pepler et al., 2004, p. 127). The program included several preventive elements implemented at the school, parent, and classroom levels, as well as additional work with specific students involved in bullying as perpetrators or victims.

The level of implementation of the program varied across the intervention schools. However, in all intervention schools three critical elements were found: staff training, codes of behavior and improved playground supervision. At the school level an emphasis was placed on developing a positive code of behavior among students, engaging teachers, and promoting positive playground interactions. At the parent level, information nights were held during which parents were informed about the problem of bullying in their school. Also, information about the program and its objectives was sent home. At the classroom level, children were involved in developing classroom rules against bullying. Further classroom activities aimed to change students' attitudes and to promote healthy relationships among peers. At the individual level, children involved in bullying as perpetrators or victims received specialized intervention through consultation and though engaging their parents. Follow‐up monitoring of these cases helped school authorities to establish that bullying incidents were terminated or discontinued.

### 6.64 *Transtheoretical‐based Tailored antibullying program

This antibullying initiative involved “transtheoretical‐based tailored programs that provided individualized and interactive computer interventions to populations of middle and high school students involved in bullying as bullies, victims and/or passive bystanders” (Evers et al., 2007, p. 398). The intervention involved only three 30‐min computer sessions during the school year for the students and a 10‐page manual for staff and parents with optional activities. According to the program designers, the transtheoretical model is “a theory of behavior change that applies particular change processes like decision‐making and reinforcement to help individuals progress at particular stages of change” (Evers et al., 2007, p. 398).

Intervention materials included the “Build Respect, Stop Bullying” program, which is a multicomponent, internet‐based computer system (Evers et al., 2007, p. 402). Students initiated the program by running a multimedia CD which brought them to the program website. Students could use the program by creating a login name based on personal information and a password. Once the students registered for the program, logged in and consented to be involved in the intervention study, they were given instructions on how to proceed. This multi‐media program also included short movies (videos) of students giving testimonials about bullying (Evers et al., 2007, p. 403).

Other elements of the program included: (1) a 10‐page family guide, sent to children's homes, which provided brief information about the multi‐media program and its relation to the antibullying initiative; and (2) a 10‐page staff guide, which included general information about bullying and how to support student change, classroom activities and information on how to work with parents. Teachers were not provided with any training.

### 6.65 Utrecht Healthy Schools

The Utrecht Healthy Schools program was a comprehensive educational program that targeted adolescent health behaviors (Busch et al., 2013). The integrated program aims to improve various different health‐related behaviors exhibited by Dutch secondary school students, such as, nutrition, exercise, sexual health, substance and alcohol use, smoking behaviors, bullying, and excessive use of television, gaming and Internet use. The program was implemented as a whole‐school approach and consisted of five key components.

First, participating schools implemented a “healthy school” policy outlining a zero‐tolerance attitude toward risky or violent behaviors, such as alcohol use, smoking or bullying. Second, the program aimed to create a healthy school environment by offering healthy options in the canteen, removing vending machines, ensuring proper sports facilities, hosting alcohol‐free school parties and implementing a smoke‐free school yard. In the third instance, the program aimed to involve parents in intervention activities by providing parent workshops and/or take‐home activities for students. Finally, curriculum materials focused on personal skill development and the program aimed to incorporate public health services into the intervention program.

### 6.66 *Viennese Social Competence Training program (ViSC)

The ViSC aimed to provide students “with systematic theoretically‐based guidance in becoming responsible and competent actors in conflict situations” (Atria & Spiel, 2007; Yanagida et al., 2019). It was specifically designed for disadvantaged adolescents aged fifteen to nineteen who were considered at risk for future problems (Atria & Spiel, 2007, p. 179). The theoretical basis of the programs drew its main ideas from social information processing theory and from research that approached the problem of bullying as a group phenomenon (Gollwitzer et al., 2006, p. 126).

The ViSC program consisted of thirteen lessons which were divided into three phases: (1) impulses and group dynamics; (2) reflection; and (3) action. The first phase, entitled “impulses and group dynamics,” consisted of six lessons and the main aim was to enhance students' competence in dealing with critical situations by teaching them how to look at social situations from different perspectives using vignette stories, discussions and role‐plays. The second phase, *reflection*, involved one lesson during which pupils reflected on what had been learned in the first phase of the program.

The last phase, *action*, consisted of six lessons during which the trainer asked students to define how they wanted to benefit from the remaining lessons. The trainer collected students' individual ideas, evaluated them and—along with the students—put them in practice in alignment with the global goal of the program: enhancing pupils' social competence. The third phase of the program was flexible and it could involve several projects suggested by pupils such as a movie production, a work of art, the organization of a party, and so on. This flexibility was allowed and was, in fact, a main feature of ViSC because organizing such projects “involves a variety of critical situations, in which alternative, nonaggressive response options can be probed, rehearsed, and evaluated for success” (Gollwitzer et al., 2006, p. 126).

Based on the design of the program, the training of students was conducted by specialist trainers, not their teachers. The trainers participated in instruction workshops and were also supervised during the training by the ViSC developers' team at the University of Vienna (Gollwitzer et al., 2006, p. 127). According to the principles of the program, it was essential for the trainer to avoid receiving any information about individual students offered by teachers; students' assessments should be based on standardized diagnostic measures (Atria & Spiel, 2007, p. 184). Moreover, the training was conducted during regular class time and teachers were advised to attend the lessons, so that the program was taken seriously by the students. ViSC has been implemented and evaluated three times: by Gollwitzer (2005), by Atria and Spiel (2007) and by Gollwitzer et al. (2006).

### 6.67 Youth‐led program

The Youth‐led program (YLP; Connolly et al., 2015) was a generalized middle school violence prevention program. This program was developed by a community agency, and involved training high school students to lead violence prevention workshops with middle school students in order to increase the latter's knowledge and attitudes of peer aggression and victimization.

Experienced mental health professionals were employed to select and supervise male and female high school students that would become “youth leaders.” These students received training in afterschool sessions on skills and knowledge of peer aggression. Topics covered included bullying perpetration and victimization, but also peer aggression, violence, and harassment.

The final sessions of this training required the youth leaders to create two individualized presentations; one covering bullying and the other discussing general aggression. Mixed gender pairs of youth leaders then conducted these presentations in middle school classrooms under the supervision of a mental health worker. These presentations lasted for approximately 45 min each.

### 6.68 *Youth Matters

The Youth Matters program used “a curricular and a modified systemic approach to bullying prevention” (Jenson & Dieterich, 2007, p. 287). The aim of the curriculum was to strengthen peer and school norms against antisocial behaviors by addressing critical issues (issue modules) such as the difference between teasing and bullying, building empathy, risks and norms surrounding aggression and so on. The curriculum also aimed to promote skills (skill modules; structured skills training sessions) that students could use in order to stay safe at school, cope with bullying, enhance their social skills and improve their peer relationships. To address systemic issues associated with bullying, curriculum modules terminated with the development of classroom or school‐wide projects, which placed emphasis on the negative consequences of bullying for students.

The curriculum consisted of 10‐session modules. Each module included a 30–40‐page story, the content of which was directly linked to the structured skills training sessions. When looking at the implementation of the program, all curriculum materials were “language sensitive”: translated into Spanish for use in the three Spanish‐speaking classrooms included in the evaluation. Youth Matters curriculum modules were offered to fourth and fifth graders. According to Jenson and Dieterich (2007, p. 287), grades 4 and 5 were selected “based on an appropriate fit between developmental ability and curricula.”

The Youth Matters program was based on a theoretically grounded curriculum. The curriculum was based on theoretical constructs derived from the Social Development Model. The latter integrated perspectives from three theories (i.e., social control theory, social learning theory and differential association theory) and proposed that four factors inhibit the development of antisocial development in children. These were: (1) bonding or attachment to family, schools and positive peers; (2) belief in the shared values or norms of the above‐mentioned social units; (3) external constraints or consistent standards against antisocial behavior; and (4) social, cognitive and emotional skills that can be seen as protective tools for children to solve problems and perform adequately in social situations. The Youth Matters curriculum addressed each of these four core areas.

### 6.69 Zero program

The Zero antibullying program is based on the idea that bullying is predominately a version of proactive aggression (Roland et al., 2010). The program aims to create a school environment that prevents these forms of proactive aggression. The intervention places the majority of responsibility for bullying prevention and intervention with the adults within the school environment (Roland et al., 2010). School staff were required to define clear standards of positive prosocial behavior among the students and to ensure that these standards are met. Thus, the adults within the school context adhere to a “zero tolerance” policy toward bullying. Another key feature of the intervention is that students are instructed to treat all school property appropriately and respectfully and the intervention philosophy is carried into classroom activities and standards also.

During the intervention, class teachers engage their respective classes in active discussions about issues relating to bullying in adherence with the intervention guidelines. The preventative function of the Zero program takes both a direct and indirect approach (Roland & Galloway, 2004). Teachers are also expected to be vigilant and visible in school corridors and playgrounds during nonclass time and follow intervention procedures when dealing with specific instances of bullying (Roland et al., 2010). When particular instances of bullying are identified, the victim is first approached and takes part in a few sessions with trained staff being comforted and assured. Parental involvement also occurs at this point. Finally, the perpetrators are invited to attend meetings and conflict resolution occurs under a restorative justice model.

### 6.70 Zippy's Friends

Zippy's Friends is a universal school‐based program for children aged 6–8 years old (Holen et al., 2013; Mishara & Ystgaard, 2006). The overarching aim of the program is to develop and improve participants' coping strategies in order to reduce and prevent psychological problems. Zippy' Friends has been funded by the global suicide prevention organization “Befrienders International,” and is now distributed internationally by the nonprofit group “Partnership for Children.”

The intervention is delivered over the course of 24 weekly lessons, that are implemented by classroom teachers. The program is based around six stories of the imaginary character “Zippy,” three children, and their families and friends. A structured curriculum outline for each lesson allows participants to engage and discuss the various themes that emerge in each of the stories. Themes that are incorporated include: emotions; communication; friendships; conflict resolution; loss and change.

Teachers are provided with a detailed manual for the program and are required to guide their classrooms through the intervention while also encouraging active engagement with the content. Typical activities that are involved in the Zippy's friends program include: drawing, role‐playing, performing exercises, play and dialogue.

## 7 RESULTS OF SYSTEMATIC REVIEW

In addition to the newly identified studies (*n* = 88), primary evaluations (*n* = 53) discovered by Farrington and Ttofi (2009) are also included in the present systematic review, giving a total of 141 studies. However, this updated systematic review has excluded evaluations that used an “other” experimental‐control design (*n* = 13). Next, a detailed explanation is provided about studies which were excluded from the current review and justifications for this decision.

### 7.1 Studies excluded because of missing information

A certain amount of statistical information is needed in order to produce meaningful effect sizes in a meta‐analysis. We estimated an antibullying program's effectiveness as the difference between the experimental and control groups on bullying outcomes, either measured as the percentage of bullies/nonbullies or victims/nonvictims or based on mean scores on measurement instruments before and after implementation of the intervention.

However, 21 studies identified by our systematic review did not present sufficient effect size information, and so the primary authors of these publications were contacted. We were able to obtain relevant information for the majority of these studies, but three authors were unable to provide required statistics and seven did not respond to our email communication.

Thus, 10 studies had to be excluded from our meta‐analysis because of a lack of information regarding quantitative outcomes. These relate to: Gradinger et al. (2015); Harpin (2011); Kyriakides et al. (2014); Lewis et al. (2013); Lishak (2011); Low and Van Ryzin (2014); van der Ploeg et al. (2016); Sahin (2012); Schroeder et al. (2012); and Wurf (2010). In the previous review by Farrington and Ttofi (2009), 44 out of 53 evaluations provided sufficient information on quantitative outcomes.

### 7.2 Studies excluded because of nonindependent samples

One further stipulation of a meta‐analysis is that the final samples must be independent of one another (Borenstein et al., 2009; Ellis, 2010). Overlapping samples are statistically dependent, and thus the variance of the summary effect size produced by the meta‐analysis would be under‐estimated (Wilson, 2010). Therefore, before conducting our meta‐analysis we ensured that all samples were independent of one another.

This issue of nonindependent samples was particularly relevant for the multiple evaluations of the KiVa antibullying program. Our thorough systematic searches identified 16 potentially includable studies presenting evaluation data from implementation of the KiVa program (i.e., Ahtola et al., 2012, 2013; Garandeau, Lee, et al., 2014, Garandeau, Poskiparta, et al., 2014; Haataja et al., 2014; Hutchings & Clarkson, 2015; Kärnä et al., 2011a, 2011b, 2013; Nocentini & Menesini, 2015; Noland, 2011; Sainio et al., 2012; Salmivalli et al., 2012; Williford et al., 2012, 2013; Yang & Salmivalli, 2015). For a description of each of these studies, see Table 7.

**Table 7:** Description of KiVa studies

*Study*	*Description*
Ahtola et al. (2012)	Evaluated the effectiveness of KiVa on teachers': (1) self‐evaluated efficacy to combat bullying, (2) understanding of bullying, and (3) confidence in the effectiveness of the KiVa program. Data was drawn from 238 teachers in 62 schools involved in the large‐scale evaluation of KiVa in Finland (Kärnä et al., 2011a, 2011b, 2013)
Ahtola et al. (2013)	Explore the relationship between implementation adherence of the KiVa program and teachers' perceived support received from head teachers. Sample drawn from Kärnä et al. (2013) second phase KiVa evaluation, employing 93 Grade 1–3 teachers from 27 Finnish schools
Garandeau, Lee, et al. (2014)	Utilize data from large‐scale RCT of KiVa program (Kärnä et al., 2011) to compare the effectiveness of the program for popular and unpopular bullies
Garandeau, Poskiparta, et al. (2014)	Employ data from 65 intervention schools involved in the second phase of KiVa evaluation (Kärnä et al., 2013). Analyse the difference between the “Confronting Approach” and the “Non‐Confronting Approach” for dealing with individual incidences of bullying
Haataja et al. (2014)	Examine how the implementation fidelity of KiVa lessons influences the program's overall effectiveness using data from Grade 1–6 students involved in the large‐scale evaluation of KiVa in Finland (i.e., Kärnä et al., 2013)
Hutchings and Clarkson (2015)	Outlines the introduction of the KiVa program in UK schools and the results of a pilot evaluation, however, no control group was employed
*Kärnä, Voeten, Little, Poskiparta, Alanen, et al. (2011)	Report results from the national nonrandomized trial of KiVa in 888 Finnish schools
*Kärnä, Voeten, Little, Poskiparta, Kaljonen, et al. (2011)	Randomized controlled trial evaluating phase one of KiVa implementation with students in Grades 4–6 in 78 Finnish schools. Intervention began in May 2007 (pretest) and finished in 2008 (posttest)
*Kärnä et al. (2013)	Randomized controlled trial evaluating phase two of KiVa implementation with students in Grades 1–3 and Grades 7–9 from 73 Finnish schools. Intervention began in May 2008 (pretest) and finished in May 2009 (posttest)
*Nocentini and Menesini (2016)	Randomized controlled trial evaluating the effectiveness of the KiVa program with Grade 4 to 6 students from 13 Italian schools. Intervention and data collection began in September to October 2013 and finished in May to June 2014
Noland (2011)	Analyse the effects of the KiVa program on adolescents' perceptions of peers, and experiences of depression and anxiety using data from the Kärnä et al. (2011) evaluation
Sainio et al. (2012)	Explore the differences in the effectiveness of the KiVa program to reduce same‐ and other‐sex victimization using data from Kärnä et al. (2011) evaluation
Salmivalli et al. (2012)	Using data from Kärnä et al. (2011) evaluation of the KiVa program, study evaluates the effectiveness of the program on different forms of being bullied
Williford et al. (2012)	Journal publication of Noland (2011) thesis
Williford et al. (2013)	Employ data from Kärnä et al. (2011) and Kärnä et al. (2013) large‐scale evaluations of the KiVa program to assess the effectiveness of the program to reduce cyber‐bullying perpetration and victimization
Yang and Salmivalli (2015)	Using data from a previous longitudinal evaluation of the KiVa program (Salmivalli, 2010) to assess the impact of the program on bullies, victims, and bully‐victims

* Included in meta‐analysis.

However, following further screening, only four of the aforementioned studies were subsequently included in the systematic and meta‐analytic review (i.e., Kärnä et al., 2011a, 2011b, 2013; Nocentini & Menesini, 2016). These four studies presented independent results of the KiVa program from the initial nationwide evaluation in Finland. Kärnä et al. (2011a) used an age cohort design with adjacent cohorts and reported the initial results from the nationwide implementation in Finland. Second, Kärnä et al. (2011b) reported the results from the RCT with Finnish students in grades 4–6, and Kärnä et al. (2013) reported results for students in grades 1–3 and 7–9. In addition, Nocentini and Menesini (2016) reported the results of the implementation and evaluation of KiVa in Italian schools. The remaining 12 publications relating to the KiVa program utilized data from the RCT evaluation in Finland (i.e., Kärnä et al., 2013 or Kärnä et al., 2011b) but explored different facets of the program's effectiveness.

Four studies identified in our systematic searches replaced evaluations included in the earlier review. For example: (1) Menard and Grotpeter (2014) was a continuation of the Menard et al. (2008) evaluation; (2) Cross et al. (2011) was a republication of the Cross et al. (2004) evaluation included in the previous review; (3) Jenson et al. (2013) and Jenson et al. (2010) presented data from additional follow‐up points to the Jenson et al. (2007) evaluation; and (4) Frey et al. (2009) used an age cohort design to evaluate follow‐up effects from the earlier Frey et al. (2005) study. In cases such as these, the most recent publication, or the publication with the most statistical information, was included in the meta‐analysis.

Ten studies (published both before and since 2009) were identified as reporting the effectiveness of an antibullying program from the same sample, or were repeat publications of earlier studies (e.g., DeRosier, 2004 and DeRosier & Marcus, 2005; Domino, 2011 and Domino, 2013; Espelage et al., 2013 and Espelage et al., 2015; Jenson et al., 2013 and Jenson et al., 2010; and Menesini et al., 2012; Study 2 and Palladino et al., 2012). In these instances, the most recent publications were selected, and as a result, five studies were excluded from the meta‐analysis.

### 7.3 Included studies

Therefore, 128 studies are included. Table 5 summarizes the intervention programs and methodological components of the 79 newly identified studies that are included in the present systematic review. For details of the remaining 49 studies please refer to Farrington and Ttofi (2009).

### 7.4 Moderator analysis

The following moderators were selected a priori for further analysis, under the descriptive label (i.e., location of intervention, publication type, publication year), design label (i.e., evaluation method and unit of allocation/randomization), and the program heading (i.e., name of intervention, COI, and program specificity). Results of these moderator analyses analogous to the analysis of variance (ANOVA) are presented in Sections 8.5.1 to 8.5.7 of the present report.

#### 7.4.1 Evaluation method

The primary moderator chosen for further analysis was evaluation method. Specifically, whether the evaluation was conducted using a RCT, quasi‐experimental with before and after measures (BA/EC) or age cohort (AC) design.

Overall, in relation to bullying perpetration outcomes, 36 evaluations used RCT designs, 31 used BA/EC designs and 14 used age cohort designs. However, due to some evaluations reporting data for multiple independent samples, a total of 40 effect sizes were estimated for bullying perpetration outcomes from RCT designs. A further 36 were estimated from BA/EC designs and 14 effect sizes came from evaluations using age cohort designs.

For bullying victimization outcomes, overall, 33 evaluations used RCT designs that gave 37 independent effect sizes for bullying victimization and 37 evaluations used BA/EC designs and gave 42 independent effect sizes. Similar to perpetration outcomes, 14 evaluations used age cohort designs to evaluate the effect of antibullying programs on bullying victimization outcomes.

#### 7.4.2 Location of intervention

Evaluations included in the present analysis were conducted in many different countries around the world. However, there were only a few countries in which multiple evaluations of antibullying programs had been published.

Specifically, in the following countries only one evaluation was included in the present report: Austria (i.e., Yanagida et al., 2019); Brazil (i.e., Silva et al., 2016); China (i.e., Ju et al., 2009); Czechoslovakia (modern day Czech Republic and Solvakia; i.e., Rican et al., 1996); Hong Kong (i.e., Wong et al., 2011); Ireland (O'Moore and Milton, 2004); Malaysia (i.e., Yaakub et al., 2010); Romania (i.e., Trip et al., 2015); Sweden (i.e., Kimber et al., 2008); Switzerland (Alsaker & Valkanover, 2001); South Africa (Meyer & Lesch, 2000); and Zambia (Kaljee et al., 2017).

If these evaluations were to be included in further moderator analysis, we would be examining the differences based on only one sample and effect size. Therefore, moderator analysis was conducted only between locations in which multiple evaluations of antibullying programs had been conducted.

So, of the 100 evaluations included in our meta‐analysis of school‐based antibullying programs, the majority (80 for perpetration, 84 for victimization) were conducted in one of 12 different countries. With respect to bullying perpetration outcomes, these countries were as follows: Australia (*n* = 2); Canada (*n* = 6); Cyprus (*n* = 3); Finland (*n* = 6); Germany (*n* = 5); Greece (*n* = 2); Italy (*n* = 11); Netherlands (*n* = 3); Norway (*n* = 8); Spain (*n* = 3); UK (*n* = 4); and United States (*n* = 26). With respect to bullying victimization outcomes, these countries were as follows: Australia (*n* = 3); Canada (*n* = 7); Cyprus (*n* = 3); Finland (*n* = 6); Germany (*n* = 4); Greece (*n* = 2); Italy (*n* = 10); the Netherlands (*n* = 3); Norway (*n* = 7); Spain (*n* = 3); UK (*n* = 6); and United States (*n* = 28).

#### 7.4.3 Publication type and year

Overall, the majority of evaluations were published in peer‐reviewed journal articles, for both bullying perpetration (*n* = 67) and bullying victimization (*n* = 72) outcomes. Two evaluations were published in chapters of edited books and both reported effects of a program on both bullying victimization and perpetration. No evaluations identified were published as entire books. Moreover, 12 unpublished dissertations were identified that published evaluation data for bullying perpetration and bullying victimization outcomes. Data was also retrieved for both outcomes from three governmental reports. Four of the effect sizes included in the present report were estimated from data emailed to authors (M. M. T. and D. P. F.) in preparation of the previous Campbell report (i.e., Farrington & Ttofi, 2009).

We also categorized included evaluations according to whether they were included in the previous report (i.e., “2009” studies), or only included in the present report (i.e., “2016” studies). In relation to bullying perpetration outcomes, 37 studies were coded as 2009 studies and 53 studies were coded as 2016 studies. Similarly, more studies were coded as 2016 (*n* = 54) studies in comparison to 2009 (*n* = 39) studies for bullying victimization outcomes.

#### 7.4.4 Intervention program

We found that very few specific antibullying programs had been implemented and evaluated more than once using independent samples. Sixty‐five different school‐based bullying intervention and prevention programs were included in our meta‐analysis, but only eight were repeatedly evaluated. Moderator analysis with respect to the specific intervention program therefore, focused on programs that had been repeatedly evaluated.

In relation to reducing bullying perpetration outcomes the intervention programs thus included in our moderator analysis were: BPYS (*n* = 3; e.g., Menard & Grotpeter, 2014); fairplayer.manual (*n* = 2; e.g., Bull et al., 2009); KiVa (*n* = 6; Kärnä et al., 2011b); NoTrap! (*n* = 4; e.g., Menesini et al., 2012); Second Step (*n* = 3; e.g., Espelage et al., 2015); Steps to Respect (*n* = 2; e.g., Frey et al., 2005); ViSC (*n* = 5; e.g., Yanagida et al., 2019).

Similarly, these interventions were included in our moderator analysis in relation to bullying victimization outcomes with the exception of the fairplayer.manual program. This intervention was evaluated twice only in relation to bullying perpetration outcomes.

Additionally, multiple evaluations of the OBPP were included in our meta‐analysis. Overall, 12 independent evaluations of this intervention were included in our analysis in relation to bullying perpetration and victimization outcomes. These are included in our moderator analysis as a collective subgroup and also as further subgroups. Evaluations of the OBPP conducted in the United States (perpetration *n* = 6; victimization *n* = 7) and those conducted in Norway (perpetration *n* = 5; victimization *n* = 5) were included in the moderator analysis separately. There was one evaluation of the OBPP conducted in Malaysia is included in the overall category (*n* = 12).

#### 7.4.5 Unit of allocation/randomization

Systematic review findings showed that one consistent issue with included intervention programs was that the unit of allocation of participants, or clusters of participants, was different to the unit of analysis in most evaluations. Age cohort designs were omitted from this moderator analysis as the unit of allocation was largely unclear due to the logistics of this experimental design.

The majority of RCT and BA/EC evaluations assigned schools to experimental conditions (perpetration *n* = 44; victimization *n* = 47) yet the unit of analysis was individual students. A number of evaluations (perpetration *n* = 19; victimization *n* = 15) assigned classes to experimental conditions yet the unit of analysis was individual students. Less than 10 evaluations (perpetration *n* = 7; victimization *n* = 9) included assigned students to experimental and control conditions. One study randomly assigned districts to experimental conditions, and information was not available for five studies in relation to bullying perpetration outcomes and four studies in relation to bullying victimization.

#### 7.4.6 Conflict of interest

In the present report, 40 studies were categorized as high COI. A large number of studies (perpetration *n* = 36; victimization *n* = 39) were considered low COI, and 14 were categorized as possible COI. Information concerning COI was unavailable for 4 evaluations in relation to bullying perpetration outcomes.

#### 7.4.7 Program specificity

Overall, a small number (*n* = 11) of studies included in our analysis were coded as low on the program specificity variable. The vast majority of evaluations were considered highly specific (i.e., were mostly concerned with only bullying behavioral outcomes; *n* = 59). Additionally, 18 studies were categorized as medium in relation to specificity, where extra outcome variables were measured but these variables were related to bullying (e.g., school climate).

### 7.5 Risk of bias analysis

**Figure 2:**
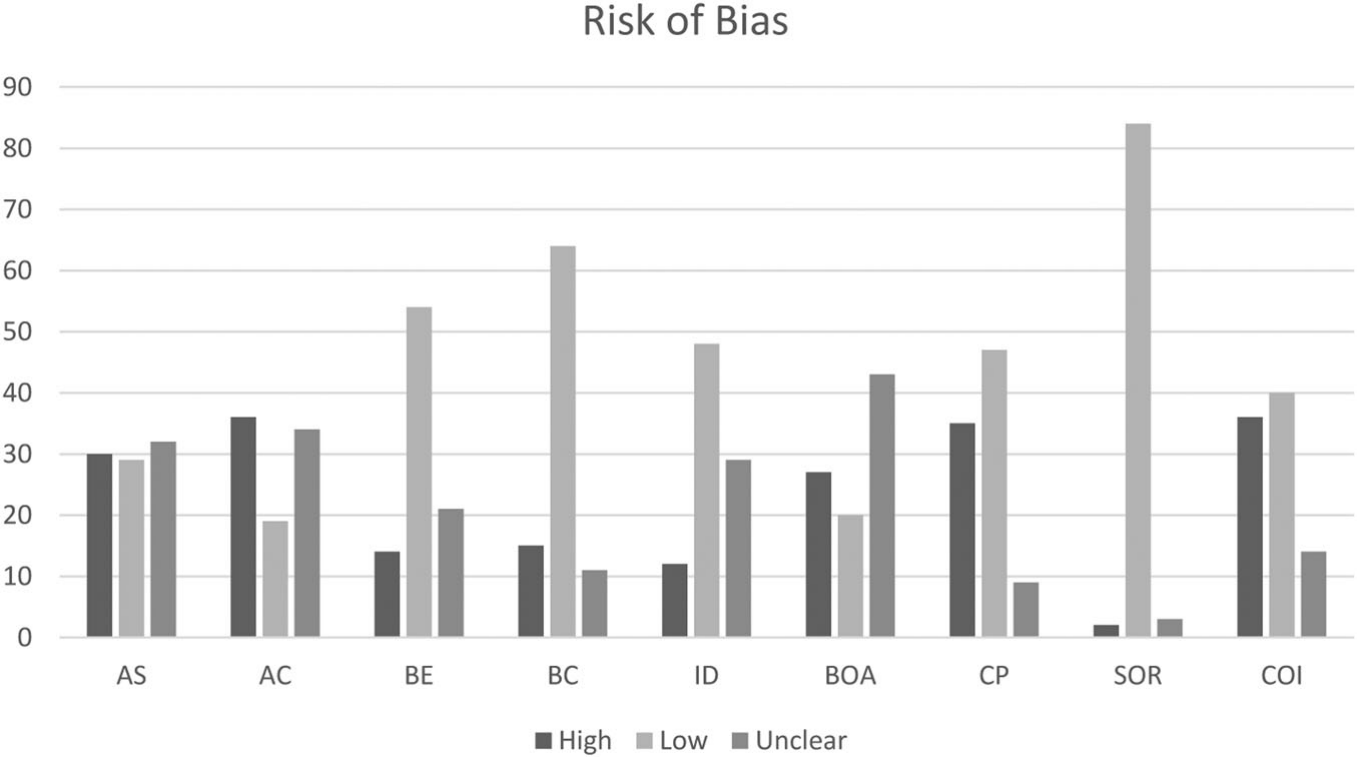
Risk of bias analysis results. AC, allocation concealment; AS, allocation sequence; BC, baseline equivalence on participant characteristics; BE, baseline equivalence on outcomes; BOA, blind outcome assessment; COI, conflict of interest; CP, contamination protection; ID, incomplete outcome data; SOR, selected outcome reporting

Figure 2 presents the results of the risk of bias analysis for each of the items on the EPOC tool and the additional items we included. The following section describes each of these categories in more detail, with examples of high‐ and low‐risk studies included. The main limitation in assessing risk of bias was the lack of information reported by primary studies. Thus, while the best effort was made to categorize each primary evaluation as being high or low risk, a large number of studies were recorded as “unclear” risk.

As seen in Figure 2, the fewest studies were considered unclear risk on CP and selected outcome reporting. Furthermore, a large number of studies were considered low risk on these items.

For the purpose of analysis, the categories high, unclear, and low risk were transformed into scores of 3, 2, and 0 respectively. A continuous “risk of bias” variable was then estimated as the sum total of scores on each of the EPOC items. As such, the lowest possible score a study could be given was zero and the maximum score was 24.

Descriptive statistical analysis identified that risk of bias scores ranged from 0 to 17, with a mean score of 9.62. Meta‐regression analysis was conducted to assess the relationship between risk of bias and effect sizes. The result of this analysis is included in Section 7 of this report. The following sections provide more detail about each of the risk categories.

#### 7.5.1 Allocation sequence

AS refers to the way in which participants, or clusters of participants, were assigned to experimental conditions. For example, low‐risk studies were those where a random number generator or another randomization software was used. In total, 30 studies were categorized as high risk on the AS item. Moreover, 29 studies were low risk and 32 were unclear risk.

#### 7.5.2 Allocation concealment

AC item refers to whether the method of allocation was concealed from participants or not. In total, 36 studies were categorized as high risk on the AC item. A further 19 studies were considered low risk, and 34 were unclear risk.

#### 7.5.3 Baseline equivalence: Outcome

Baseline equivalence refers to the comparability of experimental and control participants before the intervention has taken place. This item specifically refers to equivalence on relevant outcomes, in this case, school bullying perpetration and victimization. When experimental and control participants are not statistically significant at baseline then we can be more certain that any changes are a result of the intervention. Overall, 14 studies were categorized as high risk on the baseline equivalence on bullying outcomes item. A total of 54 studies were low risk and 21 were unclear risk.

#### 7.5.4 Baseline equivalence: Characteristics

Similarly, baseline equivalence on participant characteristics increases the chance that any change is a result of the intervention, and not a confounding variable such as differential participant characteristics at baseline. Overall, 15 studies were categorized as high risk on the baseline equivalence in participant characteristics item, 64 studies were low risk, and 11 were unclear risk.

#### 7.5.5 Incomplete outcome data

Included evaluations were required to incorporate pre‐ and post‐intervention measures of bullying (except if randomization was used). However, because of this, it is likely that there will be some attrition in primary studies. The incomplete outcome data item referred to the risk associated with differential attrition between experimental groups and/or ways in which attrition and missing cases were dealt with by primary studies. Twelve studies were categorized as high risk on the incomplete outcome data item. Additionally, 48 studies were low risk and 29 were unclear risk.

#### 7.5.6 Blind outcome assessment

This item assesses the risk associated with any bias which may arise if outcome measurements are not conducted blindly. In other words, if the individual, or individuals, who administer and collect the measurement instruments are aware of the experimental conditions of participants at the time of measurement. Overall, 27 studies were categorized as high risk on the BOA item. Twenty studies were low risk and 43 were unclear risk.

#### 7.5.7 Contamination protection

Risk of contamination occurs when there is a possibility that experimental and control participants may interact or encounter one another during the course of the evaluation. Thus, the effects of the intervention may “spill over” to control students and impact the results of the evaluation. In our analysis, 35 studies were categorized as high risk on the CP item, 47 studies were low risk, and 9 were unclear risk.

#### 7.5.8 Selective outcome reporting

SOR occurs when the outcomes reported in an evaluation study differ from the outcomes of interest proposed originally. For example, if a trial protocol proposed different outcomes than those actually reported in the publication of the trial results. Two studies were categorized as high risk on the SOR item. Eighty‐four studies were low risk, and three were unclear risk.

## 8 META‐ANALYSIS

After accounting for missing information, studies excluded because of their methodology (i.e., “other experimental‐control” designs), and studies with overlapping samples, a total of 41 studies were excluded from the meta‐analysis. Thus, a total of 100 studies were eligible for inclusion in our meta‐analysis. Table 8 outlines the raw data from these studies used to estimate effect sizes. The Comprehensive Meta‐Analysis (CMA) software was used to estimate all summary effect sizes in the present meta‐analysis.

**Table 8:** Raw data from included evaluations

Program	Evaluation	Bullying perpetration	Bullying victimization
*Randomized experiments*			
Australian Anti‐Bullying Intervention	Hunt (2007)	*Bullying alone:* EB: *M* = 1.30; *SD* = 0.60; n = 152 EA: *M* = 1.17; *SD* = 0.46; n = 111 CB: *M* = 1.30; *SD* = 0.66; n = 248 CA: *M* = 1.31; *SD* = 0.64; n = 207 *Bullying in a Group:* EB: *M* = 1.47; *SD* = 0.70; n = 152 EA: *M* = 1.39; *SD* = 0.72; n = 111 CB: *M* = 1.36; *SD* = 0.75; n = 248 CA: *M* = 1.41; *SD* = 0.76; n = 207	EB: *M* = 1.86; *SD* = 1.21; n = 152 EA: *M* = 1.53; *SD* = 1.12; n = 111 CB: *M* = 1.71; *SD* = 1.05; n = 248 CA: *M* = 1.52; *SD* = 1.08; n = 207
Behavioral Program for Bullying Boys	Meyer and Lesch (2000)	**Self‐report** *Treatment schools:* E_1_B: *M* = 104.16; *SD* = 26.24; n = 6 E_1_A_1_: *M* = 119. 50; *SD* = 16.57; n = 6 E_2_B: *M* = 82.00; *SD* = 28.50; n = 6 E_2_A_1_: *M* = 62.80; *SD* = 20.91; n = 6 E_3_B: *M* = 86.00; *SD* = 17.81; n = 6 E_3_A_1_: *M* = 75.50; *SD* = 21.51; n = 6 *Play/adult supervision control:* C_1_B: *M* = 88.60; *SD* = 34.17; n = 6 C_1_A_1:_ *M* = 86.16; *SD* = 33.09; n = 6 C_2_B: *M* = 73.30; *SD* = 13.36; n = 6 C_2_A_1_: *M* = 60.67; *SD* = 25.57; n = 6 C_3_B: *M* = 84.40; *SD* = 17.81; n = 6 C_3_A_1_: *M* = 102.8; *SD* = 18.63; n = 6 *No treatment/supervision control:* C_1_B: *M* = 75.16; *SD* = 34.09; n = 6 C_1_A_1_: *M* = 74.00; *SD* = 41.07; n = 6 C_2_B: *M* = 86.40; *SD* = 49.03; n = 6 C_2_A_1_: *M* = 54.20; *SD* = 13.92; n = 6 C_3_B: *M* = 93.60; *SD* = 21.83; n = 6 C_3_A_1_: *M* = 109.40; *SD* = 53.26; n = 6 **Peer‐report** *Treatment schools:* E_1_B: *M* = 62.20; *SD* = 40.89; n = 6 E_1_A_1_: *M* = 75.40; *SD* = 29.04; n = 6 E_1_A_2_: *M* = 63.60; *SD* = 43.60; n = 6 E_2_B: *M* = 40.83; *SD* = 25.70; n = 6 E_2_A_1_: *M* = 46.50; *SD* = 20.36; n = 6 E_2_A_2_: *M* = 46.50; *SD* = 24.63; n = 6 E_3_B: *M* = 66.00; *SD* = 46.67; n = 6 E_3_A_1_: *M* = 55.60; *SD* = 37.70; n = 6 E_3_A_2_: *M* = 42.00; *SD* = 45.17; n = 6 *Play/adult supervision control:* C_1_B: *M* = 77.30; *SD* = 44.52; n = 6 C_1_A_1:_ *M* = 59.30; *SD* = 18.12; n = 6 C_1_A_2_: *M* = 60.50; *SD* = 27.02; n = 6 C_2_B: *M* = 34.83; *SD* = 15.74; n = 6 C_2_A_1_: *M* = 28.50; *SD* = 16.10; n = 6 C_2_A_2_: *M* = 26.83; *SD* = 21.10; n = 6 C_3_B: *M* = 53.20; *SD* = 32.50; n = 6 C_3_A_1_: *M* = 35.60; *SD* = 29.08; n = 6 C_3_A_2_: *M* = 42.40; *SD* = 25.74; n = 6 *No treatment/supervision control:* C_1_B: *M* = 57.60; *SD* = 19.27; n = 6 C_1_A_1_: *M* = 60.50; *SD* = 21.95; n = 6 C_1_A_2_: *M* = 51.60; *SD* = 22.88; n = 6 C_2_B: *M* = 42.60; *SD* = 29.96; n = 6 C_2_A_1_: *M* = 41.40; *SD* = 27.39; n = 6 C_2_A_2_: *M* = 35.80; *SD* = 29.40; n = 6 C_3_B: *M* = 33.80; *SD* = 20.92; n = 6 C_3_A_1_: *M* = 42.60; *SD* = 25.35; n = 6 C_3_A_2_: *M* = 51.00; *SD* = 44.10; n = 6	
Bulli & Pape	Baldry and Farrington (2004)	*Younger:* EB: *M* = 1.69; *SD* = 2.15; n = 58 EA: *M* = 2.69; *SD* = 3.31; n = 26 CB: *M* = 1.54; *SD* = 2.20; n = 57 CA: *M* = 1.57; *SD* = 2.20; n = 72 *Older:* EB: *M* = 2.54; *SD* = 3.59; n = 63 EA: *M* = 2.31; *SD* = 3.07; n = 99 CB: *M* = 2.11; *SD* = 2.44; n = 46 CA: *M* = 3.39; *SD* = 3.99; n = 36	*Younger:* EB: *M* = 3.66; *SD* = 4.36; n = 59 EA: *M* = 2.24; *SD* = 3.50; n = 29 CB: *M* = 3.25; *SD* = 3.50; n = 56 CA: *M* = 1.85; *SD* = 2.62; n = 71 *Older:* EB: *M* = 3.64; *SD* = 4.89; n = 64 EA: *M* = 2.31; *SD* = 3.89; n = 99 CB: *M* = 1.84; *SD* = 2.35; n = 44 CA: *M* = 2.79; *SD* = 2.48; n = 38
CBT and CBT + media	McLaughlin (2009)	*E*_*1*_ *: CBT group* E_1_B: *M* = 13.79; *SD* = 4.15; n = 28 E_1_A: *M* = 13.32; *SD* =3.74; n = 28 *E*_*2*_ *: CBT + media* E_2_B: *M* = 11.08; *SD* = 1.63; n = 25 E_2_A: *M* = 11.68; *SD* = 2.58; n = 25 *C: Waitlist control* CB: *M* = 13.47; *SD* = 6.41; n = 15 CA: *M* = 13.13; *SD* = 5.45; n = 15	*E*_*1*_ *: CBT group* E_1_B: *M* = 14.64; *SD* = 5.44; n = 28 E_1_A: *M* = 13.50; *SD* = 4.07; n = 28 *E*_*2*_ *: CBT + media* E_2_B: *M* = 15.28; *SD* = 6.28; n = 25 E_2_A: *M* = 13.20; *SD* = 3.51; n = 25 *C: Waitlist Control* CB: *M* = 16.93; *SD* = 9.71; n = 15 CA: *M* = 16.67; *SD* = 9.36; n = 15
Chinese antibullying intervention	Ju et al. (2009)	‐	**Way to school:** *Victimization “At least once or twice”:* EB: 32%; n = 233 EA: 14%; n = 233 CB: 37%; n = 121 CA: 22%; n = 121 *Victimization “Often”:* EB: 10%; n = 233 EA: 5%; n = 233 CB: 11%; n = 121 CA: 5%; n = 121 **Way home from school:** *Victimization “At least once or twice”:* EB: 35%; n = 233 EA: 17%; n = 233 CB: 45%; n = 121 CA: 38%; n = 121 *Victimization “Often”:* EB: 11%; n = 233 EA: 8%; n = 233 CB: 10%; n = 121 CA: 9%; n = 121
Clinical prevention program	Tsiantis et al. (2013)	EB: n = 18; N = 331 EA: n = 8; N = 306 CB: n = 13; N = 335 CA: n = 11; N = 316	EB: n = 56; N = 331 EA: n = 25; N = 306 CB: n = 27; N = 335 CA: n = 21; N = 316
The Confident Kids Program	Berry and Hunt (2009)	‐	**Self‐report:** EB: *M* = 15.91; *SD* = 7.05; n = 22 EA: *M* = 7.54; *SD* = 6.44; n = 22 CB: 13.17; *SD* = 5.01; n = 24 CA: 12.58; *SD* = 5.98; n = 24 **Parent‐report:** EB: *M* = 13.00; *SD* = 7.30; n = 22* EA: *M* = 5.18; *SD* = 4.44; n = 22* CB: *M* = 8.37; *SD* = 4.64; n = 24* CA: *M* = 8.45; *SD* = 4.73; n = 24*
Cyberprogram 2.0	Garaigordobil and Martínez‐Valderrey (2015)	*Traditional bullying:* EB: *M* = 1.57; *SD* = 1.88; n = 93 EA: *M* = 0.70; *SD* = 1.09; n = 93 CB: *M* = 0.54; *SD* = 0.86; n = 83 CA: *M* = 0.93; *SD* = 1.39; n = 83	*Traditional Bullying:* EB: *M* = 0.75; *SD* = 1.10; n = 93 EA: *M* = 0.57; *SD* = 0.88; n = 93 CB: *M* = 0.55; *SD* = 1.01; n = 83 CA: *M* = 0.94; *SD* = 1.77; n = 83
DASI	Kyriakides, Creemers, Muijs, et al. (2014; Europe); Kyriakides, Creemers, and Papastylianou, et al. (2014; Cyprus and Greece)	*Awaiting information*	*Awaiting information*
Dutch antibullying program	Fekkes et al. (2006)	EB: 5.1%; n = 1101 EA1: 7.9%; n = 1098 EA2: 6.6%; n = 686 CB: 5.1%; n = 1110 CA1: 8.9%; n = 1108 CA2: 7.3%; n = 895	EB: 17.7%; n = 1106 EA1: 15.5%; n = 1104 EA2: 6.6%; n = 688 CB: 14.6%; n = 1115 CA1: 17.3%; n = 1112 CA2: 11.9%; n = 897
Emotional Literary program	Knowler and Frederickson (2013)	‐	*Low emotional literacy group:* EB: *M* = 16.24; *SD* = 13.32; n = 11 EA: *M* = 10.16; *SD* = 9.01; n = 11 CB: *M* = 19.04; *SD* = 10.84; n = 11 CA: *M* = 11.76; *SD* = 10.84; n = 11 *High Emotional Literacy Group:* EB: *M* = 9.92; *SD* = 8.82; n = 11 EA: *M* = 8.48; *SD* = 6.27; n = 11 CB: *M* = 17.03; *SD* = 10.32; n = 12 CA: *M* = 10.70; *SD* = 11.67; n = 12
Expect Respect	Rosenbluth et al. (2004)	EB: 10.6%; n = 929 EA: 17.0%; n = 741* CB: 11.2%; n = 834 CA: 17.8%; n = 665*	‐
Fairplayer.manual	Wölfer and Scheithauer (2014, p. 314)	EB: *M* = 1.20; *SD* = 0.33; n = 206 EA: *M* = 1.20; *SD* = 0.50; n = 198 CB: *M* = 1.25; *SD* = 0.53; n = 116 CA: *M* = 1.19; *SD* = 0.46; n = 113	‐
Fourth R	Cissner and Ayoub (2014)	**Baseline to T1:** *Reporting any perpetration:* EB: 55%; N = 570 EA: 63%; N = 570 CB: 59%; N = 175 CA: 61%; N = 175 *Reporting physical perpetration:* EB: 38%; N = 570 EA: 52%; N = 570 CB: 46%; N = 175 CA: 51%; N = 175 **Baseline to T2:** *Reporting any perpetration:* EB: 56%; N = EA: 58%; n = 263 CB: 59%; n = 248 CA: 63%; n = 248 *Reporting physical perpetration:* EB: 39%; n = 263 EA: 45%; n = 263 CB: 43%; n = 248 CA: 51%; n = 248	**Baseline to T1:** *Reporting any victimization:* EB: 66%; N = 570 EA: 76%; N = 570 CB: 74%; N = 175 CA: 75%; N = 175 *Reporting physical victimization:* EB: 41%; N = 570 EA: 55%; N = 570 CB: 46%; N = 175 CA: 54%; N = 175 **Baseline to T2:** *Reporting any victimization:* EB: 67%; n = 263 EA: 67%; n = 263 CB: 70%; n = 248 CA: 75%; n = 248 *Reporting physical victimization:* EB: 41%; n = 263 EA: 49%; n = 263 CB: 41%; n = 248 CA: 51%; n = 248
Friendly Schools Project	Cross et al. (2004)	EB: 13.0%; n = 135; N = 1038 EA1: 16.4%; n= 163; N = 992 CB: 15.1%; n = 139; N = 919 CA1: 15.2%; n = 133; N = 875	EB: 16.2%; n = 159; N = 982 EA1: 13.2%; n = 131; N = 990 EA2: 14.7%; n = 128; N = 869 CB: 15.7%; n = 135; N = 860 CA1: 13.9%; n = 122; N = 880 CA2: 14.6%; n = 116; N = 792
	Cross et al. (2011)	*Bullied others every few weeks+* EB: n = 27; N = 1037 EA1: n = 37; N = 973 EA2: n = 40; N = 841 EA3: n = 47; N = 675 CB: n = 28; N = 919 CA1: n = 25; N = 854 CA2: n = 41; N = 772 CA3: n = 40; N = 682 *Bullied others 1–2 a term:* EB: n = 108; N = 1037 EA1: n = 121; N = 973 EA2: n = 149; N = 841 EA3: n = 141; N = 675 CB: n = 111; N = 919 CA1: n = 105; N = 854 CA2: n = 141; N = 772 CA3: n = 144; N = 682	*Bullied every few weeks+* EB: n = 168; N = 1044 EA1: n = 131; N = 977 EA2: n = 126; N = 853 EA3: n = 87; N = 680 CB: n = 152; N = 918 CA1: n = 119; N = 857 CA2: n = 109; N = 771 CA3: n = 120; N = 679 *Bullied 1–2 a term:* EB: n = 262; N = 1044 EA1: n = 285; N = 977 EA2: n = 272; N = 853 EA3: n = 213; N = 680 CB: n = 220; N = 918 CA1: n = 303; N = 857 CA2: n = 275; N = 771 CA3: n = 206; N = 679
INCLUSIVE	Bonell et al. (2015)	‐	EB: *M* = 1.04; *SD* = 1.05; n = 508 EA: *M* = 1.02; *SD* = 0.96; n = 508 CB: *M* = 0.91; *SD* = 0.96; n = 509 CA: *M* = 0.89; *SD* = 0.94; n = 509
KiVa	Kärnä et al. (2011); Grades 4–6	*Self‐report* EB: *M* = 0.475; *SD* = 0.748; n = 4,201 EMid: *M* = 0.355; *SD* = 0.647; n = 4,201 EA: *M* = 0.273; *SD* = 0.565; n = 4,201 CB: *M* = 0.514; *SD* = 0.732; n = 3,965 CMid: *M* = 0.432; *SD* = 0.708; n = 3,965 CA: *M* = 0.348; *SD* = 0.597; n = 3,965 *Peer‐report:* EB: *M* = 0.069; *SD* = 0.119; n = 4,201 EMid: *M* = 0.060; *SD* = 0.109; n = 4,201 EA: *M* = 0.054; sd = 0.097; N = 4,201 CB: *M* = 0.071; *SD* = 0.120; n = 3,965 CMid: *M* = 0.070; *SD* = 0.120; n = 3,965 CA: *M* = 0.070; *SD* = 0.112; n = 3,965	*Self‐report:* EB: *M* = 0.741; *SD* = 1.071; n = 4,201 EMid: *M* = 0.738; *SD* = 1.068; n = 4,201 EA: *M* = 0.485; *SD* = 0.843; n = 4,201 CB: *M* = 0.782; *SD* = 1.064; n = 3,965 CMid: *M* = 0.829; *SD* = 1.101; n = 3,965 CA: *M* = 0.657; *SD* = 0.909; n = 3,965 *Peer‐report:* EB: *M* = 0.063; *SD* = 0.091; n = 4,201 EMid: *M* = 0.059; *SD* = 0.081; n = 4,201 EA: *M* = 0.049; *SD* = 0.075; n = 4,201 CB: *M* = 0.065; *SD* = 0.096; n = 3,965 CMid: *M* = 0.070; *SD* = 0.091; n = 3,965 CA: *M* = 0.065; *SD* = 0.081; n = 3,965
	Kärnä et al. (2013); Grades 2–3	*Self‐report:* EB: *M* = 0.07; *SD* = 0.26; n = 2,027 EMid: *M* = 0.04; *SD* = 0.20; n = 2,224 EA: *M* = 0.04; *SD* = 0.20; n = 2,019 CB: *M* = 0.07; *SD* = 0.25; n = 1,966 CMid: *M* = 0.05; *SD* = 0.23; n = 2,083 CA: *M* = 0.06; *SD* = 0.23; n = 2,018	*Self‐report:* EB: *M* = 0.22; *SD* = 0.42; n = 2,030 EMid: *M* = 0.13; *SD* = 0.34; n = 2,230 EA: *M* = 0.13; *SD* = 0.33; n = 2,020 CB: *M* = 0.23; *SD* = 0.42; n = 1,987 CMid: *M* = 0.16; *SD* = 0.37; n = 2,086 CA: *M* = 0.17; *SD* = 0.38; n = 2,018
	Kärnä et al. (2013); Grades 8–9	*Self‐report:* EB: *M* = 0.07; *SD* = 0.25; n = 5,690 EMid: *M* = 0.06; *SD* = 0.23; n = 5,530 EA: *M* = 0.05; *SD* = 0.23; n = 5,216 CB: *M* = 0.08; *SD* = 0.26; n = 4,327 CMid: *M* = 0.06; *SD* = 0.23; n = 4,358 CA: *M* = 0.07; *SD* = 0.25; n = 3,816 *Peer‐report:* EB: *M* = 0.05; *SD* = 0.10; n = 5,951 EMid: *M* = 0.05; *SD* = 0.09; n = 5,939 EA: *M* = 0.04; *SD* = 0.07; n = 5,885 CB: *M* = 0.05; *SD* = 0.10; n = 4,633 CMid: *M* = 0.05; *SD* = 0.09; n = 4,779 CA: *M* = 0.04; *SD* = 0.07; n = 4,488	*Self‐report:* EB: *M* = 0.09; *SD* = 0.29; n = 5,694 EMid: *M* = 0.06; *SD* = 0.24; n = 5,535 EA: *M* = 0.07; *SD* = 0.25; n = 5,252 CB: *M* = 0.10; *SD* = 0.30; n = 4,333 CMid: *M* = 0.08; *SD* = 0.27; n = 4,360 CA: *M* = 0.07; *SD* = 0.26; n = 3,847 *Peer‐report:* EB: *M* = 0.06; *SD* = 0.09; n = 5,951 EMid: *M* = 0.06; *SD* = 0.08; n = 5,940 EA: *M* = 0.05; *SD* = 0.07; n = 5,894 CB: *M* = 0.07; *SD* = 0.10; n = 4,633 CMid: *M* = 0.06; *SD* = 0.09; n = 4,779 CA: *M* = 0.05; *SD* = 0.07; n = 4,488
	Nocentini and Menesini (2016)	*Primary School:* EB: *M* = 0.059; *SD* = 0.086; n = 488 EA: *M* = 0.046; *SD* = 0.073; n = 442 CB: *M* = 0.064; *SD* = 0.090; n = 486 CA: *M* = 0.064; *SD* = 0.078; n = 462 *Middle school:* EB: *M* = 0.032; *SD* = 0.059; n = 529 EA: *M* = 0.029; *SD* = 0.053; n = 493 CB: *M* = 0.030; *SD* = 0.050; n = 516 CA: *M* = 0.041; *SD* = 0.063; n = 493	*Primary School:* EB: *M* = 0.134; *SD* = 0.122; n = 488 EA: *M* = 0.098; *SD* = 0.102; n = 443 CB: *M* = 0.138; *SD* = 0.122; n = 487 CA: *M* = 0.140; *SD* = 0.119; n = 462 *Middle School:* EB: *M* = 0.062; *SD* = 0.073; n = 533 EA: *M* = 0.057; *SD* = 0.073; n = 494 CB: *M* = 0.056; *SD* = 0.080; n = 516 CA: *M* = 0.075; *SD* = 0.086; n = 493
Media Heroes	Chaux et al. (2016)	EB: *M* = 0.31; *SD* = 0.47; n = 361 EA: *M* = 0.22; *SD* = 0.41; n = 361 CB: *M* = 0.34; *SD* = 0.45; n = 348 CA: *M* = 0.39; *SD* = 0.68; n = 348	EB: *M* = 0.39; *SD* = 0.54 n = 366 EA: *M* = 0.30; *SD* = 0.40; n = 366 CB: *M* = 0.41; *SD* = 0.48; n = 352 CA: *M* = 0.38; *SD* = 0.59; n = 352
The Positive Action Program	Li et al. (2011); Lewis et al. (2013)	OR = 1.69 (CI, 1.09–2.70)	
Preventure & Adventure	Topper (2011)	‐	*Preventure:* EB: *M* = 4.09; *SD* = 2.33; n = 167 EA1: *M* = 3.85; *SD* = 1.73; n = 167 EA2: *M* = 3.66; *SD* = 1.50; n = 167 EA3: *M* = 3.42; *SD* = 0.90; n = 167 CB: *M* = 4.57; *SD* = 1.85; n = 125 CA1: *M* = 4.03; *SD* = 1.86; n = 125 CA2: *M* = 3.88; *SD* = 1.46; n = 125 CA3: *M* = 3.64; *SD* = 1.17; n = 125 *Adventure:* EB: *M* = 5.04; SD = 2.62; n = 625 EA1: *M* = 4.65; *SD* = 2.43; n = 625 EA2: *M* = 4.38; *SD* = 2.21; n = 625 EA3: *M* = 4.16; *SD* = 1.91; n = 625 CB: *M* = 4.75; *SD* = 2.12; n = 464 CA1: *M* = 4.63; *SD* = 2.14; n = 464 CA2: *M* = 4.38; *SD* = 1.99; n = 464 CA3: *M* = 4.25; *SD* = 1.94; n = 464
Pro‐ACT+E	Sprober et al. (2006)	*Verbal bullying:* E_1_B: *M* = 22.95; *SD* = 5.64; n = 48* E_1_A1: *M* = 23.46; *SD* = 6.79; n = 48* E_1_A2: *M* = 21.73; *SD* = 4.70; n = 42* E_2_B: *M* = 22.94; *SD* = 6.27; n = 48* E_2_A1: *M* = 21.39; *SD* = 3.98; n = 48* E_2_A2: *M* = 21.38; *SD* = 3.57; n = 42* CB: *M* = 26.79; *SD* = 6.80; n = 48* CA1: *M* = 25.50; *SD* = 5.56; n = 48* CA2: *M* = 26.85; *SD* = 7.79; n = 42* *Physical bullying:* E_1_B: *M* = 26.78; *SD* = 2.37; n = 48* E_1_A1: *M* = 26.27; *SD* = 3.51; n = 48* E_1_A2: *M* = 26.67; *SD* = 3.53; n = 42* E_2_B: *M* = 26.72; *SD* = 4.05; n = 48* E_2_A1: *M* = 25.26; *SD* = 2.43; n = 48* E_2_A2: *M* = 25.68; *SD* = 2.17; n = 42* CB: *M* = 29.08; *SD* = 4.50; n = 48* CA1: *M* = 26.89; *SD* = 3.79; n = 48* CA2: *M* = 28.89; *SD* = 6.85; n = 42*	E_1_B: *M* = 20.02; *SD* = 5.75; n = 48* E_1_A1: *M* = 18.39; *SD* = 5.20; n = 48* E_1_A2: *M* = 17.71; *SD* = 4.70; n = 42* E_2_B: *M* = 19.76; *SD* = 4.26; n = 48* E_2_A1: *M* = 18.06; *SD* = 3.29; n = 48* E_2_A2: *M* = 17.84; *SD* = 3.46; n = 42* CB: *M* = 20.38; *SD* = 5.79; n = 48* CA1: *M* = 18.82; *SD* = 8.45; n = 48* CA2: *M* = 19.32; *SD* = 7.42; n = 42*
Project Ploughshares Puppets for Peace	Beran and Shapiro (2005)	EB: *M* = 10.41; *SD* = 4.27; n = 66 EA: *M* = 9.68; *SD* = 3.68; n = 66* CB: *M* = 8.91; *SD* = 3.49; n = 63 CA: *M* = 8.61; *SD* = 3.21; n = 63*	‐
REBE & ViSC	Trip et al. (2015)	*E*_*1*_ *: ViSC–REBE* E_1_B: *M* = 1.24; *SD* = 0.50; n = 228 E_1_Mid: *M* = 1.30; *SD* = 0.47; n = 201 E_1_A: *M* = 1.30; *SD* = 0.51; n = 183 *E*_*2*_ *: REBE–ViSC* E_2_B: *M* = 1.27; *SD* = 0.44; n = 326 E_2_Mid: *M* = 1.34; *SD* = 0.52; n = 291 E_2_A: *M* = 1.32; *SD* = 0.56; n = 211 *C: No treatment control* CB: *M* = 1.28; *SD* = 0.48; n = 249 CMid: *M* = 1.31; *SD* = 0.44; n = 230 CA: *M* = 1.39; *SD* = 0.48; n = 150	*E*_*1*_ *: ViSC–REBE* E_1_B: *M* = 1.41; *SD* = 0.60; n = 228 E_1_Mid: *M* = 1.48; *SD* = 0.65; n = 201 E_1_A: *M* = 1.45; *SD* = 0.66; n = 183 *E*_*2*_ *: REBE–ViSC* E_2_B: *M* = 1.43; *SD* = 0.63; n = 326 E_2_Mid: *M* = 1.47; *SD* = 0.67; n = 291 E_2_A: *M* = 1.45; *SD* = 0.71; n = 211 *C: No treatment control* CB: *M* = 1.48; *SD* = 0.61; n = 249 CMid: *M* = 1.43; *SD* = 0.60; n = 230 CA: *M* = 1.52; *SD* = 0.70; n = 150
The Resourceful Adolescent program	Stallard et al. (2013)	**High‐risk participants:** EB: 26.82%; n = 96; N = 358* EA1: 23.25%; n = 73; N = 314* EA2: 20.83%; n = 55; N = 264* *C*_*1*_ *: Usual PSHE:* C_1_B: 28.88%; n = 80; N = 277* C_1_A1: 30.08%; n = 77; N = 256* C_1_A2: 18.06%; n = 41; N = 227* *C*_*2*_ *: Attention control PSHE:* C_2_B: 33.71%; n = 118; N = 350* C_2_A1: 26.51%; n = 88; N = 332* C_2_A2: 20.50%; n = 57; N = 278* **All participants:** EB: 16.57%; n = 258; N = 1,557* EA1: 16.67%; n = 246; N = 1,476* EA2: 13.60%; n = 178; N = 1,309* *C*_*1*_ *: Usual PSHE:* C_1_B: 14.79%; n = 215; N = 1,454* C_1_A1: 15.58%; n = 223; N = 1,431* C_1_A2: 13.60%; n = 178; N = 1,309* *C*_*2*_ *: Attention control PSHE:* C_2_B: 20.74%; n = 312; N = 1,504* C_2_A1: 18.48%; n = 265; N = 1,434* C_2_A2: 16.28%; n = 209; N = 1,284*	‐
S.S. GRIN	DeRosier (2004); DeRosier and Marcus (2005)	EB: *M* = 0.09; *SD* = 1.08; n = 187 EA1: *M* = 0.15; *SD* = 1.22; n = 187 EA2: *M* = 0.15; *SD* = 1.32; n = 134 CB: *M* = 0.13; *SD* = 1.18; n = 194 CA1: *M* = 0.07; *SD* = 1.13; n = 194 CA2: *M* = 0.14; *SD* = 1.05; n = 140	EB: *M* = 0.31; *SD* = 1.10; n = 187 EA1: *M* = 0.38; *SD* = 1.16; n = 187 EA2: *M* = 0.31; *SD* = 1.12; n = 134 CB: *M* = 0.27; *SD* = 1.06; n = 194 CA1: *M* = 0.26; *SD* = 1.12; n = 194 CA2: *M* = 0.42; *SD* = 1.22; n = 140
Second Step	Espelage et al. (2013, 2015)	*Illinois:* EB: 24.6%; N = 1,061 EA: 29.8%; N = 1,061 CB: 28.2%; N = 968 CA: 36.2%; N = 968 *Kansas:* EB: 19.1%; N = 900 EA: 27.7%; N = 900 CB: 22.0%; N = 729 CA: 32.4%; N = 729	*Illinois:* EB: 50.0%; N = 1,061 EA: 50.7%; N = 1,061 CB: 52.2%; N = 968 CA: 56.3%; N = 968 *Kansas:* EB: 48.4%; N = 900 EA: 52.1%; N = 900 CB: 45.3%; N = 729 CA: 47.2%; N = 729
Second Step and Cultural Awareness Course	Polanin (2015)	EB: *M* = 0.154; *SD* = .0164; n = 28* EA1: *M* = 0.154; *SD* = 0.231; n = 28* EA2: *M* = 0.111; *SD* = 0.150; n = 28* EA3: *M* = 0.139; *SD* = 0.209; n = 28* CB: *M* = 0.223; *SD* = 0.277; n = 27* CA1: *M* = 0.275; *SD* = 0.335; n = 27* CA2: *M* = 0.293; *SD* = 0.345; n = 27* CA3: *M* = 0.269; *SD* = 0.331; n = 37*	EB: *M* = 0.792; *SD* = 0.974; n = 27* EA1: *M* = 0.739; *SD* = 0.872; n = 27* EA2: *M* = 0.568; *SD* = 0.690; n = 27* EA3: *M* = 0.456; *SD* = 0.604; n = 27* CB: *M* = 0.964; *SD* = 1.049; n = 27* CA1: *M* = 0.859; *SD* = 0.978; n = 27* CA2: *M* = 0.843; *SD* = 0.795; n = 27* CA3: *M* = 0.723; *SD* = 0.679; n = 27*
Short Video Intervention	Boulton and Flemington (1996)	EB: *M* = 9.00; *SD* = 2.10; n = 84 EA: *M* = 9.30; *SD* = 2.20; n = 84 CB: *M* = 14.80; *SD* = 5.30; n = 80 CA: *M* = 14.80; *SD* = 5.10; n = 80	
SPC + CAPSLE	Fonagy et al. (2009)	*E*_*1*_ *: CAPSLE* E_1_B: *M* = 100.4; *SD* = 9.72; n = 563 E_1_A: *M* = 98.9; *SD* = 9.02; n = 457 CB: *M* = 98.2; *SD* = 8.99; n = 360 CA: *M* = 99.3; *SD* = 8.18; n = 274	*E*_*1*_ *: CAPSLE* E_1_B: *M* = 100.64; *SD* = 9.49; n = 563 E_1_A: *M* = 99.0; *SD* = 9.63; n = 457 CB: *M* = 99.7; *SD* = 9.77; n = 360 CA: *M* = 99.8; *SD* = 9.20; n = 274
Steps to Respect	Brown et al. (2011) Students N = 2940 Teacher N = 1296	**Self‐report:** EB: *M* = 0.50; *SD* = 0.50; n = 1470* EA: *M* = 0.584; *SD* = 0.49; n = 1470* CB: *M* = 0.468; *SD* = 0.50; n = 1470* CA: *M* = 0.60; *SD* = 0.49; n = 1470* **Teacher‐report:** *Physical bullying:* EB: *M* = 0.21; *SD* = 0.41; n = 651* EA: *M* = 0.23; *SD* = 0.42; n = 651* CB: *M* = 0.17; *SD* = 0.378; n = 651* CA: *M* = 0.286; *SD* = 0.45; n = 651* *Nonphysical bullying:* EB: *M* = 0.42; *SD* = 0.49; n = 651* EA: *M* = 0.49; SD = 0.50; n = 651* CB: *M* = 0.40; *SD* = 0.489; n = 651* CA: *M* = 0.517; *SD* = 0.50; n = 651*	**Self‐report:** EB: *M* = 2.14; *SD* = 1.04; n = 1470* EA: *M* = 2.11; *SD* = 1.03; n = 1470* CB: *M* = 2.10; *SD* = 1.04; n = 1470* CA: *M* = 2.18; *SD* = 1.06; n = 1470*
	Frey et al. (2005)	*Direct bullying:* EB: *M* = 0.46; *SD* = 0.59; n = 563 EA: *M* = 0.48; *SD* = 0.62; n = 563 CB: *M* = 0.56; *SD* = 0.66; n = 563 CA: *M* = 0.62; *SD* = 0.71; n = 563 *Indirect bullying:* EB: *M* = 0.88; *SD* = 0.72; n = 563 EA: *M* = 0.90; *SD* = 0.74; n = 563 CB: *M* = 0.94; *SD* = 0.73; n = 563 CA: *M* = 0.96; *SD* = 0.83; n = 563	*Direct bullying:* EB: *M* = 1.01; *SD* = 0.79; n = 563 EA: *M* = 0.90; *SD* = 0.82; n = 563 CB: *M* = 1.07; *SD* = 0.82; n = 563 CA: *M* = 1.01; *SD* = 0.83; n = 563
SWPBIS	Waasdorp et al. (2012)	EB: *M* = 1.56; *SD* = 0.77; n = 6,614 EA: *M* = 1.78; *SD* = 0.86; n = 6,614 CB: *M* = 1.54; *SD* = 0.74; n = 5,124 CA: *M* = 1.87; *SD* = 0.83; n = 5,124	* * ‐
Take the LEAD	Domino (2011; 2013)	*Total:* EB: *M* = 1.15; *SD* = 1.47; n = 160 EA: *M* = 0.68; *SD* = 1.04; n = 160 CB: *M* = 1.39; *SD* = 1.73; n = 163 CA: *M* = 1.98; *SD* = 2.02; n = 163 *Boys:* EB: *M* = 1.53; *SD* = 1.78; n = 73 EA: *M* = 0.88; *SD* = 1.26; n = 73 CB: *M* = 1.84; *SD* = 2.05; n = 79 CA: *M* = 2.55; *SD* = 2.27; n = 79 *Girls:* EB: *M* = 0.84; *SD* = 1.07; n = 87 EA: *M* = 0.52; *SD* = 0.80; n = 87 CB: *M* = 0.95; *SD* = 1.22; n = 84 CA: *M* = 1.42; *SD* = 1.59; n = 84	*Total:* EB: *M* = 2.48; *SD* = 2.55; n = 160 EA: *M* = 1.26; *SD* = 1.80; n = 160 CB: *M* = 1.41; *SD* = 1.94; n = 163 CA: *M* = 2.25; *SD* = 2.40; n = 163 *Boys:* EB: *M* = 2.55; *SD* = 2.56; n = 73 EA: *M* = 1.35; *SD* = 1.79; n = 73 CB: *M* = 1.14; *SD* = 1.77; n = 79 CA: *M* = 1.91; *SD* = 2.22; n = 79 *Girls:* EB: *M* = 2.41; *SD* = 2.55; n = 87 EA: *M* = 1.18; *SD* = 1.81; n = 87 CB: *M* = 1.67; *SD* = 2.07; n = 84 CA: *M* = 2.57; *SD* = 2.53; n = 84
ViSC	Yanagida et al. (2019)	Latent *d* = 0.185 Rescaled SE^b^ = 0.162	Latent *d* = 0.725 Rescaled SE^b^ = 0.186
Youth‐led program	Connolly et al. (2015)	‐	EB: *M* = 0.32; *SD* = 0.42; n = 209 EA: *M* = 0.33; *SD* = 0.38; n = 209 CB: *M* = 0.37; *SD* = 0.42; n = 300 CA: *M* = 0.36; *SD* = 0.43; n = 300
Youth Matters	Jenson et al. (2010, 2013); Jenson and Dieterich (2007)	*Bullies:* EB: 16%; n = 61; N = 381 EA1: 11%; n = 39; N = 356* EA2: 13%; n = 32; N = 246 EA3: 12%; n = 34; N = 283* CB: 17%; n = 67; N = 394 CA1: 12%; n = 47; N = 392* CA2: 15%; n = 45; N = 300 CA3: 10%; n = 30; N = 289*	*Victims:* EB: 36%; n = 135; N = 375 EA1: 37%; n = 132; N = 356* EA2: 39%; n = 95; N = 244 EA3: 31%; n = 89; N = 283* CB: 31%; n = 122; N = 394 CA1: 37%; n = 143; N = 392* CA2: 40%; n = 117; N = 293 CA3: 39%; n = 113; N = 289*
Zippy's Friends	Holen et al. (2013)	* * *Teacher reports:* EB: *M* = 2.33; *SD* = 0.334; n = 685 EA: *M* = 2.52; *SD* = 0.364; n = 673 CB: *M* = 2.27; *SD* = 0.357; n = 625 CA: *M* = 2.30; *SD* = 0.461; n = 625	‐
*Before‐after, experimental‐control designs*			
Antibullying Pledge Scheme	Pryce and Frederickson (2013)	*Self‐report:* EB: *M* = 5.20; *SD* = 0.95; n = 182 EA: *M* = 5.33; *SD* = 1.18; n = 182 CB: *M* = 5.07; *SD* = 0.58; n = 135 CA: *M* = 4.90; *SD* = 0.46; n = 135 *Peer‐report:* EB: *M* = 0.15; *SD* = 0.09; n = 187 EA: *M* = 0.15; *SD* = 0.11; n = 187 CB: *M* = 0.14; *SD* = 0.04; n = 140 CA: *M* = 0.14; *SD* = 0.02; n = 140	*Self‐report:* EB: *M* = 7.80; *SD* = 1.23; n = 182 EA: *M* = 7.80; *SD* = 1.58; n = 182 CB: *M* = 7.23; *SD* = 0.80; n = 135 CA: *M* = 7.46; *SD* = 0.88; n = 135 *Peer‐report:* EB: *M* = 0.16; *SD* = 0.10; n = 187 EA: *M* = 0.17; *SD* = 0.13; n = 187 CB: *M* = 0.13; *SD* = 0.04; n = 140 CA: *M* = 0.13; *SD* = 0.02; n = 140
Be‐Prox	Alsaker and Valkanover (2001); Alsaker (2004)	EB: 41.1%; N = 150 EA: 40.1%; N = 152 CB: 31.7%; N = 161 CA: 33.5%; N = 165	* * EB: 57.7%; N = 150 EA: 49.3%; N = 152 CB: 32.9%; N = 161 CA: 52.1%; N = 164
Befriending Intervention program	Menesini et al. (2003)	EB: *M* = 2.24; *SD* = 4.89; n = 178 EA: *M* = 2.06; *SD* = 4.31; n = 178 CB: *M* = 2.04; *SD* = 3.72; n = 115 CA: *M* = 3.02; *SD* = 4.78; n = 115	* * EB: *M* = 3.53; *SD* = 6.19; n = 178 EA: *M* = 3.68; *SD* = 6.68; n = 178 CB: *M* = 3.06; *SD* = 5.54; n = 115 CA: *M* = 4.45 *SD* = 6.90; n = 115
Beyond the Hurt	Sutherland (2010)	*Males:* EB: *M* = 1.12; *SD* = 0.98; n = 133 EA: *M* = 1.00; *SD* = 1.36; n = 133 CB: *M* = 1.37; *SD* = 1.18; n = 144 CA: *M* = 1.00; *SD* = 1.30; n = 144 *Females:* EB: *M* = 0.92; *SD* = 1.00; n = 152* EA: *M* = 0.68; *SD* = 1.00; n = 152* CB: *M* = 1.12; *SD* = 1.17; n = 192* CA: *M* = 0.75; *SD* = 1.15; n = 192*	*Males:* EB: *M* = 1.21; *SD* = 0.94; n = 133 EA: *M* = 1.21; *SD* = 1.49; n = 133 CB: *M* = 0.93; *SD* = 0.87; n = 144 CA: *M* = 1.48; *SD* = 1.74; n = 144 *Females:* EB: *M* = 1.54; *SD* = 1.00; n = 152* EA: *M* = 1.06; *SD* = 1.18; n = 152* CB: *M* = 1.29; *SD* = 1.02; n = 192* CA: *M* = 1.17; *SD* = 1.33; n = 192*
The Bully Prevention Challenge Course Curriculum	Battey (2009)	‐	*Total victimization:* EB: *M* = 9.45; *SD* = 8.00; n = 89 EA1: *M* = 7.78; *SD* = 6.98; n = 65 EA2: *M* = 9.15; *SD* = 8.16; n = 50 CB: *M* = 11.39; *SD* = 7.60; n = 72 CA1: *M* = 8.64; *SD* = 7.80; n = 60 CA2: *M* = 9.24; *SD* = 7.71; n = 57 *Verbal victimization:* EB: *M* = 3.69; *SD* = 2.80; n = 89 EA1: *M* = 3.19; *SD* = 2.60; n = 65 EA2: *M* = 3.61; *SD* = 2.90; n = 50 CB: *M* = 4.17; *SD* = 2.40; n = 72 CA1: *M* = 3.33; *SD* = 2.70; n = 60 CA2: *M* = 3.91; *SD* = 2.90; n = 57 *Social victimization:* EB: *M* = 2.26; *SD* = 2.60; n = 89 EA1: *M* = 2.02; *SD* = 2.50; n = 65 EA2: *M* = 2.15; *SD* = 2.60; n = 50 CB: *M* = 2.91; *SD* = 2.70; n = 72 CA1: *M* = 2.53; *SD* = 2.90; n = 60 CA2: *M* = 2.52; *SD* = 2.80; n = 57 *Physical victimization:* EB: *M* = 1.40; *SD* = 2.20; n = 89 EA1: *M* = 1.00; *SD* = 1.70; n = 65 EA2: *M* = 1.46; *SD* = 2.00; n = 50 CB: *M* = 1.77; *SD* = 2.30; n = 72 CA1: *M* = 2.91; *SD* = 2.20; n = 60 CA2: *M* = 1.12; SD = 2.00; n = 57 *Attack on property:* EB: *M* = 1.99; *SD* = 2.20; n = 89 EA1: *M* = 1.45; *SD* = 2.00; n = 65 EA2: *M* = 1.98; *SD* = 2.30; n = 50 CB: *M* = 2.18; *SD* = 2.30; n = 72 CA1: *M* = 1.93; *SD* = 2.60; n = 60 CA2: *M* = 1.66; *SD* = 2.20; n = 57
Bully‐Proofing Your School	Beran et al. (2004)	‐	* * EB: *M* = 5.77; *SD* = 6.10; n = 25 EA: *M* = 5.36; *SD* = 5.50; n = 25 CB: *M* = 3.60; *SD* = 3.50; n = 77 CA: *M* = 3.41; *SD* = 3.40; n = 77
	Menard and Grotpeter (2014)	*Physical aggression:* EB: *M* = 7.12; *SD* = 2.80; n = 156 EA: *M* = 6.73; *SD* = 2.67; n = 713 CB: *M* = 7.34; *SD* = 2.64; n = 401 CA: *M* = 7.25; *SD* = 3.21; n = 1,665 *Relational aggression:* EB: *M* = 5.64; *SD* = 2.20; n = 156 EA: *M* = 5.43; *SD* = 2.94; n = 713 CB: *M* = 5.97; *SD* = 2.31; n = 401 CA: *M* = 5.73; *SD* = 2.38; n = 1,665	*Physical aggression:* EB: *M* = 7.57; *SD* = 2.94; n = 156 EA: *M* = 7.03; *SD* = 2.94; n = 713 CB: *M* = 7.42; *SD* = 3.15; n = 401 CA: *M* = 7.65; *SD* = 3.19; n = 1,665 *Relational aggression:* EB: *M* = 5.89; *SD* = 2.51; n = 156 EA: *M* = 5.51; *SD* = 2.63; n = 713 CB: *M* = 5.96; *SD* = 2.80; n = 401 CA: *M* = 5.90; *SD* = 2.69; n = 1,665
	Menard et al. (2008)^a^	**Elementary schools:** *Physical aggression:* B: r = ‐0.063; n = 708 A1: r = 0.044; n = 636 A2: r = 0.102; n = 708 A3: r = 0.116; n = 735 A4: r = 0.047; n = 710 *Relational aggression:* B: r = ‐0.103; n = 708 A1: r = ‐0.066; n = 636 A2: r = 0.080; n = 708 A3: r = 0.134; n = 735 A4: r = 0.052; n = 710 **Middle schools:** *Physical aggression:* B: r = 0.040; n = 280 A1: r = ‐0.128; n = 306 A2: r = 0.009; n = 339 A3: r = 0.080; n = 354 A4: r = 0.049; n = 348 *Relational aggression:* B: r = 0.019; n = 280 A1: r = ‐0.009; n = 306 A2: r = 0.092; n = 339 A3: r = 0.094; n = 354 A4: r = 0.092; n = 348	**Elementary schools:** *Physical aggression:* B: r = 0.005; n = 708 A1: r = ‐0.009; n = 636 A2: r = 0.052; n = 708 A3: r = 0.109; n = 735 A4: r = 0.101; n = 710 *Relational aggression:* B: r = ‐0.027; n = 708 A1: r = ‐0.028; n = 636 A2: r = 0.109; n = 708 A3: r = 0.051; n = 735 A4: r = 0.067; n = 710 **Middle schools:** *Physical aggression:* B: r = 0.060; n = 280 A1: r = 0.032; n = 306 A2: r = ‐0.022; n = 339 A3: r = ‐0.031; n = 354 A4: r = 0.040; n = 348 *Relational aggression:* B: r = 0.014; n = 280 A1: r = 0.036; n = 306 A2: r = ‐0.053; n = 339 A3: r = ‐0.027; n = 354 A4: r = ‐0.003; n = 348
Defeat Bullying	Herrick (2012)	‐	*E*_*1*_ *: Intervention* E_1_B: *M* = 2.00; *SD* = 4.35; n = 25 E_1_A1: *M* = 2.00; *SD* = 10.36; n = 25 E_1_A2: *M* = 0.83; *SD* = 2.74; n = 20 *E*_*2*_ *: Intervention + parent involvement* E_2_B: *M* = 15.20; *SD* = 4.35; n = 22 E_2_A1: *M* = 14.48; *SD* = 10.16; n = 21 E_2_A2: *M* = 12.85; *SD* = 12.34; n = 22 *C: Waitlist Control* CB: *M* = 2.50; *SD* = 2.04; n = 20 CA1: *M* = 0.00; *SD* = 0.00; n = 21 CA2: *M* = 0.00; *SD* = 0.00; n = 22
Drama program	Joronen et al. (2011)	*Bullied at least once:* EB: 39.7%; n = 31*; N = 78 EA: 33.8%; n = 26*; N = 78 CB: 30.2%; n = 17*; N = 56 CA: 28.6%; n = 16*; N = 56	*Victimized at least once:* EB: 58.8%; n = 46*; N = 78 EA: 38.1%; n = 30*; N = 78 CB: 37.7%; n = 21*; N = 56 CA: 39.3%; n = 22*; N = 56
Ecological Anti‐Bullying program	Rahey and Craig (2002)	*Junior children:* EB: *M* = 0.206; *SD* = 0.570; n = 125 EA: *M* = 0.254; *SD* = 0.779; n = 125 CB: *M* = 0.105; *SD* = 0.526; n = 67 CA: *M* = 0.224; *SD* = 0.487; n = 67 *Senior children:* EB: *M* = 0.425; *SD* = 0.895; n = 138 EA: *M* = 0.521; *SD* = 0.916; n =138 CB: *M* = 0.264; *SD* = 0.503; n = 176 CA: *M* = 0.391; *SD* = 0.714; n = 176	*Junior children:* EB: *M* = 1.220; *SD* = 1.34; n = 125 EA: *M* = 0.783; *SD* = 1.19; n = 125 CB: *M* = 1.090; *SD* = 1.29; n = 67 CA: *M* = 0.685; *SD* = 1.11; n = 67 *Senior children:* EB: *M* = 0.440; *SD* = 0.863; n = 138 EA: *M* = 0.890; *SD* = 1.29; n = 138 CB: *M* = 0.563; *SD* = 1.03; n = 176 CA: *M* = 0.685; *SD* = 1.11; n = 176
fairplayer.manual	Bull et al. (2009)	*E*_*1*_ *: Long‐term intervention:* E_1_B: 11.6%; n = 5; N = 43 E_1_A1: 4.7%; n = 2; N = 43 E_1_A2: 9.3%; n = 4; N = 43 *E*_*2*_ *: Short‐term intervention:* E_2_B: 10.0%; n = 4; N = 40 E_2_A1: 7.5%; n = 3; N = 40 E_2_A2: 12.5%; n = 5; N = 40 CB: 11.8%; n = 4; N = 34 CA1: 14.7%; n = 5; N = 34 CA2: 20.6%; n = 7; N = 34	*E*_*1*_ *: Long‐term intervention:* E_1_B: 11.6%; n = 5; N = 43 E_1_A1: 11.6%; n = 5; N = 43 E_1_A2: 11.6%; n = 5; N = 43 *E*_*2*_ *: Short‐term intervention:* E_2_B: 22.5%; n = 9; N = 40 E_2_A1: 15.0%; n = 6; N = 40 E_2_A2: 7.5%; n = 3; N = 40 CB: 8.8%; n = 3; N = 34 CA1: 14.7%; n = 5; N = 34 CA2: 20.6%; n = 7; N = 34
FearNot!	Sapouna et al. (2010)	EB: 11.3%; n = 48; N = 423 EA1: 10.8%; n = 47; N = 436 EA2: 11.1%; n = 48; N = 434 CB: 14.1%; n = 66; N = 469 CA1: 11.8%; n = 54; N = 457 CA2: 11.8%; n = 55; N = 465	EB: 25.7%; n = 109; N = 424 EA1: 20.8%; n = 91; N = 438 EA2: 20.5%; n = 88; N = 429 CB: 26.9%; n = 128; N = 475 CA1: 27.4%; n = 127; N = 463 CA2: 21.4%; n = 101; N = 471
Granada Anti‐Bullying Program	Martin et al. (2005)	EB: 44.00%; n = 25 EA: 28.00%; n = 25* CB: 20.83%; n = 24 CA: 25.00%; n = 24*	EB: 28.00%; n = 25 EA: 20.00%; n = 25* CB: 20.83%; n = 24 CA: 25.00%; n = 24*
Greek Anti‐Bullying Program	Andreou et al. (2007)	EB: *M* = 10.43; *SD* = 3.40; n = 248 EA1: *M* = 10.06; *SD* = 3.80; n = 246 EA2: *M* = 10.45; *SD* = 4.09; n = 234 CB: *M* = 9.87; *SD* = 3.65; n = 206 CA1: *M* = 10.85; *SD* = 3.72; n = 207 CA2: *M* = 10.81; *SD* = 3.94; n = 203	EB: *M* = 10.74; *SD* = 3.61; n = 248 EA1: *M* = 10.63; *SD* = 3.90; n = 248 EA2: *M* = 10.21; *SD* = 3.49; n = 235 CB: *M* = 10.62; *SD* = 3.78; n = 206 CA1: *M* = 11.17; *SD* = 3.68; n = 206 CA2: *M* = 11.03; *SD* = 3.89; n = 201
Lunch Buddy mentoring program	Elledge et al. (2010)	‐	**Self‐report:** *Lunch buddy:* EB: *M* = 1.92; *SD* = 0.38; n = 12 EA: *M* = 1.84; *SD* = 0.28; n = 11 *Different controls:* C_1_B: *M* = 1.93; *SD* = 0.33; n = 12 C_1_A: *M* = 1.72; *SD* = 0.46; n = 12 *Same controls:* C_2_B: *M* = 1.96; *SD* = 0.29; n = 12 C_2_A: *M* = 1.74; *SD* = 0.41; n = 12 **Peer‐report:** *Lunch buddy:* EB: *M* = 0.28; *SD* = 0.20; n = 12 EA: *M* = 0.13; *SD* = 0.12; n = 12 *Different controls:* C_1_B: *M* = 0.20; *SD* = 0.14; n = 12 C_1_A: *M* = 0.26; *SD* = 0.20; n = 12 *Same controls:* C_2_B: *M* = 0.26; *SD* = 0.25; n =12 C_2_A: *M* = 0.19; *SD* = 0.25; n = 12
NoTrap!	Menesini et al. (2012); Study 1	*E*_*1*_ *: Awareness* E_1_B: *M* = 2.596; *SD* = 0.332; n = 124 E_1_A: *M* = 2.618; *SD* = 0.275; n = 124 *E*_*2*_ *: Peer educators* E_2_B: *M* = 2.573; *SD* = 0.323; n = 61 E_2_A: *M* = 2.551; *SD* = 0.276; n = 60 CB: *M* = 2.633; *SD* = 0.296; n = 45 CA: *M* = 2.557; *SD* = 0.194; n = 45	*E*_*1*_ *: Awareness* E_1_B: *M* = 2.478; *SD* = 0.528; n = 127 E_1_A: *M* = 2.568; *SD* = 0.251; n = 127 *E*_*2*_ *: Peer educators* E_2_B: *M* = 2.582; *SD* = 0.213; n = 60 E_2_A: *M* = 2.556; *SD* = 0.237; n = 60 CB: *M* = 2.615; *SD* = 0.194; n = 45 CA: *M* = 2.589; *SD* = 0.213; n = 45
	Menesini et al. (2012); Study 2; and Palladino et al. (2012)	*Overall:* EB: *M* = 1.20; *SD* = 0.27; n = 231 EA: *M* = 1.17; *SD* = 0.28; n = 231 CB: *M* = 1.24; *SD* = 0.33; n = 144 CA: *M* = 1.29; *SD* = 0.36; n = 144 *E*_*1*_ *: Peer educators* E_1_B: *M* = 1.23; *SD* = 0.31; n = 42 E_1_A: *M* = 1.19; *SD* = 0.38; n = 42 *E*_*2*_ *: Other experimental* E_2_B: *M* = 1.19; *SD* = 0.26; n = 189* E_2_A: *M* = 1.17; *SD* = 0.26; n = 189*	*Overall:* EB: *M* = 1.22; *SD* = 0.31; n = 231 EA: *M* = 1.17; *SD* = 0.26; n = 231 CB: *M* = 1.24; *SD* = 0.27; n = 144 CA: *M* = 1.28; *SD* = 0.29; n = 144 *E*_*1*_ *: Peer educators* E_1_B: *M* = 1.24; *SD* = 0.38; n = 42 E_2_B: *M* = 1.20; *SD* = 0.31; n = 42 *E*_*2*_ *: Other experimental* E_2_B: *M* = 1.22; *SD* = 0.28; n = 189* E_2_A: *M* = 1.17; *SD* = 0.25; n = 189*
	Palladino et al. (2016); Trial 1; 2011/2012	** ** EB: *M* = 0.117; *SD* = 0.13; n = 387 EMid: *M* = 0.113; *SD* = 0.15; n = 368 EA1: *M* = 0.083; *SD* = 0.11; n = 330 EA2: *M* = 0.060; *SD* = 0.07; n = 218 CB: *M* = 0.105; *SD* = 0.11; n = 131 CMid: *M* = 0.122; *SD* = 0.12; n = 138 CA1: *M* = 0.111; *SD* = 0.14; n = 110 CA2: *M* = 0.081; *SD* = 0.11; n = 76	** ** EB: *M* = 0.109; *SD* = 0.11; n = 389 EMid: *M* = 0.091; *SD* = 0.11; n = 372 EA1: *M* = 0.059; *SD* = 0.09; n = 338 EA2: *M* = 0.042; *SD* = 0.05; n = 224 CB: *M* = 0.093; *SD* = 0.10; n = 130 CMid: *M* = 0.106; *SD* = 0.13; n = 141 CA1: *M* = 0.090; *SD* = 0.12; n = 112 CA2: *M* = 0.060; *SD* = 0.08; n = 74
	Palladino et al. (2016); Trial 2; 2012/2013	*Males:* EB: *M* = 0.110; *SD* = 0.08; n = 67 EA: *M* = 0.068; *SD* = 0.06; n = 67 CB: *M* = 0.106; *SD* = 0.08; n = 173 CA: *M* = 0.130; *SD* = 0.11; n = 173 *Females:* EB: *M* = 0.084; *SD* = 0.08; n = 167 EA: *M* = 0.062; *SD* = 0.06; n = 167 CB: *M* = 0.097; *SD* = 0.08; n = 54 CA: *M* = 0.079; SD = 0.07; n = 54	*Males:* EB: *M* = 0.110; *SD* = 0.09; n = 67 EA: *M* = 0.063; *SD* = 0.05; n = 67 CB: *M* = 0.099; *SD* = 0.08; n = 173 CA: *M* = 0.095; *SD* = 0.10; n = 173 *Females:* EB: *M* = 0.094; *SD* = 0.08; n = 167 EA: *M* = 0.068; *SD* = 0.06; n = 167 CB: *M* = 0.098; *SD* = 0.07; n = 54 CA: *M* = 0.102; *SD* = 0.09; n = 54
OBPP	Bauer et al. (2007)	‐	*Physical victimization:* EB: 13.8%; N = 4531 EA: 14.6%; N = 4419 CB: 16.3%; N = 1373 CA: 17.5%; N = 1448 *Relational victimization:* EB: 24.8%; N = 4607 EA: 24.7%; N = 4480 CB: 30.4%; N = 1408 CA: 30.2%; N = 1456
	“Bergen 2”	EB: 5.60%; N = 1278 EA: 4.40%; N = 1296 CB: 4.10%; N = 1111 CA: 5.60%; N = 1168	* * EB: 12.70%; N = 1297 EA: 9.70%; N = 1320 CB: 10.60%; N = 1117 CA: 11.10%; N = 1179
	Finn (2009)	*Group:* EB: *M* = 1.13; *SD* = 0.24; n = 435 EA: *M* = 1.12; *SD* = 0.27; n = 437 CB: *M* = 1.13; *SD* = 0.21; n = 372 CA: *M* = 1.14; *SD* = 0.23; n = 360 *Girls:* EB: *M* = 1.14; *SD* = 0.22; n = 207 EA: *M* = 1.11; *SD* = 0.26; n = 216 CB: *M* = 1.12; *SD* = 0.18; n = 189 CA: *M* = 1.12; *SD* = 0.20; n = 182 *Boys:* EB: *M* = 1.13; *SD* = 0.26; n = 228 EA: *M* = 1.13; *SD* = 0.27; n = 221 CB: *M* = 1.14; *SD* = 0.23; n = 183 CA: *M* = 1.16; *SD* = 0.26; n = 178	*Group:* EB: *M* = 1.56; *SD* = 0.60; n = 436 EA: *M* = 1.52; *SD* = 0.60; n = 440 CB: *M* = 1.54; *SD* = 0.57; n = 379 CA: *M* = 1.51; *SD* = 0.58; n = 360 *Girls:* EB: *M* = 1.62; *SD* = 0.59; n = 207 EA: *M* = 1.50; *SD* = 0.55; n = 216 CB: *M* = 1.55; *SD* = 0.58; n = 193 CA: *M* = 1.47; *SD* = 0.52; n = 182 *Boys:* EB: *M* = 1.50; *SD* = 0.60; n = 229 EA: *M* = 1.54; *SD* = 0.65; n = 224 CB: *M* = 1.53; *SD* = 0.57; n = 186 CA: *M* = 1.54; *SD* = 0.63; n = 178
	Losey (2009)	EB: *M* = 1.21; *SD* = 0.38; n = 237 EA: *M* = 1.21; *SD* = 0.49; n = 237 CB: *M* = 1.17; *SD* = 0.28; n = 416 CA: *M* = 1.15; *SD* = 0.30; n = 416	EB: *M* = 1.32; *SD* = 0.50; n = 235 EA: *M* = 1.33; *SD* = 0.60; n = 235 CB: *M* = 1.29; *SD* = 0.43; n = 420 CA: *M* = 1.25; *SD* = 0.46; n = 420
	Melton et al. (1998)	EB: 24.00%; N = 3904 EA: 20.00%; N = 3827 CB: 19.00%; N = 2485 CA: 22.00%; N = 2436	EB: 25.00%; N = 3904 EA: 19.00%; N = 3827 CB: 24.00%; N = 2485 CA: 19.00%; N = 2436
	Yaakub et al. (2013)	*Verbal bullying* EB: *M* = 4.14; *SD* = 3.40; n = 1877 EA: *M* = 4.66; *SD* = 3.60; n = 1878 CB: *M* = 3.76; *SD* = 3.33; n = 1934 CA: *M* = 4.73; *SD* = 3.25; n = 1938 *Physical bullying* EB: *M* = 0.86; *SD* = 1.89; n = 1875 EA: *M* = 1.14; *SD* = 2.29; n = 1871 CB: *M* = 0.99; *SD* = 2.01; n = 1935 CA: *M* = 1.03; *SD* = 2.00; n = 1934 *Relational bullying* EB: *M* = 0.51; *SD* = 0.91; n = 1878 EA: *M* = 0.53; *SD* = 0.98; n = 1878 CB: *M* = 0.45; *SD* = 0.87; n = 1935 CA: *M* = 0.58; *SD* = 0.94; n = 1937	
Progetto Pontassieve	Ciucci and Smorti (1998)	EB: 46.7%; N = 167 EA: 49.7%; N = 169 CB: 43.9%; N = 140 CA: 51.4%; N = 141	* * EB: 44.9%; N = 167 EA: 50.3%; N = 169 CB: 37.4%; N = 140 CA: 47.4%; N = 141
Restorative Whole School Approach	Wong et al. (2011)	**Bullying overall:** *E*_*1*_ *: RWsA* E_1_B: *M* = 1.40; *SD* = 0.39; n = 353 E_1_A: *M* = 1.33; *SD* = 0.32; n = 361 *E*_*2*_ *: Partial RWsA* E_2_B: *M* = 1.45; *SD* = 0.42; n = 550 E_2_A: *M* = 1.40; *SD* = 0.38; n = 584 *C: Non RWsA* CB: *M* = 1.48; *SD* = 0.49; n = 186 CA: *M* = 1.59; *SD* = 0.54; n = 206 **Physical bullying:** *E*_*1*_ *: RWsA* E_1_B: *M* = 1.32; *SD* = 0.46; n = 353 E_1_A: *M* = 1.22; *SD* = 0.38; n = 361 *E*_*2*_ *: Partial RWsA* E_2_B: *M* = 1.38; *SD* = 0.52; n = 550 E_2_A: *M* = 1.38; *SD* = 0.51; n = 584 *C: Non RWsA* CB: *M* = 1.47; *SD* = 0.63; n = 186 CA: *M* = 1.59; *SD* = 0.71; n = 206 **Verbal bullying:** *E*_*1*_ *: RWsA* E_1_B: *M* = 1.70; *SD* = 0.62; n = 353 E_1_A: *M* = 1.65; *SD* = 0.55; n = 361 *E*_*2*_ *: Partial RWsA* E_2_B: *M* = 1.74; *SD* = 0.64; n = 550 E_2_A: *M* = 1.72; *SD* = 0.57; n = 584 *C: Non RWsA* CB: *M* = 1.76; *SD* = 0.67; n = 186 CA: *M* = 1.94; *SD* = 0.75; n = 206 **Exclusion bullying:** *E*_*1*_ *: RWsA* E_1_B: *M* = 1.32; *SD* = 0.48; n = 353 E_1_A: *M* = 1.19; *SD* = 0.37; n = 361 *E*_*2*_ *: Partial RWsA* E_2_B: *M* = 1.38; *SD* = 0.52; n = 550 E_2_A: *M* = 1.25; *SD* = 0.44; n = 584 *C: Non RWsA* CB: *M* = 1.42; *SD* = 0.59; n = 186 CA: *M* = 1.14; *SD* = 0.60; n = 206 **Extortion bullying:** *E*_*1*_ *: RWsA* E_1_B: *M* = 1.28; *SD* = 0.42; n = 353 E_1_A: *M* = 1.25; *SD* = 0.37; n = 361 *E*_*2*_ *: Partial RWsA* E_2_B: *M* = 1.31; *SD* = 0.47; n = 550 E_2_A: *M* = 1.28; *SD* = 0.42; n = 584 *C: Non RWsA* CB: *M* = 1.34; *SD* = 0.51; n = 186 CA: *M* = 1.46; *SD* = 0.62; n = 206	‐
School‐bus antibullying program	Krueger (2010)	*Direct observations:* EB: *M* = 2.091; *SD* = 2.505; n = 22 EA: *M* = 0.818; *SD* = 1.053; n = 22 CB: *M* = 1.92; *SD* = 2.253; n = 22 CA: *M* = 1.80; *SD* = 2.739; n = 25	‐
SET program	Kimber (2008)	‐	*Grades 4–9:* EB: *M* = 1.23; *SD* = 0.49; n = 352 EA: *M* = 1.20; *SD* = 0.44; n = 352 CB: *M* = 1.18; *SD* = 0.40; n = 110 CA: *M* = 1.32; *SD* = 0.72; n = 110
Short Intensive Intervention in Czechoslovakia	Rican et al. (1996)	EB: 19.0%; N = 100 EA: 7.1%; N = 98 CB: 13.3%; N = 98 CA: 11.2%; N = 98	EB: 18.0%; N = 100 EA: 7.1%; N = 98 CB: 16.3%; N = 98 CA: 14.3%; N = 98
Social Skills Training (SST)	Fox and Boulton (2003)	‐	EB: *M* = 29.47; *SD* = 8.16; n = 15 EA: *M* = 34.29; *SD* = 16.01; n = 15 CB: *M* = 31.54; *SD* = 18.93; n = 13 CA: *M* = 33.56; *SD* = 20.15; n = 13
Stare bene a scuola	Gini et al. (2003)	EB: 11.1%; N = 63 EA: 17.5%; N = 63 CB: 19.1%; N = 47 CA: 23.4%; N = 47	EB: 36.5%; N = 63 EA: 41.3%; N = 63 CB: 51.1%; N = 47 CA: 34.0%; N = 47
Start: Strong	Williams et al. (2015)	‐	EB: 23%; N = 717 EA: 28%; N = 717 CB: 23%; N = 800 CA: 34%; N = 800
Strengths in Motion	Rawana et al. (2011)	EB: *M* = 2.69; *SD* = 1.20; n = 50 EA1: *M* = 2.87; *SD* = 1.24; n = 47* EA2: *M* = 2.50; *SD* = 0.98; n = 44* CB: *M* = 2.33; *SD* = 0.65; n = 53 CA1: *M* = 2.21; *SD* = 0.54; n = 50* CA2: *M* = 2.40; *SD* = 0.91; n = 46*	EB: *M* = 3.56; *SD* = 1.90; n = 50 EA1: *M* = 3.13; *SD* = 1.47; n = 47* EA2: *M* = 2.85; *SD* = 1.40; n = 44* CB: *M* = 3.28; *SD* = 1.60; n = 53 CA1: *M* = 3.22; *SD* = 1.87; n = 50* CA2: *M* = 2.90; *SD* = 1.40; n = 46*
Transtheoretical‐based tailored antibullying program	Evers et al. (2007)	*Middle school:* EB: 75.9%; N = 266 EA: 61.7%; N = 266 CB: 78.1%; N = 483 CA: 73.7%; N = 483 *High School:* EB: 67.6%; N = 531 EA: 49.2%; N = 531 CB: 71.5%; N = 309 CA: 67.0%; N = 309	*Middle school:* EB: 82.0%; N = 266 EA: 60.2%; N = 266 CB: 80.3%; N = 483 CA: 75.4%; N = 483 *High School:* EB: 68.4%; N = 531 EA: 50.7%; N = 531 CB: 75.4%; N = 309 CA: 68.6%; N = 309
ViSC	Gollwitzer et al. (2006)	EB: *M* = 1.56; *SD* = 0.51; n = 89 EA1: *M* = 1.58; *SD* = 0.63; n = 89* EA2: *M* = 1.46; *SD* = 0.45; n = 89* CB: *M* = 1.54; *SD* = 0.53; n = 60 CA1: *M* = 1.55; *SD* = 0.53; n = 60* CA2: *M* = 1.57; *SD* = 0.65; n = 60*	EB: *M* = 1.64; *SD* = 0.65; n = 89 EA1: *M* = 1.51; *SD* = 0.60; n = 89* EA2: *M* = 1.48; *SD* = 0.55; n = 89* CB: *M* = 1.63; *SD* = 0.49; n = 60 CA1: *M* = 1.62; *SD* = 0.60; n = 60* CA2: *M* = 1.56; *SD* = 0.60; n = 60*
*Age cohort designs*			
Donegal antibullying program	O'Moore and Milton (2004)	*Grade 4:* B: 10.49%; N = 181 A: 5.24%; N = 248	*Grade 4:* B: 19.23%; N = 182 A: 10.67%; N = 253
Finnish antibullying program	Salmivalli et al. (2005)	*Grade 4:* B: *M* = 0.15; *SD* = 0.36; n = 389 Low: *M* = 0.08; *SD* = 0.26; n = 247 High: *M* = 0.03; *SD* = 0.18; n = 125 *Grade 5:* B: *M* = 0.11; *SD* = 0.32; n = 417 Low: *M* = 0.12; *SD* = 0.32; n = 258 High: *M* = 0.07; *SD* = 0.25; n = 131	*Grade 4:* B: *M* = 0.14; *SD* = 0.34; n = 389 Low: *M* = 0.10; *SD* = 0.29; n = 247 High: *M* = 0.06; *SD* = 0.24; n = 125 *Grade 5:* B: *M* = 0.13; *SD* = 0.33; n = 417 Low: *M* = 0.11; *SD* = 0.32; n = 258 High: *M* = 0.07; *SD* = 0.26; n = 131
OBPP	Olweus/Bergen 1	*Grades 5–7:* B: 7.28%; N = 1689 A: 5.02%; N = 1663 *Grades 6–7:* B: 7.35%; N = 1294 A: 3.60%; N = 1103	*Grades 5–7:* B: 9.98%; N = 1874 A: 3.78%; N = 1691 *Grades 6–7:* B: 9.92%; N = 1297 A: 3.55%; N = 1115
	Olweus/New National	*Grades 5–7:* B: 5.7%; N = 8370 A1: 3.6%; N = 8295 *Grades 6–7:* B: 5.1%; N = 8222 A2: 2.6%; N = 8473	*Grades 5–7:* B: 15.2%; N = 8387 A1: 10.2%; N = 8299 *Grades 6–7:* B: 13.2%; N = 8238 A2: 8.7%; N = 8483
	Olweus/Olso 1	*Grades 5–7:* B: 6.4%; N = 874 A: 3.1%; N = 983	*Grades 5–7:* B: 14.4%; N = 882 A: 8.5%; N = 986
	Olweus/Olso 2	*Grades 4–7:* B: 5.5%; N = 2682 A1: 2.8%; N = 3077 A2: 2.3%; N = 3022 A3: 2.8%; N = 2535 A4: 2.7%; N = 2834 *Grades 8–10:* B: 6.2%; N = 1445 A1: 5.7%; N = 1449 A2: 4.1%; N = 1526	*Grades 4–7:* B: 14%; N = 2695 A1: 9.8%; N = 3077 A2: 8.8%; N = 3026 A3: 8%; N = 2538 A4: 8.4%; N = 2967 *Grades 8–10:* B: 7.1%; N = 1452 A1: 6.8%; N = 1462 A2: 5.2%; N = 1532
OBPP: Chula Vista	Pagliocca et al. (2007)	*Grades 3–6:* B: 27.86%; N = 1177 A1: 22.88%; N = 1088 A2: 24.33%; N = 1126	*Grades 3–6:* B: 12.91%; N = 1177 A1: 10.84%; N = 1088 A2: 10.39%; N = 1126
Respect	Ertesvag and Vaaland (2007)	*Grade 5:* B: *M* = 0.29; *SD* = 0.32; n = 118 A1: *M* = 0.31; *SD* = 0.43; n = 126 A2: *M* = 0.21; *SD* = 0.33; n = 151 A3: *M* = 0.17; *SD* = 0.38; n = 143 *Grade 6:* B: *M* = 0.36; *SD* = 0.38; n = 152 A1: *M* = 0.28; *SD* = 0.43; n = 129 A2: *M* = 0.17; *SD* = 0.25; n = 130 A3: *M* = 0.21; *SD* = 0.30; n = 140 *Grade 7:* B: *M* = 0.31; *SD* = 0.32; n = 147 A1: *M* = 0.32; *SD* = 0.39; n = 160 A2: *M* = 0.30; *SD* = 0.40; n = 134 A3: *M* = 0.15; *SD* = 0.28; n = 140 *Grade 8:* B: *M* = 0.32; *SD* = 0.49; n = 123 A1: *M* = 0.25; *SD* = 0.33; n = 128 A2: *M* = 0.41; *SD* = 0.60; n = 112 A3: *M* = 0.25; *SD* = 0.49; n = 123 *Grade 9:* B: *M* = 0.34; *SD* = 0.55; n = 95 A1: *M* = 0.32; *SD* = 0.48; n = 128 A2: *M* = 0.35; *SD* = 0.59; n = 112 A3: *M* = 0.33; *SD* = 0.49; n = 122 *Grade 10:* B: *M* = 0.35; *SD* = 0.49; n = 112 A1: *M* = 0.41; *SD* = 0.55; n = 99 A2: *M* = 0.38; *SD* = 0.60; n = 149 A3: *M* = 0.31; *SD* = 0.56; n = 124	*Grade 5:* B: *M* = 0.54; *SD* = 0.49; n = 118 A1: *M* = 0.53; *SD* = 0.53; n = 126 A2: *M* = 0.43; *SD* = 0.48; n = 151 A3: *M* = 0.44; *SD* = 0.54; n = 143 *Grade 6:* B: *M* = 0.46; *SD* = 0.46; n = 152 A1: *M* = 0.50; *SD* = 0.57; n = 129 A2: *M* = 0.38; *SD* = 0.47; n = 130 A3: *M* = 0.39; *SD* = 0.46; n = 140 *Grade 7:* B: *M* = 0.44; *SD* = 0.51; n = 147 A1: *M* = 0.39; *SD* = 0.52; n = 160 A2: *M* = 0.44; *SD* = 0.52; n = 134 A3: *M* = 0.39; *SD* = 0.46; n = 140 *Grade 8:* B: *M* = 0.30; *SD* = 0.57; n = 123 A1: *M* = 0.21; *SD* = 0.34; n = 128 A2: *M* = 0.57; *SD* = 0.74; n = 112 A3: *M* = 0.32; *SD* = 0.40; n = 123 *Grade 9:* B: *M* = 0.26; *SD* = 0.39; n = 95 A1: *M* = 0.26; *SD* = 0.46; n = 128 A2: *M* = 0.36; *SD* = 0.55; n = 112 A3: *M* = 0.44; *SD* = 0.55; n = 122 *Grade 10:* B: *M* = 0.35; *SD* = 0.60; n = 112 A1: *M* = 0.27; *SD* = 0.34; n = 99 A2: *M* = 0.24; *SD* = 0.40; n = 149 A3: *M* = 0.24; *SD* = 0.34; n = 124
Sheffield antibullying program	Whitney et al. (1994)	*Primary:* B: 10%; N = 2519 A: 8.4%; N = 2370 *Secondary:* B: 6.2%; N = 4103 A: 4.3%; N = 4612	*Primary:* B: 26%; N = 2523 A: 23.1%; N = 2380 *Secondary:* B: 10%; N = 4116 A: 9.2%; N = 4620
Utrecht Healthy Schools	Busch et al. (2013)	OR: 0.38 (95% CI, 0.23–0.65)	OR: 0.38 (95% CI, 0.21–0.68)

Abbreviations: A, after; B, before; C, control; E, experimental; *M*, mean; *N*, sample size; *n*, group sample size.

### 8.1 Effect sizes

A meta‐analysis aims to estimate comparable effect sizes from multiple primary studies. The choice of effect size depends on how statistical information is reported by primary studies (Borenstein et al., 2009). In meta‐analyses such as this one, the data is largely presented in continuous (e.g., means, standard deviations, sample sizes) or dichotomous (e.g., prevalence or percentages) forms (Wilson, 2010). Thus, primary effect sizes estimated were Cohen's *d* and Odds Ratios.

As previously mentioned, we aimed to estimate one effect size for each *independent sample* included in primary studies. Therefore, where studies reported results separately for male and female participants, or primary and secondary school students, one effect size was calculated for each group.

For primary studies that presented results as percentages or frequencies of participants identifying as either bullies or victims, the odds ratio (OR) effect size was estimated. The ORs for before and after intervention time‐points were calculated independently. The CMA™ software that we used to analyze effect sizes in the present report did not allow us to enter raw data for before and after time‐points for primary studies that reported dichotomous outcomes separately. Thus, we were unable to use this software to calculate a pre‐post intervention estimate for these studies. Hence, these calculations were carried out manually,^5^ by the first author, using the method outlined by Farrington and Ttofi (2009).

Cohen's *d* was estimated for primary studies when results were reported in the form of continuous data. Cohen's *d* is estimated as the difference between experimental and control means divided by the pooled standard deviation (Wilson, 2010, p. 184). Effects were assigned a positive direction in cases where bullying was less in the experimental group compared to the control group or where the reduction in bullying outcomes was larger in the experimental group in comparison to the change in the control group. Following this logic, a negative effect was found when there was: (1) a larger reduction in the control group compared to the experimental group; or (2) there was no change or increase in bullying perpetration/victimization in the experimental group but a reduction or smaller increase in the control group.

For comparability, all effect sizes were converted to ODs. Summary mean effects for bullying perpetration, bullying victimization, and for each of the moderator subgroup are thus reported as odds ratios. In the present review, odds ratios greater than one represent a positive, or desirable, intervention effect. Namely, a reduction of bullying in the experimental group, that is comparably larger than the change in bullying in the control group. Therefore, the change is attributed to have occurred because of the intervention program. Similarly, odds ratios less than one represent a negative, or undesirable, intervention effect and odds ratios that equal one represents a null effect.

### 8.2 Corrections for clustering

As the present review aims to evaluate the effectiveness of school‐based antibullying programs, cluster‐randomized trials were included. Clustering is a common phenomenon in educational evaluations (Donner & Klar, 2002), and occurs when “clusters,” not individuals, are randomly assigned to experimental conditions (Higgins et al., 2011). In other words, primary studies sometimes assigned classes or schools to intervention and control conditions, rather than individual students.

Often this approach is utilized in evaluation studies to reduce treatment contamination and increase administrative convenience (Donner et al., 2001). However, one of the main issues with incorporating cluster‐randomized trials in a meta‐analysis is that participants within a cluster are likely to be more homogeneous than participants in another cluster (Higgins et al., 2011). Thus, the variance of estimates of treatment effectiveness will be under‐estimated (Donner & Klar, 2002, p. 2974). Clustering could occur for several reasons in studies included in the present report. For example: (1) classes of children, not individual children, were e randomized to intervention or control condition; (2) the intervention was implemented at the classroom level (i.e., to a class or group of children at one time); or (3) the intervention was targeted at teachers, who were trained to implement the intervention in their respective classrooms.

Therefore, effect sizes in the present meta‐analysis were corrected for the inclusion of clusters in primary studies. This is achieved by estimating a *design effect:*

(1) 1+(M−1)×ICC.

where *M* represents the mean cluster size in each study (e.g., the mean number of students per classroom^6^) and the ICC is the intraclass correlation coefficient.

The ICC is rarely reported by primary studies (Higgins et al., 2011; Valdebenito et al., 2018). Based on Murray and Blitse (2003), and subsequently the strategy followed by Farrington and Ttofi (2009), an ICC of 0.025 was assumed in the current meta‐analysis. The variances of effect sizes were then multiplied by this design effect estimated for each study. In the present meta‐analysis, there were only four studies where corrections for clustering were not required. Three studies (i.e., Berry & Hunt, 2009; Knowler & Frederickson, 2013; Meyer & Lesch, 2000) randomly assigned participants to experimental conditions, and Elledge et al. (2010) described an intervention that was not implemented in a classroom (i.e., the intervention occurred in one‐on‐one sessions with victims of bullying).

### 8.3 Computational models

The results of our meta‐analysis are presented using two different models. First, we will report the results as estimated using a random effects model that weights studies, largely in proportion to the between‐study variance and accounting for sampling error, thus allowing for the natural variation that occurs between primary studies (Borenstein et al., 2009). We also present the results under the MVA model (Jones, 2005; Farrington & Welsh, 2013). which uses the same estimation of a mean effect size as the fixed effects model in that it assigns greater weight to larger evaluations, but also accounts for the between‐study heterogeneity. The MVA model takes account of the heterogeneity of effect sizes to fit the data exactly and yields the same mean effect size as a fixed effect model, but with and increased confidence interval.^7^

Farrington and Welsh (2013) have argued that larger evaluations should be given more weight, and that adding to the variance of effect sizes in order to reduce the heterogeneity is not an optimal method of estimating the weighted mean effect size. When there is considerable heterogeneity in effect sizes, all studies tend to be given much the same weighting in a random effects model. Therefore, several effect sizes from independent samples in one study (e.g., a multisite evaluation) will have a greater weight in the random effects model than in the fixed effects model.

Comparing six models of estimating mean effect sizes for the impact on CCTV on crime rate, Farrington and Welsh (2013) found that five of the six models produced very similar mean odds ratio effect sizes, with the exception of the random effects model. In this case the random effects model estimated a much higher mean odds ratio (Farrington & Welsh, 2013, p. 11).

The MVA model is suggested as an alternative approach that overcomes the issues of the random effects model. This technique can be seen as an adjustment to the fixed effects model and combines both the strengths of the fixed effects model (i.e., larger studies = larger weights) and the random effects model (i.e., adjusting for highly probable between‐study variance), and has been used in several meta‐analyses from both the behavioral sciences (e.g., Portnoy & Farrington, 2015; Ttofi et al., 2016; Zych, Baldry, et al., 2019; Zych, Viejo, et al., 2019) and medical sciences, where this is known as the “Shore adjustment” (e.g., Ayieko et al., 2014; Carlos‐Wallace et al., 2016; Erren et al., 2009; Steinmaus et al., 2008).

A full review of the strengths and limitations of this model is beyond the scope of the current review. Therefore, in our current meta‐analysis we report mean effect sizes for the impact of antibullying programs on bullying perpetration and bullying victimization using both the random effects model and the MVA model. In later sections, we discuss the differences in the weighted mean effect sizes according to the model chosen.

### 8.4 Moderator analysis

In traditional empirical research when one wishes to compare two mean values to evaluate the difference between two participants, or two groups of participants, a *t* test is the standard statistical test. In meta‐analysis, we want to compare subgroups of studies rather than sub‐groups of individuals, so the analysis is slightly different. We followed guidelines provided by noted meta‐analysts for this type of analysis (Borenstein et al., 2009; Lipsey & Wilson, 2001).

Our approach involved two steps: (1) computing the mean effect and variance for each subgroup; and (2) comparing the mean effects between subgroups (Borenstein et al., 2009, p. 152). This approach has been used previously by researchers to conduct similar analyses (e.g., Kaminski et al., 2008; Ttofi & Farrington, 2011).

Comparing the mean effect sizes for subgroups involves a method that is analogous to a one‐way ANOVA in primary research (Hedges, 1982; Lipsey & Wilson, 2001; Wilson, 2002). The meta‐analyst creates mutually exclusive categories of primary studies and then compares the between‐studies (*Q*_*B*_) and the within‐studies (*Q*_*W*_) variance.

The between‐studies heterogeneity is the value used to evaluate whether the difference between subgroups is statistically significant (i.e., whether the difference in weighted mean effect sizes for subgroups is, at least partially, explained by the relevant intervention component). Similar to a one‐way analysis of variance, this approach partitions the variance and compares the variability between‐groups. The following formula is used to estimate the *Q*_*B*_:

$$Q_{B}=Q-Q_{w}$$

The degrees of freedom for the between‐studies heterogeneity is estimated as *j* − 1 and the statistical significance is determined using a *χ* ^2^ distribution. As *Q*_*B*_ is estimated using the weights assigned to observed effect sizes, the value will vary between the fixed effects model and the random effects model. *Q*_*B*_ is not reported for comparisons of subgroups with very unequal numbers of studies (e.g., location of the evaluation). Under the MVA model, the heterogeneity between groups is estimated by dividing the fixed effects *Q*_*B*_ by *Q/df*. The present report presents results from moderator analysis under both the random effects and MVA models.

### 8.5 Meta‐regression analysis

CMA™ version 3 software was used to conduct meta‐regression analysis to explore the relationship between continuous moderator variables and perpetration and victimization outcomes. Weighted regression analysis (Lipsey & Wilson, 2001) were used to explore which moderators were independently related to school bullying perpetration and victimization. Meta‐regression analyses were only conducted for continuous moderator variables.

Meta‐regression analyses were computed under a fixed effects model, and the standard error of regression coefficients were adjusted using the MVA model. The *Q* and *df* of Q for the mean summary effect sizes for subgroups were used to adjust the standard error to reflect between‐study variance.

## 9 RESULTS OF META‐ANALYSIS

In total, 100 studies were included in our meta‐analysis of the effectiveness of school‐based antibullying programs. From these evaluations, we were able to estimate 103 independent effect sizes. These are presented for bullying perpetration and bullying victimization outcomes in Tables 8 and 9, respectively. The majority of these effect sizes were estimated from studies that used RCT designs (*n* = 45 effect sizes) or BA/EC designs (*n* = 44 effect sizes). We estimated the remaining 14 effect sizes from age cohort designs.

**Table 9:** Meta‐analysis results: School‐bullying perpetration outcomes

*Study*	OR	CI	*z*	*p*
*Randomized controlled trials (36 evaluations; 40 effect sizes)*				
Baldry and Farrington (2004); Older	2.237	0.940–5.327	1.820	.069
Baldry and Farrington (2004); Younger	0.495	0.203–1.207	−1.546	.122
Beran and Shapiro (2005)	1.234	0.571–2.669	0.535	.593
Boulton and Flemington (1996)	0.871	0.443–1.712	−0.400	.689
Brown et al. (2011)	1.192	1.034–1.375	2.425	.015
Chaux et al. (2016)	1.620	1.123–2.336	2.583	.010
Cissner and Ayoub (2014)	0.793	0.459–1.370	−0.832	.406
Cross et al. (2011)	0.803	0.552–1.168	−1.147	.252
DeRosier and Marcus (2005)	1.208	0.769–1.897	0.819	.413
Domino (2013)	3.417	2.167–5.390	5.286	<.001
Espelage et al. (2015); Illinois	1.108	0.823–1.493	0.678	.498
Espelage et al. (2015); Kansas	1.052	1.093–1.274	4.245	.000
Fekkes et al. (2006)	1.105	0.620–1.970	0.339	.735
Fekkes et al. (2016)	2.514	1.264–5.003	2.627	.009
Fonagy et al. (2009)	1.248	0.946–1.646	1.564	.118
Frey et al. (2005)	1.058	0.813–1.376	0.419	.675
Garaigordobil and Martínez‐Valderrey (2015)	4.828	2.440–9.554	4.521	<.001
Holen et al. (2013)	2.127	1.688–2.679	6.400	<.001
Hunt (2007)	1.431	0.876–2.337	1.431	.152
Jenson et al. (2013)	1.099	0.551–2.190	0.267	.789
Kaljee et al. (2017)	0.592	0.496–0.707	−5.780	<.001
Kärnä et al. (2011b); Grades 4–6	1.101	1.000–1.212	1.963	.050
Kärnä et al. (2013); Grades 2–3	1.165	1.021–1.328	2.270	.023
Kärnä et al. (2013); Grades 8–9	1.075	0.987–1.171	1.667	.096
Krueger (2010)	2.423	0.621–9.456	1.274	.203
Li et al. (2011)	2.221	1.350–3.654	3.142	.002
McLaughlin (2009)	0.845	0.262–2.721	−0.283	.777
Meyer and Lesch (2000)	0.880	0.432–1.793	‐0.351	.726
Nocentini and Menesini (2016); Middle	1.562	1.184–2.062	3.154	.002
Nocentini and Menesini (2016); Primary	1.332	1.009–1.757	2.026	.043
Ostrov et al. (2015)	2.049	1.030–4.077	2.044	.041
Polanin (2015)	1.543	0.448–5.316	0.687	.492
Rosenbluth et al. (2004)	1.001	0.652–1.538	0.005	.996
Sprober et al. (2006)	0.654	0.285–1.499	−1.004	.315
Stallard et al. (2013)	1.057	0.774–1.443	0.346	.729
Trip et al. (2015)	1.243	0.868–1.780	1.188	.235
Tsiantis et al. (2013)	1.914	0.570–6.425	1.050	.294
Waasdorp et al. (2012)	1.282	1.173–1.401	5.480	<.001
Wölfer and Scheithauer (2014)	0.790	0.479–1.304	−0.922	.357
Yanagida et al. (2019)	1.399	0.699–2.798	0.949	.343
Random effects: RCTs	*1.240*	*1.118–1.375*	*4.069*	*<.001*
MVA fixed effects: RCTs	*1.171*	*1.082–1.268*	*3.913*	*<.001*
*Before‐after/experimental‐control designs (31 evaluations; 36 effect sizes)*				
Alsaker and Valkanover (2001)	1.134	0.579–2.222	0.367	.713
Andreou et al. (2007)	1.956	1.305–2.934	3.246	.001
Bergen 2/Olweus	1.770	0.974–3.218	1.872	.061
Bull et al. (2009)	2.455	0.343–17.563	0.894	.371
Ciucci and Smorti (1998)	1.198	0.581–2.470	0.491	.624
Evers et al. (2007); High	1.745	1.136–2.681	2.543	.011
Evers et al. (2007); Middle	1.547	0.909–2.630	1.609	.108
Finn (2009)	1.162	0.853–1.584	0.954	.340
Gini et al. (2003)	0.762	0.151–3.846	−0.329	.742
Gollwitzer et al. (2006)	0.968	0.451–2.079	−0.084	.933
Joronen et al. (2011)	1.210	0418–3.509	0.352	.725
Losey (2009)	0.903	0.618–1.322	−0.523	.601
Martin et al. (2005)	2.560	0.333–19.656	0.904	.366
Melton et al. (1998)	1.519	1.248–1.849	4.172	<.001
Menard and Grotpeter (2014)	1.085	0.855–1.377	0.672	.502
Menesini et al. (2003)	1.594	0.952–2.669	1.772	.076
Menesini et al. (2012; Study 1)	0.549	0.336–0.896	−2.399	.016
Ortega‐Ruiz et al. (2012)	1.230	0.893–1.693	1.268	.205
Palladino et al. (2012)	1.611	0.987–2.632	1.906	.057
Palladino et al. (2016; Trial 1)	1.803	1.148–2.832	2.559	.010
Palladino et al. (2016; Trail 2)	2.107	1.305–3.401	3.048	.002
Pepler et al. (2004)	1.883	1.030–3.444	2.055	.040
Pryce and Frederickson (2013)	0.543	0.324–0.909	−2.324	.020
Rahey and Craig (2002); Senior	1.223	0.629–2.378	0.594	.553
Rahey and Craig (2002); Junior	1.075	0.654–1.769	0.286	.775
Rawana et al. (2011)	0.565	0.240–1.330	−1.307	.191
Rican et al. (1996)	2.522	0.638–9.964	1.320	.187
Sapouna et al. (2010)	0.867	0.465–1.617	−0.450	.653
Silva et al. (2016)	1.259	0.562–2.822	0.559	.576
Sismani et al. (2014)	0.699	0.231–2.116	−0.634	.526
Solomontos‐Kountouri et al. (2016); 7th grade	1.029	0.832–1.274	0.267	.790
Solomontos‐Kountouri et al. (2016); 8th grade	0.593	0.431–0.817	−3.200	.001
Sutherland (2010)	0.754	0.519–1.095	−1.482	.138
Toner (2010)	0.890	0.427–1.859	−0.309	.757
Wong et al. (2011)	2.111	1.480–3.013	4.120	<.001
Yaakub et al. (2010)	1.085	0.935–1.260	1.071	.284
Random effects: BA/EC	*1.183*	*1.040–1.345*	*2.564*	*.010*
MVA fixed effects: BA/EC	*1.171*	*1.049–1.307*	*2.812*	*.005*
*Age cohort designs (14 evaluations; 14 effect sizes)*				
Busch et al. (2013)	0.380	0.226–0.639	−3.653	<.001
Ertesvåg & Vaaland (2004)	1.340	1.133–1.587	3.407	.001
Kärnä et al. (2011a); Nationwide	1.180	1.093–1.274	4.245	<.001
Limber et al. (2017); OBPP Pennsylvania	1.503	1.427–1.582	15.474	<.001
Olweus/Bergen 1	1.690	1.252–2.282	3.431	<.001
Olweus/New National	1.744	1.575–1.931	10.717	<.001
Olweus/Olso 1	2.140	1.182–3.876	2.512	.012
Olweus/Olso 2	1.751	1.354–2.263	4.275	<.001
O'Moore and Milton (2004)	2.119	0.809–5.547	1.530	.126
Pagliocca et al. (2007)	1.300	0.926–1.824	1.514	.130
Purugulla (2011)	1.274	0.923–1.758	1.473	.141
Roland et al. (2010)	1.417	1.368–1.468	19.430	<.001
Salmivalli et al. (2005)	1.310	1.068–1.606	2.596	.009
Whitney et al. (1994)	1.330	1.113–1.589	3.132	.002
Random effects: age cohorts	*1.474*	*1.392–1.560*	*13.416*	*<.001*
MVA fixed effects: age cohorts	*1.422*	*1.359–1.487*	*15.563*	*<.001*
**Overall: random effect model**	**1.308**	**1.239–1.380**	**9.792**	**<.001**
**Overall: MVA fixed effects model**	**1.324**	**1.271–1.379**	**13.403**	**<.001**

Abbreviations: BA/EC, before‐after/experimental control designs; CI, confidence intervals; MVA, multiplicative variance adjustment; OR, odds ratio; RCT, randomized controlled trial; Sig, statistically significant.

### 9.1 School‐bullying perpetration outcomes

Overall, we found that antibullying programs significantly reduced bullying perpetration under both computational models of meta‐analysis. The effect sizes for each evaluation are presented in Table 9. The mean summary effect sizes were similar under both the multivariance adjustment model (MVA: OR = 1.324; 95% CI 1.27–1.38; *z* = 13.4; *p* < .001; *I* ^*2*^ = 81.42) and the random effects model (RE: OR = 1.309; 95% CI: 1.24–1.38; *z* = 9.88; *p* < 0.001; *τ* ^2^ = 0.044).

This result indicates that participants in primary studies who received an antibullying intervention were less likely to report engaging in bullying others after completing the program in comparison to control students who did not partake in the program.

**Figure 3:**
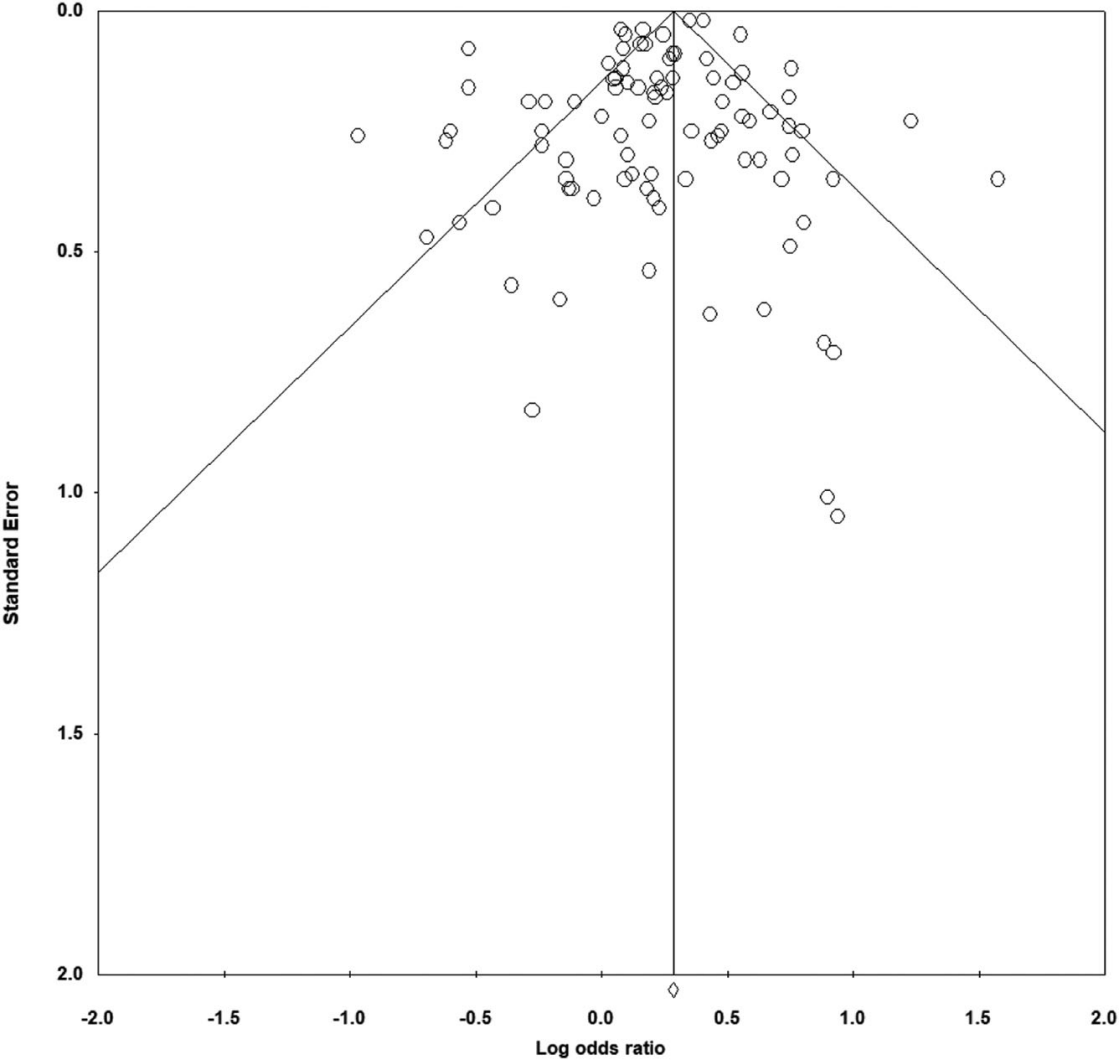
Publication bias analysis: school‐bullying perpetration

Analysis of the funnel plot (Figure 3) suggests that publication bias is not present, as studies are symmetrically distributed around the mean effect size. In addition, point estimates did not vary using Duval and Tweedie's trim and fill procedure under a random effects model (in both cases: OR = 1.308; 95% CI 1.240–1.380). Based on these results, it was reasonable to assume that publication bias was not likely.

### 9.2 School‐bullying victimization outcomes

Overall, we found that antibullying programs significantly reduced bullying victimization under both computational models of meta‐analysis. The effect sizes for each evaluation are presented in Table 10. The mean summary effect sizes were very similar under both the multivariance adjustment model (MVA: OR = 1.248; 95% CI 1.21–1.29; *z* = 12.06; *p* < .001; *I* ^*2*^ = 78.327) and the random effects model (RE: OR = 1.244; 95% CI: 1.19–1.31; *z* = 8.92; *p* < 0.001; *τ* ^2^ = 0.032).

**Table 10:** Meta‐analysis results: School‐bullying victimization outcomes

Study	OR	CI	*z*	*p*
*Randomized controlled trials (33 evaluations; 37 effect sizes)*				
Baldry and Farrington (2004); Older	2.874	1.207–6.842	2.385	.017
Baldry and Farrington (2004); Younger	1.011	0.425–2.407	0.025	.980
Berry and Hunt (2009)	9.865	3.129–31.102	3.907	<.001
Bonell et al. (2015)	1.000	0.761–1.315	0.000	1.000
Brown et al. (2011)	1.212	1.051–1.397	2.650	.008
Chaux et al. (2016)	1.236	0.857–1.783	1.136	.256
Cissner and Ayoub (2014)	0.632	0.342–1.167	−1.466	.143
Connolly et al. (2015)	0.917	0.638–1.317	−0.471	.638
Cross et al. (2011)	1.202	0.884–1.635	1.172	.241
DeRosier and Marcus (2005)	0.878	0.559–1.378	−0.567	.571
Domino (2013)	5.305	3.342–8.422	7.077	<.001
Espelage et al. (2015); Illinois	0.733	0.542–0.991	−2.091	.043
Espelage et al. (2015); Kansas	0.934	0.607–1.438	−0.309	.757
Fekkes et al. (2006)	1.006	0.672–1.506	0.029	.977
Fekkes et al. (2016)	2.430	1.188–4.970	2.433	.015
Fonagy et al. (2009)	1.182	0.895‐ 1.559	1.179	.238
Frey et al. (2005)	1.117	0.859–1.453	0.824	.410
Garaigordobil and Martínez‐Valderrey (2015)	2.213	1.171–4.182	2.447	.014
Hunt (2007)	1.259	0.771–2.056	0.920	.357
Jenson et al. (2013)	1.309	0.785–2.183	1.031	.303
Ju et al. (2009)	1.669	0.752–3.700	1.260	.208
Kaljee et al. (2017)	0.878	0.735–1.048	−1.440	.150
Kärnä et al. (2011b); Grades 4–6	1.273	1.156–1.401	4.926	<.001
Kärnä et al. (2013); Grades 2–3	1.148	1.028–1.282	2.452	.014
Kärnä et al. (2013); Grades 8–9	0.937	0.860–1.020	−1.500	.134
Knowler and Frederickson (2013)	0.573	0.196–1.669	−1.022	.307
McLaughlin (2009)	1.458	0.453–4.697	0.632	.527
Nocentini and Menesini (2016); Middle	1.668	1.264–2.201	3.615	<.001
Nocentini and Menesini (2016); Primary	1.600	1.212–2.111	3.321	.001
Polanin (2015)	1.214	0.352–4.184	0.307	.758
Rosenbluth et al. (2004)	0.699	0.515–0.949	−2.295	.022
Sprober et al. (2006)	1.031	0.450–2.361	0.073	.942
Topper (2011); Adventure	1.230	0.949–1.594	1.562	.118
Topper (2011); Preventure	0.762	0.480–1.209	−1.154	.249
Trip et al. (2015)	1.028	0.718–1.471	0.149	.882
Tsiantis et al. (2013)	1.857	0.749–4.602	1.337	.181
Yanagida et al. (2019)	3.725	1.656–8.377	3.180	.001
Random effects: RCTs	*1.200*	*1.078–1.336*	*3.331*	*.001*
MVA fixed effects: RCTs	*1.117*	*1.027–1.215*	*2.571*	*.010*
*Before‐after/experimental‐control designs (37 evaluations; 42 effect sizes)*				
Alsaker and Valkanover (2001)	3.114	1.609–6.029	3.371	.001
Andreou et al. (2007)	1.376	0.918–2.064	1.544	.123
Battey (2009)	0.773	0.352–1.696	−0.643	.521
Bauer et al. (2007)	1.013	0.793–1.294	0.100	.92
Beran et al. (2004)	1.101	0.657–1.843	0.366	.715
Bergen 2/Olweus	1.438	0.956–2.161	1.745	.081
Bull et al. (2009)	2.366	0.357–15.680	0.892	.372
Ciucci and Smorti (1998)	1.234	0.595–2.558	0.565	.572
Elledge et al. (2010)	0.492	0.138–1.751	−1.095	.273
Evers et al. (2007); High	0.915	0.565–1.482	−0.362	.718
Evers et al. (2007); Middle	2.257	1.288–3.953	2.846	.004
Finn (2009)	1.031	0.757–1.405	0.195	.845
Fox and Boulton (2003)	0.739	0.174–3.139	−0.410	.682
Gini et al. (2003)	0.405	0.116–1.414	−1.417	.157
Gollwitzer et al. (2006)	0.968	0.451–2.079	−0.084	.933
Herrick (2012)	0.661	0.205–2.137	−0.691	.490
Joronen et al. (2011)	2.482	0.894–6.890	1.745	.081
Kimber (2008)	1.833	1.122–2.993	2.420	.016
Losey (2009)	0.831	0.568–1.216	−0.953	.340
Martin et al. (2005)	1.970	0.231–16.781	0.620	.535
Melton et al. (1998)	1.058	0.869–1.287	0.559	.576
Menard and Grotpeter (2014)	1.395	1.099–1.770	2.739	.006
Menesini et al. (2003)	1.422	0.849–2.381	1.338	.181
Menesini et al. (2012; Study 1)	0.596	0.276–1.290	−1.313	.189
Ortega‐Ruiz et al. (2012)	1.394	1.012–1.918	2.036	.042
Palladino et al. (2012)	1.771	1.084–2.892	2.283	.022
Palladino et al. (2016; Trial 1)	2.270	1.445–3.566	3.559	<.001
Palladino et al. (2016; Trial 2)	2.306	1.432–3.712	3.437	.001
Pepler et al. (2004)	0.724	0.430–1.219	−1.214	.225
Pryce and Frederickson (2013)	1.406	0.840–2.355	1.297	.195
Rahey and Craig (2002); Junior	1.048	0.539–2.038	0.139	.889
Rahey and Craig (2002); Senior	0.582	0.354–0.958	−2.129	.033
Rawana et al. (2011)	0.565	0.240–1.330	−1.307	.191
Rican et al. (1996)	2.438	0.650–9.134	1.322	.186
Sapouna et al. (2010)	1.351	0.849–2.150	1.270	.204
Silva et al. (2016)	0.683	0.278–1.680	−0.830	.407
Sismani et al. (2014)	1.917	0.802–4.587	1.463	.143
Solomontos‐Kountouri et al. (2016); 7th grade	1.142	0.829–1.572	0.811	.417
Solomontos‐Kountouri et al. (2016); 8th grade	0.603	0.438–0.830	−3.100	.002
Sutherland (2010)	1.868	1.286–2.714	3.279	.001
Toner (2010)	1.482	0.710–3.094	1.048	.294
Williams et al. (2015)	1.326	0.921–1.909	1.516	.129
Random effects: BA/EC	*1.226*	*1.085–1.385*	*3.278*	*.001*
MVA fixed effects: BA/EC	*1.188*	*1.066–1.325*	*3.104*	*.002*
*Age cohort designs (14 evaluations; 14 effect sizes)*				
Busch et al. (2013)	0.380	0.211–0.684	−3.227	.001
Ertesvåg and Vaaland (2004)	1.181	0.995–1.400	1.908	.056
Kärnä et al. (2011a); Nationwide	1.210	1.137–1.287	6.045	<.001
Limber et al. (2017); OBPP Pennsylvania	1.189	1.148–1.232	9.655	<.001
Olweus/Bergen 1	2.889	2.141‐ 3.900	6.935	<.001
Olweus/New National	1.533	1.441–1.632	13.497	<.001
Olweus/Olso 1	1.809	1.230–2.662	3.010	.003
Olweus/Olso 2	1.480	1.243–1.762	4.404	<.001
O'Moore and Milton (2004)	1.990	0.977–4.053	1.895	.058
Pagliocca et al. (2007)	0.920	0.705–1.201	−0.610	.542
Purugulla (2011)	1.221	0.975–1.529	1.737	.082
Roland et al. (2010)	1.355	1.308–1.404	16.925	<.001
Salmivalli et al. (2005)	1.300	1.058–1.596	2.495	.013
Whitney et al. (1994)	1.140	1.004–1.295	2.015	.044
Random effects: age cohorts	*1.302*	*1.230–1.378*	*9.092*	*<.001*
MVA fixed effects: age cohorts	*1.289*	*1.288–1.353*	*10.218*	*<.001*
**Overall: random effects model**	**1.242**	**1.183–1.304**	**8.767**	**<.001**
**Overall: MVA fixed effects model**	**1.248**	**1.204–1.294**	**12.06**	**<.001**

Abbreviations: BA/EC, before‐after/experimental control designs; CI, confidence intervals; MVA, multiplicative variance adjustment; OR, odds ratio; RCT, randomized controlled trial; Sig, statistically significant.

This result suggests that students who participated in an antibullying program were significantly less likely to report being bullied by others after receiving the intervention in comparison to students who did not receive the intervention.

**Figure 4:**
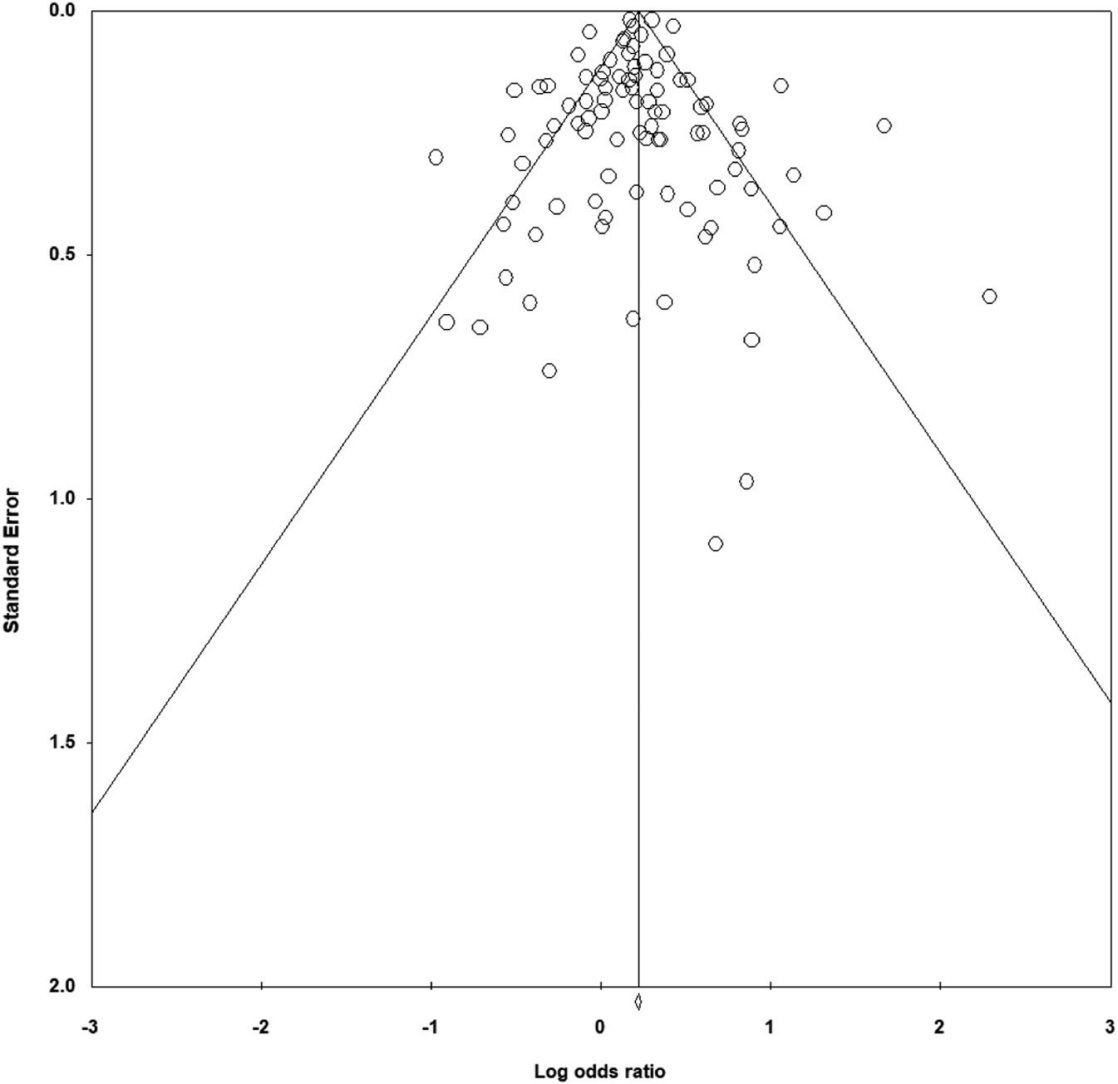
Publication bias analysis: school‐bullying victimization

The funnel plot in Figure 4 indicates that no publication bias is present in analysis of bullying victimization effect sizes, as the studies fall symmetrically around the mean effect size. Duval and Tweedie's trim and fill procedure highlighted some minor differences between observed effect sizes (OR = 1.245; 95% CI 1.186–1.306; *Q* = 460.97) and adjusted effect sizes (OR = 1.241; 95% CI 1.182–1.303; *Q* = 473.43). However, this difference is negligible. Based on these results, it was reasonable to assume that publication bias was not likely.

### 9.3 Analysis of heterogeneity

In a meta‐analysis, heterogeneity (*Q*) is the between‐study spurious variance that occurs partly because of true variation in effect sizes, but also as a result of random error (Borenstein et al., 2009). Heterogeneity is estimated as the excess variation that exists when we compare the total amount of between‐study variance and within‐study random error.

In the present meta‐analysis, there was significant heterogeneity between studies for both bullying perpetration (*Q* = 323.392; *df* = 85; *p* < 0.001; *I* ^*2*^ = 73.716) and bullying victimization (*Q* = 387.255; *df* = 87; *p *< 0.001; *I* ^*2*^ = 77.534) outcomes. Multiple moderator analyses were conducted to explore possible explanations for this heterogeneity.

### 9.4 Risk of bias analysis

Scores on each of the risk of bias items were summed to estimate a total risk of bias score. This continuous variable was then used to examine the relationship between effectiveness and risk of bias in meta‐regression models.

For perpetration outcomes, risk of bias was not associated with effect size under a random effects model of meta‐regression (*b* = 0.003; *SE* = 0.006; *z* = 0.50; *p* = .621) or under the MVA model (*b* = 0.014; *SE* = 0.014; *z* = 1.01; *p* = .156). Similarly, risk of bias scores did not significantly predict bullying victimization effect sizes under a random effects meta‐regression (*b* = 0.007; *SE* = 0.005; *z* = 1.30; *p* = .195) or the MVA model (*b* = 0.012; *SE* = 0.012; *z* = 1.006; *p* = .157).

### 9.5 Moderator analyses^8^

#### 9.5.1 Evaluation method

Our meta‐analysis further investigated the effectiveness of antibullying programs in relation to the methodological designs used by evaluation studies. The breakdown of results by methodological design is also shown in Tables 9 and 10 for bullying perpetration and victimization outcomes respectively.

Primary studies employing age cohort designs associated with the largest effect sizes for both bullying perpetration (OR = 1.474; 95% CI, 1.39–1.56; *p* < .001) and bullying victimization (OR = 1.302; 95% CI, 1.230–1.378; *p* < .001) under a random effects model. Similarly, AC studies were associated with the largest effect sizes under the MVA model also (perpetration OR = 1.422; 95% CI, 1.36–1.46; *p *< .001) and victimization OR = 1.289; 95% CI, 1.29–1.35; *p *< .001).

Under the MVA model of meta‐analysis, mean effect sizes were the same for RCT evaluations (OR = 1.171; 95% CI, 1.08–1.27; *p* < .001) and BA/EC evaluations (OR = 1.170; 95% CI, 1.05–1.31; *p* = .005) for bullying perpetration outcomes. Moreover, the differences between RCT evaluations (OR = 1.117; 95% CI, 1.03–1.22; *p* = .01) and BA/EC evaluations (OR = 1.188; 95% CI, 1.07–1.33; *p* = .002) were marginal for bullying victimization outcomes under the MVA model.

In relation to bullying victimization outcomes, before‐after/experimental‐control designs gave the second largest mean effect size (OR = 1.225; 95% CI, 1.085–1.383; *p* = 0.001), followed by RCTs (OR = 1.210; 95% CI, 1.091–1.342; *p* < .001) under a random effects model. However, the result was the opposite for bullying perpetration outcomes under a random effects model (RCT: OR = 1.244; 95% CI, 1.123–1.379; *p* < .001; BA/EC: OR = 1.187; 95% CI, 1.044–1.350; *p* = 0.009).

Due to the marginal differences and lack of clear pattern in which method was associated with the largest effect sizes (between RCT and BA/EC) further moderator analysis was not conducted.

#### 9.5.2 Location of intervention

**Figure 5:**
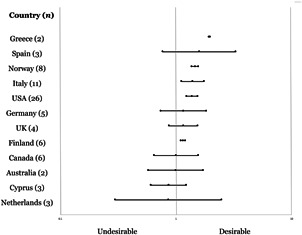
Forest plot of effect size by location: school‐bullying perpetration

**Figure 6:**
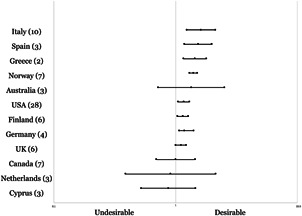
Forest plot of effect sizes by location: school‐bullying victimization

Mean effects for bullying perpetration and bullying victimization outcomes are presented graphically in Figures 5 and 6, respectively. Table 11 outlines the mean effects for each of the 12 countries for both bullying perpetration and victimization outcomes under both the MVA model and the random effects model.

**Table 11:** Moderator analyses results: Location of evaluation

	MVA model					Random effects model			
Location (*n*)	*OR*	*95% CI*	*p*	*Q (p)*	*I* ^*2*^	*OR*	*95% CI*	*p*	*τ* ^2^
*School bullying perpetration (n = 79 effect sizes)*									
Australia (2)	0.994	0.58–1.71	.980	3.364 (.067)	70.273	1.020	0.699–1.489	.916	.059
Canada (6)	1.00	0.65–1.56	.99	3.950 (.413)	26.582	0.919	0.683–1.235	.574	.021
Cyprus (3)	0.86	0.61–1.23	.42	8.660 (.013)	76.905	0.854	0.648–1.127	.266	.035
Finland (6)	1.15	1.11–1.21	<.001	4.982 (.418)	0.361	1.158	0.994–1.348	.059	.003
Germany (5)	1.16	0.74–2.83	.52	8.779 (.118)	54.437	1.062	0.796–1.416	.685	.021
Greece (2)	1.95	1.93–1.98	<.001	0.001 (.973)	NA	1.949	1.209–3.145	.006	.212
Italy (11)	1.39	1.12–1.75	.004	26.349 (.003)	62.048	1.370	1.141–1.643	.001	.056
Netherlands (3)	0.86	0.29–2.48	0.78	19.548 (<.001)	89.769	0.892	0.606–1.313	.563	.593
Norway (8)	1.47	1.37–1.57	<.001	30.430 (<.001)	76.996	1.659	1.436–1.918	<.001	.002
Spain (3)	1.59	0.77–3.29	.21	12.859 (.002)	84.447	1.791	1.222–2.624	.003	.490
UK (4)	1.16	0.87–1.54	.32	11.618 (.009)	74.178	1.029	0.807–1.313	.816	.036
United States (26)	1.38	1.24–1.54	<.001	65.804 (<.001)	62.008	1.293	1.171–1.428	<.001	.004
*School bullying victimization (n = 82 effect sizes)*									
Australia (3)	1.349	0.721–2.529	.351	12.15 (.002)	83.539	1.463	1.029–2.078	.034	0.316
Canada (7)	1.052	0.691–1.452	.982	17.121 (.004)	64.955	1.016	0.792–1.304	.902	0.069
Cyprus (3)	0.875	0.520–1.462	.614	10.982 (.004)	81.788	0.912	0.666–1.249	.564	0.095
Finland (6)	1.149	1.044–1.273	.008	32.574 (<.001)	84.650	1.180	1.004–1.388	.045	0.001
Germany (4)	1.229	1.068–1.414	.01	1.169 (.883)	156.629	1.220	0.886–1.678	.223	0.076
Greece (2)	1.446	1.161–1.803	<.001	0.349 (.555)	186.533	1.475	0.924–2.355	.104	0.092
Italy (10)	1.632	1.237–2.122	<.001	19.198 (.038)	53.120	1.592	1.314–1.928	<.001	0.035
Netherlands (3)	0.911	0.389–2.136	0.833	15.947 (<.001)	87.458	0.914	0.631–1.326	.636	0.415
Norway (7)	1.404	1.302–1.515	<.001	39.737 (<.001)	84.901	1.548	1.326–1.809	<.001	0.014
Spain (3)	1.537	1.19 0– 1.987	<.001	1.670 (.434)	19.760	1.610	1.091–2.377	.016	0.053
UK (6)	1.110	1.011–1.229	.041	4.056 (.541)	23.274	1.060	0.831–1.352	.639	0.017
United States (28)	1.168	1.050–1.303	.005	90.373 (<.001)	70.124	1.105	0.996–1.227	.059	0.019

Evaluations conducted in Greece were associated with the largest effect sizes for bullying perpetration outcomes, followed by Norway, Italy, United States, and Finland under the MVA model of meta‐analysis. Evaluations conducted in Italy were associated with the largest mean effect sizes in relation to bullying victimization, followed by Spain, Norway, United States, and Finland under the MVA model of meta‐analysis. Additionally, evaluations conducted in Germany and the UK gave significant mean effects when computed using the MVA model.

Under the random effects model, Greek evaluations were similarly associated with the largest effect sizes for bullying perpetration, followed by Spanish and Norwegian evaluations. Evaluations conducted in Italy and the United States were also associated with significant mean effects for reductions in bullying perpetration. In relation to bullying victimization, evaluations conducted in Spain and Italy were associated with very similar mean effect sizes and were the largest of the 12 effect sizes, followed by evaluations conducted in Norway. Evaluations conducted in Australia were also associated with significant mean effects in reducing bullying victimization (*p* < .05) and evaluations conducted in Finland and the United States were nearly statistically significant (*p* = .05 and *p* = .06, respectively) under the random effects model.

Due to the large number of different countries and the unequal number of studies in each location, further subgroup analyses were not conducted.

#### 9.5.3 Publication type and year

Table 12 outlines the mean summary effect sizes for each of the publication type moderators for bullying perpetration and victimization outcomes. Evaluations for which data was received via email correspondence from evaluators gave the largest mean effect sizes for both bullying perpetration and bullying victimization. Differences in the mean effect sizes for evaluations reported via unpublished dissertations, either masters or doctoral theses, gave the smallest mean effect sizes for both bullying perpetration and victimization outcomes. Subgroup analysis was not conducted further using these categorizations due to the imbalance in numbers of evaluations in each category (i.e., evaluations were overwhelmingly published in peer‐reviewed journal article format).

**Table 12:** Moderator analyses results: Publication type

	MVA model					Random effects model			
Publication type (*n*)	*OR*	95% CI	*p*	*Q (p)*	*I* ^2^	OR	95% CI	*p*	*τ* ^2^
*School bullying perpetration (n = 90 effect sizes)*									
Article (67)	1.315	1.251–1.383	<.001	409.65 (*p* < .001)	83.89	1.230	1.146–1.321	<.001	.044
Chapter (2)	1.278	0.909–1.796	.158	3.98 (*p* = .264)	24.58	1.321	0.926–1.885	.125	.033
Correspondence (4)	1.745	1.692–1.799	<.001	0.51 (*p* = .972)	0.00	1.745	1.602–1.901	<.001	.000
Dissertation (12)	1.040	0.878–1.232	.649	7.74 (*p* = .356)	9.59	1.037	0.870–1.237	.686	.006
Gov Report (3)	1.311	0.969–1.773	.079	7.241 (*p* = .027)	72.38	1.154	0.805–1.654	.435	.070
*School bullying victimization (n = 93 effect sizes)*									
Article (72)	1.223	1.176–1.272	<.001	297.08 (*p*<.001)	76.10	1.209	1.137–1.286	<.001	.027
Chapter (2)	1.267	0.316–5.083	.738	11.55 (*p* = .001)	91.34	1.480	0.354–6.179	.591	.972
Correspondence (4)	1.568	1.367–1.799	<.001	17.41 (*p* = .001)	82.77	1.791	1.419–2.261	<.001	.042
Dissertation (12)	1.107	0.962–1.274	.156	18.04 (*p* = .081)	39.01	1.073	0.934–1.280	.267	.026
Gov Report (3)	1.006	0.848–1.194	.946	2.46 (*p* < .001)	18.67	0.993	0.826–1.193	.939	.006

However, additional analysis was conducted to examine any potential differences between peer reviewed and nonpeer reviewed evaluations. Therefore, the above categories were collapsed, and evaluations reported by dissertation, chapter, correspondence and governmental reports (perpetration *n* = 23; victimization *n* = 21) were compared to evaluations published via peer‐reviewed journal article.

Under the MVA model, non‐peer‐reviewed evaluations gave a larger (OR = 1.493; 95% CI, 1.266–1.761; *p* < .001) mean effect size than peer‐reviewed evaluations (see Table 11). Moreover, moderator analysis indicated that the difference was statistically significant (*Q*_*B*_ = 12.861; *df* = 1; *p* < .001). However, under the random effects model, both groups gave similar effect sizes for bullying perpetration outcomes, and the difference between peer‐reviewed (see Table 11) and non‐peer‐reviewed (OR = 1.309; 95% CI, 1.137–1.508; *p* < .001) was not statistically significant (*Q*_*B*_ = 0.595; *df* = 1; *p* = .441).

For bullying victimization outcomes, similar results were obtained. Under the MVA model, non‐peer‐reviewed evaluations gave statistically significant larger mean effect sizes (OR = 1.403; 95% CI, 1.262 1.560; *p *< .001) than peer‐reviewed evaluations (see Table 11; *Q*_*B*_ = 27.197; *df* = 1; *p* < .001). Yet, there was a marginal difference under the random effects model between peer‐reviewed (see Table 11) and non‐peer‐reviewed (OR = 1.231; 95% CI, 1.059–1.431; *p* = .007) and the difference was not statistically significant (*Q*_*B*_ = 0.048; *df* = 1; *p* = .827).

The mean summary effect size for “2009” studies on the year of publication moderator was OR = 1.487 (95% CI, 1.430–1.546; *p *< .001) under the MVA model and OR = 1.411 (95% CI, 1.315–1.513; *p* < .001) under the random effects model for bullying perpetration outcomes. Across both computational models these summary effects were larger than those for studies labeled “2016” on bullying perpetration for the MVA model (OR = 1.243; 95% CI, 1.667–1.324; *p *< .001) and the RE model (OR = 1.184; 95% CI, 1.087–1.289; *p* < .001). Moderator analysis analogous to the ANOVA showed that this difference was statistically significant (*Q*_*B*_ = 76.412; *df* = 1; *p *< .001) under fixed effects and mixed effects analysis (*Q*_*B*_ = 9.676; *df* = 1; *p* = .002).

In relation to bullying victimization, the mean summary effect size for studies labeled “2009” was larger (OR = 1.322; 95% CI, 1.220–1.432; *p *< .001) under the MVA model than the mean summary effect size for studies labeled “2016” (OR = 1.229; 95% CI, 1.175–1.285; *p* < .001). Moderator analysis analogous to the ANOVA found that this difference was statistically significant (*Q*_*B*_ = 10.115; *df* = 1; *p* = .001) but the difference between odds ratios was marginal. However, under the random effects model the minimal difference between the “2009” studies (OR = 1.215; 95% CI, 1.094–1.350; *p *< .001) was not statistically different to the mean summary effect size for “2019” studies (OR = 1.223; 95% CI, 1.139–1.313; *p *< .001; *Q*_*B*_ = 0.010; *df* = 1; *p* = .920).

#### 9.5.4 Intervention program

The mean summary effect sizes for 10 different intervention programs in relation to reducing bullying perpetration behaviors and 9 different intervention programs in relation to reducing bullying victimization behaviors. Table 13 outlines the effectiveness of specific antibullying programs in reducing both school‐bullying perpetration and victimization. The effectiveness of these programs varied greatly.

**Table 13:** Moderator analyses results: Intervention program

	MVA model					Random effects model			
Intervention (*n*)	OR	95% CI	*p*	*Q (p)*	*I* ^2^	OR	95% CI	*p*	*τ* ^2^
*School bullying perpetration (n = 36 effect sizes)*									
BPYS (3)	1.065	0.950–1.193	.279	0.252 (.616)	693.651	1.054	0.787–1.412	.724	.061
Fairplayer.manual (2)	0.846	0.498–1.439	.539	1.198 (.274)	16.528	0.855	0.507–1.443	.557	.093
KiVa (6)	1.143	1.075–1.215	<.001	9.347 (.096)	46.507	1.180	1.063–1.309	.002	.001
NoTrap! (4)	1.378	0.764–2.483	.286	18.301 (<.001)	83.607	1.374	1.059–1.782	.017	.246
OBPP: Overall (12)	1.532	1.438–1.631	<.001	22.292 (.014)	55.141	1.501	1.358–1.659	<.001	.002
OBPP: USA (6)	1.473	1.374–1.579	<.001	10.604 (.060)	52.848	1.349	1.185–1.535	<.001	.002
OBPP: Norway (5)	1.749	1.695–1.804	<.001	0.498 (.974)	703.213	1.759	1.503–2.059	<.001	.018
Second Step (3)	1.101	1.027–1.181	<.001	0.304 (.859)	557.895	1.107	0.879–1.395	.387	.029
Steps to Respect (2)	1.160	1.052–1.279	<.001	0.609 (.435)	64.204	1.142	0.934–1.397	.197	.001
ViSC (5)	0.952	0.730–1.241	.714	12.237 (.016)	67.312	0.949	0.785–1.149	.596	.045
*School bullying victimization (n = 35 effect sizes)*									
BPYS (3)	1.349	1.189–1.530	<.001	0.734 (.693)	172.48	1.323	0.962–1.819	.085	.036
KiVa (6)	1.160	1.033–1.302	<.001	41.222 (<.001)	90.296	1.240	1.063–1.447	.006	.021
NoTrap! (4)	1.836	1.150–2.931	<.001	9.929 (.019)	69.785	1.772	1.296–2.424	<.001	.165
OBPP: Overall (12)	1.264	1.158–1.379	<.001	102.667 (<.001)	89.286	1.285	1.137–1.451	<.001	.039
OBPP: Norway (5)	1.172	1.122–1.224	<.001	10.141 (.119)	60.556	1.053	0.899–1.233	.522	.017
OBPP: USA (7)	1.566	1.391–1.762	<.001	17.579 (.002)	65.868	1.726	1.424–2.092	<.001	.016
Second Step (3)	0.807	0.666– 0.977	<.001	1.249 (.535)	60.128	0.832	0.593–1.168	.289	.024
Steps to Respect (2)	1.190	1.113–1.272	<.001	0.287 (.592)	248.432	1.171	0.884–1.551	.273	.008
ViSC (5)	0.952	0.635–1.429	.813	20.146 (.001)	80.145	1.004	0.781–1.291	.975	.190

In relation to school‐bullying perpetration outcomes, the OBPP was associated with the largest mean effect sizes. In addition, evaluations of the OBPP in Norway were associated with larger summary effect sizes than evaluations of OBPP conducted in the United States. However, the difference was not statistically significant for school‐bullying perpetration outcomes when moderator analysis analogous to the ANOVA was conducted (*Q*_*b*_ = 3.65; *df* = 1; *p* = 0.06).

Other programs were significantly effective in reducing school‐bullying perpetration behaviors, for example KiVa, Second Step, and Steps to Respect. Positive effect sizes (i.e., OR > 1) were also observed for the BPYS and NoTrap! programs but these effects were not statistically significant in relation to reduction in bullying perpetration outcomes. Negative effects were found for two antibullying programs, the fairplayer manual and ViSC, although these effects were not statistically significant.

In relation to school‐bullying victimization outcomes, NoTrap! was associated with the largest mean effect size, followed by the BPYS Program, and then the OBPP. Our analysis identified that other antibullying programs were also significantly effective in reducing school‐bullying victimization, for example, Steps to Respect and KiVa.

Again, effect sizes for the OBPP varied between evaluations conducted in Norway and evaluations conducted in the United States for bullying victimization outcomes. Moreover, our analysis found that the difference in the magnitude of these effect sizes was statistically significant (*Q*_*b*_ *=* 74.95; *df* = 1; *p *< 0.001). Our analysis also identified negative effects of the Second Step program in relation to bullying victimization outcomes. Evaluations of the ViSC program also had a negative effect on bullying victimization, although this effect was not statistically significant.

#### 9.5.5 Unit of allocation/randomization

Table 14 outlines the mean effects for subgroups of studies according to how participants were allocated to experimental or control groups. Results are presented for bullying perpetration and victimization outcomes for all studies that allocated studies in classes, schools, or individual students. The mean effects for RCT and BAEC for each allocation unit are also presented separately.

**Table 14:** Moderator analyses results: Unit of allocation/randomization

	*MVA model*					Random effects model			
Unit of allocation (*n*)	OR	95% CI	*p*	*Q (p)*	*I* ^2^	OR	95% CI	*p*	*τ* ^2^
**School bullying perpetration (*n* = 70 effect sizes)**									
*All designs*									
Classes (19)	1.319	1.087–1.601	<.001	44.763 (<.001)	59.788	1.286	1.044–1.586	.018	.338
Schools (44)	1.163	1.091–1.240	<.001	136.032 (<.001)	68.390	1.188	1.098–1.286	<.001	.185
Students (7)	0.725	0.489–1.074	.109	47.208 (<.001)	87.290	1.465	0.749–2.865	.265	.771
*Randomized controlled trials (n = 39 effect sizes)*									
Classes (11)	1.295	0.952–1.761	.099	36.998 (<.001)	72.972	1.246	0.892–1.740	.197	.460
Schools (22)	1.184	1.107–1.266	<.001	57.455 (<.001)	63.450	1.242	1.141–1.352	<.001	.135
Students (6)	0.720	0.471–1.101	.129	45.737 (<.001)	89.068	1.407	0.699–2.835	.339	.776
*Quasi‐experimental designs with before and after measures (n = 31 effect sizes)*									
Classes (8)	1.353	1.109–1.651	<.001	7.648 (.365)	8.473	1.349	1.099–1.655	.004	.008
Schools (22)	1.091	0.942–1.263	.244	75.193 (<.001)	72.072	1.108	0.940–1.305	.223	.095
Students (1)	2.046	0.340–17.807	.373	NA	NA	2.460	0.340–17.807	.373	.001
**School bullying victimization (*n* = 71 effect sizes)**									
*All designs*									
Classes (15)	1.529	1.168–2.001	<.001	50.377 (<.001)	72.210	1.523	1.138–2.038	.005	.462
Schools (47)	1.164	1.063–1.275	<.001	132.738 (<.001)	65.345	1.181	1.068–1.305	.001	.261
Students (9)	0.940	0.717–1.232	.654	27.401 (.001)	70.804	1.157	0.771–1.734	.482	.455
*Randomized controlled trials (n = 32 effect sizes)*									
Classes (7)	1.716	0.967–3.046	.065	39.039 (<.001)	84.631	1.637	0.876–3.058	.122	.568
Schools (19)	1.156	1.028–1.300	<.001	49.942 (<.001)	63.958	1.165	1.025–1.324	.019	.046
Students (6)	0.943	0.677–1.314	.729	25.486 (<.001)	80.381	1.203	0.777–1.863	.407	.220
*Quasi‐experimental designs with before and after measures (n = 38 effect sizes)*									
Classes (8)	1.418	1.144–1.757	<.001	9.662 (.209)	27.551	1.422	1.130–1.789	.003	.029
Schools (28)	1.175	1.016–1.358	<.001	82.710 (<.001)	67.356	1.186	1.013–1.389	.034	.107
Students (2)	0.943	0.193–3.335	.762	1.825 (.177)	45.205	0.917	0.203–4.133	.910	.558

In relation to bullying perpetration outcomes, under the MVA model, studies that assigned participants in classes were associated with the largest effect sizes. However, the difference between the mean effect for all evaluations that used classes or schools as the unit of allocation were verging on statistically significance (*Q*_*b*_ *=* 3.705, *df* = 1, *p* = .054). Under the random effects model, evaluations that assigned students to experimental conditions were associated with the largest effect size for bullying perpetration outcomes when all designs were included, and for RCT evaluations and BA/EC evaluations individually. However, the mean effect size for many of the subgroups were not collectively statistically significant overall under the random effects model.

Similarly, under the MVA model, evaluations conducted using a RCT design, and assigned classes to conditions, were associated with the largest effect size for bullying perpetration, although the mean group for this subgroup was not statistically significant. Moreover, moderator analysis analogous to the ANOVA found that the difference in the mean effect size for RCT designs that assigned classes to experimental and control conditions were not statistically different to RCT designs that assigned schools to experimental and control conditions (*Q*_*b*_ *=* 1.140, *df* = 1, *p* = .286*)*.

In relation to BAEC designs, evaluations that assigned students to experimental conditions were associated with the largest mean effect size, although the effect was not statistically significant. However, the difference between the mean effect for BAEC evaluations that assigned classes and those that assigned schools to conditions was statistically significant under the MVA model (*Q*_*b*_ *=* 4.551, *df* = 1, *p* = .033).

For bullying victimization outcomes, studies where the unit of allocation was classes of participants were associated with the largest effect sizes, followed by schools and individual students under the MVA model. The difference between studies that allocated classes and studies that allocated schools was statistically significant (*Q*_*b*_ *=* 12.450, *df* = 1, *p *< .001). This pattern was observed when all designs were included, and for the subgroup of RCT evaluations and the subgroup of BA/EC evaluations. Thus, when participants were assigned in classes the mean effect size for these RCT evaluations were significantly associated with larger effect sizes (*Q*_*b*_ *=* 13.590, *df* = 1, *p *< .001) for reductions in bullying victimization than RCT evaluations that assigned schools. Yet the difference between the mean effect sizes for BA/EC evaluations that assigned classes were not statistically significant (*Q*_*b*_ *=* 3.359, *df* = 1, *p* = .067) than BA/EC evaluations that assigned schools to experimental conditions.

#### 9.5.6 Conflict of interest

COI was a categorical moderator variable with three levels: high‐risk (H), low‐risk (L), and possible‐risk (P). Moderator analysis analogous to the ANOVA was conducted so as to assess the differences between evaluations on each level. Studies categorized as possible‐risk on COI variable were excluded from subgroup comparisons to establish the differences between evaluations that were clearly high‐risk and evaluations that were clearly low‐risk. Table 15 outlines the mean summary effects for each group for both bullying perpetration and bullying victimization outcomes.

**Table 15:** Moderator analyses results: Conflict of interest

	MVA model					Random effects model			
COI‐risk (*n*)	OR	95% CI	*p*	*Q (p)*	*I* ^2^	OR	95% CI	*p*	*τ* ^2^
*School bullying perpetration (n = 86 effect sizes)*									
High (40)	1.375	1.309–1.444	<.001	196.882 (<.001)	80.191	1.330	1.232–1.435	<.001	.025
Possible (10)	1.390	1.185–1.631	<.001	13.468 (.142)	33.175	1.445	1.182–1.766	.844	.030
Low (36)	1.146	1.024–1.282	.017	214.119 (<.001)	83.654	1.123	0.988–1.277	.077	.106
*School bullying victimization (n = 89 effect sizes)*									
High (40)	1.270	1.213–1.329	<.001	218.053 (<.001)	82.114	1.324	1.232–1.422	<.001	.022
Possible (10)	1.090	0.957–1.241	.192	16.538 (.056)	45.581	1.087	0.908–1.301	.365	.030
Low (39)	1.129	1.010–1.262	.033	162.359 (<.001)	76.595	1.132	0.997–1.285	.056	.101

*Note*: Four studies and six studies were excluded from the present moderator analysis for perpetration and victimization outcome respectively as not enough information was available.

Moderator analyses found that the difference between high‐risk and low‐risk studies on COI variable was statistically significant for bullying perpetration outcomes under both the MVA model (*Q*_*B*_ = 50.129; *df* = 1; *p *< .001) and the random effects model (*Q*_*B*_ = 4.900; *df* = 1; *p* = .027). This suggests that evaluations considered to have high COI were associated with larger overall effect sizes for bullying perpetration. Similarly, high‐risk COI studies were significantly associated with slightly larger effect sizes for bullying victimization in comparison to low‐risk COI studies when compared under both the MVA model (*Q*_*B*_ = 16.127; *df* = 1; *p* < .001) and the random effects model (*Q*_*B*_ = 4.449; *df* = 1; *p* = .035).

#### 9.5.7 Program specificity

The majority of evaluations included in our meta‐analysis were of highly specific intervention programs, that is, those that targeted bullying behaviors and no other outcomes. Consistently across computational model and both perpetration and victimization outcomes these subgroups were associated with the largest mean effect sizes. These results are presented in Table 16. Additionally, highly specific programs were the only subgroup of evaluations that gave a statistically significant mean summary effect under both the MVA model and the random effects model for bullying victimization outcomes. In relation to bullying perpetration outcomes, the subgroup of evaluations that were coded as “medium” on the program specificity moderator were associated with a statistically significant mean effect size under the MVA model (*p *< .001) and the random effects model (*p* = .036).

**Table 16:** Moderator analyses results: Program specificity

	MVA model					Random effects model			
Specificity (*n*)	OR	95% CI	*p*	*Q (p)*	*I* ^2^	OR	95% CI	*p*	*τ* ^2^
*School bullying perpetration (n = 85 effect sizes)*									
High (66)	1.343	1.285–1.403	<.001	279.036 (<.001)	76.706	1.295	1.209–1.388	<.001	.004
Medium (14)	1.208	1.038–1.404	<.001	108.843 (<.001)	88.056	1.165	1.009–1.343	.036	.013
Low (5)	1.014	0.625–1.645	.955	24.652 (.001)	83.774	0.996	0.761–1.303	.976	.135
*School bullying victimization (n = 88 effect sizes)*									
High (63)	1.262	1.210–1.317	<.001	328.981 (<.001)	81.154	1.292	1.212–1.377	<.001	.007
Medium (16)	1.022	0.889–1.173	.763	33.055 (.005)	54.621	1.061	0.919–1.225	.422	.010
Low (9)	1.059	0.824–1.347	.676	25.746 (.001)	68.927	1.008	0.833–1.219	.937	.050

## 10 DISCUSSION

### 10.1 Summary of main findings

Overall, our updated meta‐analysis found that school‐based antibullying programs are effective in reducing both school‐bullying perpetration and victimization. For school‐bullying perpetration the weighted mean OR = 1.324 under the MVA model, or OR = 1.309 under a random‐effects model (RE) were associated with reductions of approximately 19–20%.^9^ In comparison, the weighted mean ORs for bullying victimization outcomes were 1.248 and 1.242 under the MVA model and the random effects model respectively. These mean effect sizes correspond to an approximate reduction in bullying victimization of 15–16%. These results suggest that the included interventions were slightly more effective at reducing school‐bullying perpetration than school‐bullying victimization.

The results of this meta‐analysis are consistent with findings from most of previous reviews that indicate that antibullying programs have a small but significant effect, with some variations in overall results being attributable to methodological differences in inclusion and exclusion criteria (Ttofi et al., 2014). Our mean effect sizes are also consistent with the earlier review (Farrington & Ttofi, 2009; Ttofi & Farrington, 2011), although the differences further outline that moderator variables such as methodological design may be responsible for variability. For example, the weighted mean effect sizes for both bullying perpetration and bullying victimization outcomes estimated in the earlier Campbell report were larger than those estimated in the present report.

Yet, we included publication year as a categorical moderator variable in the present analysis. We found that more recent studies (i.e., those that were *not* included by Farrington & Ttofi, 2009) were significantly different to studies that were included in the earlier review. Namely, recent studies were actually associated with significantly larger effect sizes for both bullying perpetration and victimization outcomes (see Section 8.5.3).

Therefore, as we excluded studies considered to have utilized less scientifically rigorous methodological designs this may explain the differences in the weighted mean effect sizes. Specifically, we excluded evaluations conducted using “other experimental‐control designs,” described in the earlier review as evaluations in which participants were assigned to experimental and control conditions but bullying outcomes were only measured after implementation of the intervention. Thus, attributing any change in behaviors to the intervention is potentially risky because there may be other reasons why a positive effect of the intervention was observed. For example, the experimental and control groups were not comparable at baseline, but this remains unknown as no measure of bullying was obtained.

Thus, the inclusion of these less methodologically rigorous evaluations may explain why the weighted mean effects sizes reported in the earlier review were larger than those reported in the current report, but our moderator analysis found a contradictory pattern. The following sections of this report will aim to discuss the findings obtained by our moderator analyses and also the strengths and limitations of the current analysis and potential avenues for future research. The heterogeneity in this meta‐analysis was very large for both bullying perpetration and victimization outcomes. This may suggest that there was a wide range of effects across programs and we may not be able to explain differences using moderator analysis.

### 10.2 Moderator analyses

#### 10.2.1 Evaluation method

Under both the MVA and random effects models, evaluations conducted using age cohort designs were identified to be, collectively, the most effective, or at least associated with the largest mean effect sizes. This is consistent with Farrington and Ttofi's (2009) review. This methodological design was first introduced as an evaluation design for the OBPP (Olweus, 1991). This approach has been criticized for the potential threats to internal validity, history and testing effects (Farrington & Ttofi, 2009, p. 15). It has been suggested that this design avoids the threats of aging and maturation effects, as individuals within the same school act as a control group for same‐aged experimental participants (Olweus, 2005a). However, this design is vulnerable to cross‐contamination between experimental and control participants which would impact the overall effectiveness. Notably, intervention researchers have tested the OBPP with other methodological designs (e.g., Bauer et al., 2007) which resulted in smaller effects.

Interestingly, the pattern between RCTs and BA/EC designs was less clear. In relation to bullying victimization outcomes, evaluations using BA/EC designs appear to be more effective than evaluations using RCT designs. However, for bullying perpetration outcomes, evaluations using RCT designs appear to be more effective than evaluations that utilized BA/EC designs. Further research is needed to understand these effects. However, the nature of these analyses is correlational and the differences between effect sizes are marginal. Thus, no concrete conclusion can be drawn in relation to the association between randomized and nonrandomized quasi experimental designs and effect size in the present context.

#### 10.2.2 Unit of allocation/randomization

In theory, RCTs are the best method of evaluation of interventions because random allocation ensures that any observed differences between experimental and control groups occurs as a result of experimental manipulation, thus giving the best possible internal validity (Farrington, 1983, 2003). However, the unit of random allocation can have an impact on internal validity. For example, we assume that individuals are randomly assigned to experimental and control conditions, so that RCT designs adequately account for the random variation that occurs in real‐world research (Weisburd, 2003).

However, in practice, evaluations of antibullying programs may be more likely to assign groups of individuals, for example in terms of classrooms or schools, to experimental conditions rather than individual students. This is true for both randomized (e.g., classrooms, Chaux et al., 2016; or schools, Espelage et al., 2015) and nonrandomized (e.g., classrooms, Ortega‐Ruiz et al., 2012; or schools, Rawana et al., 2011) methodologies. When this is the case, we need larger numbers to ensure adequate statistical conclusion validity and avoid issues of selection effects and differential attrition (Farrington & Ttofi, 2009; Ttofi & Farrington, 2011). There was a lot of variation in the unit of allocation in our primary studies, which may explain why we did not find that one methodological design was more effective than another.

Moreover, the majority of included evaluations did not use the same unit for allocation and analysis, thus, posing a threat to our results. We approach the results therefore with caution, favouring more conservative estimates. Furthermore, the relationship between the unit of randomization/allocation moderator variable and the effect sizes for bullying perpetration and victimization outcomes was unclear. Whether or not the differences between subgroups of evaluations that assigned classes or schools to experimental conditions were statistically significant or not depended on the computational model used and the bullying outcome in question. For bullying perpetration, the differences between studies based on unit of allocation were not statistically significant for randomized and nonrandomized studies. For bullying victimization outcomes, studies where classes were the unit of allocation were associated with the largest effect sizes when all designs where included and for randomized evaluations, but not for nonrandomized evaluations, separately.

Risk of bias analysis also found that a large number of RCT studies were categorized as being high risk for allocation‐related items on the EPOC tool. Therefore, the differences observed between primary evaluations in our meta‐analysis may be due to the observation that largely the unit of allocation and the unit of analysis were not the same in primary studies. However, further analysis and investigation is needed to better understand these results.

#### 10.2.3 Location of intervention

Overall, the results of our meta‐analysis are consistent with previous findings and show that school‐based antibullying programs have a modest but significant effect in reducing bullying behaviors. However, our meta‐analysis included evaluations of antibullying programs from a wide range of countries and specific intervention programs, far more than previous meta‐analyses (e.g., Cantone et al., 2015; Chalamandaris & Piette, 2015; Evans et al., 2014; Jiménez‐Barbero et al., 2012, 2016). As a result, the results of this meta‐analysis are robust and have implications for bullying research globally.

Our analysis identifies that antibullying programs worldwide are effective in reducing school‐bullying perpetration and victimization by significant amounts. Moreover, evaluations in different countries appear to vary in effectiveness. In Greece, where evaluations included in our meta‐analysis were associated with the largest effect sizes, school‐bullying perpetration behaviors were reduced by approximately 40%. Evaluations conducted in the Norway, Italy and the United States were also effective in reducing bullying perpetration by approximately 21–25%.

Antibullying programs implemented and evaluated in Italy were associated with the largest reduction in school‐bullying victimization in our meta‐analysis, with the odds ratio effect size corresponding to an approximate reduction of 31%. Moreover, evaluations conducted in Spain and Norway reduced school‐bullying victimization by approximately 28% and 23%, respectively. Evaluations conducted in Finland, Germany and the UK were also significantly effective, although less so, reducing school‐bullying victimization by approximately 8–12%.

There are many potential explanations for the differences in effectiveness observed between countries. For example, definitions of school‐bullying, and behaviors that constitute bullying, differ between countries. Previous research conducted by Smith et al. (2000) showed that school‐bullying is perceived differently across different countries and cultures and this may explain variability in bullying reporting. Definitions of school bullying, and behaviors that constitute bullying, differ between countries. For example, Smith et al. (2016) showed that school bullying in Eastern cultures manifests more often as exclusion or isolation of an individual victim. In comparison, school bullying in Western cultures comprises a wider range of physical, verbal and relational forms of aggression.

Our meta‐analysis included several examples of cases where the same intervention program was evaluated in different countries (e.g., KiVa program in Finland (Kärnä et al., 2013) and in Italy (Nocentini & Menesini, 2016)). While societal practices, educational systems, and individual lifestyles may differ greatly, some argue that there may be some support for the cross‐national applicability of specific intervention programs. However, there is a current lack of existing research comparing the effectiveness of specific interventions in specific countries.

Previous research has indicated that are also cultural differences in bullying behaviors among adolescents (e.g., Smith et al., 2016). As such, an antibullying program to reduce these behaviors may be impacted by these differences. This is particularly evident when we observe the variations in effect sizes for the OBPP (Olweus, 1993) and the KiVa antibullying program. These programs may be the most well‐known antibullying programs that are commercially available, and as such as the only examples in our review of interventions evaluated in completely different locations.

The OBPP program was originally designed and implemented in Norway, and it is therefore not surprising that the OBPP program appears to be effective in reducing both school‐bullying perpetration and victimization when evaluated in Norway, compared to evaluations in the United States (see Table 13). While the program was still significantly effective in the United States, the percentage decrease in school‐bullying perpetration was roughly 25% and in school‐bullying victimization was roughly 11%. These figures are lesser in comparison to the decreases in bullying behaviors seen in Norwegian evaluations (35% perpetration; 29% victimization). These differences could be attributed to different evaluation methodologies (see Gaffney et al., 2019), however, they most likely reflect cultural and societal differences between youth in Norway and youth in the United States.

Interestingly, the opposite is observed with the KiVa program. When KiVa was evaluated in Finnish samples, the program was effective in reducing school‐bullying perpetration by approximately 4–5% and school‐bullying victimization by approximately 6% (Kärnä et al., 2011a, 2011b, 2013). However, when evaluated in Italian primary and secondary schools, the effect sizes were much larger. Nocentini and Mensini (2016) found that KiVa was effective in reducing school‐bullying perpetration by approximately 15–20% and school‐bullying victimization by approximately 25%.

In the case of KiVa, each of the evaluations used the same methodology (i.e., RCT), but varied greatly in the sample size. Thus, further research is needed to explain why some interventions (e.g., OBPP or KiVa) appear to be more effective in some samples compared to others. The programs are still effective, but the variation in effect size could be attributable to a number of different methodological and implementation factors that warrant further exploration.

#### 10.2.4 Intervention program

Following this logic, we also explored the effectiveness of the specific antibullying programs. Out of the four most widely disseminated antibullying programs included in our review (i.e., KiVA, NoTrap!, OBPP, ViSC), the OBPP was collectively the most effective in reducing school bullying perpetration of these. Across 11 evaluations, the OBPP reduced bullying perpetration by approximately 26%, which was larger than any other widely disseminated program.

In relation to school‐bullying victimization outcomes, the NoTrap! program was the most effective, reducing victimization by around 37%. NoTrap! also reduced bullying perpetration by a considerable amount, approximately 22%, but this effect was not statistically significant. The KiVA program, significantly reduced school bullying perpetration by approximately 9% and school bullying victimization by approximately 11%. The ViSC program was the only program to increase bullying perpetration (by roughly 4%) and bullying victimization (by roughly 4%) although these effects were not statistically significant.

Another moderator we used to code differences between included evaluations was the specificity of the intervention program. In other words, we evaluated each intervention program on how specific it related to bullying behaviors. Unsurprisingly, our findings suggest that antibullying programs gave the largest overall effect sizes. While the significance of the differences between subgroups was not computed due to the large discrepancies between the numbers of evaluations included in each subgroup.

However, our inclusion criteria for the current report was strictly concerned with school‐bullying intervention programs and behavioral outcomes of bullying. As such, we may have overlooked effective programs that only included nonbehavioral outcomes of bullying (e.g., attitudes toward bullying, awareness of bullying) or other problem behaviors (e.g., peer aggression or victimization, mental health issues, juvenile delinquency, etc.) that occur among young people in schools. Changes in these behaviors may also impact bullying, either directly or indirectly, yet, more research is needed to understand this potential effect. Most obvious in the present report is how programs that target specifically school‐bullying may impact cyber‐bullying, and vice versa, given the significant overlap in the prevalence of these behaviors (Baldry et al., 2017).

Further research is also needed to better understand specifically “what works” in these “specific interventions.” In the previous review, (Farrington and Ttofi 2009; Ttofi & Farrington, 2011) conducted detailed coding of interventions and evaluations and analyzed how effect sizes varied between components and features of primary studies. For example, parent training, playground supervision, and more intense and longer programs were significantly correlated with larger reductions in bullying perpetration (Ttofi & Farrington, 2011). Moreover, several intervention components were associated with larger reductions in bullying victimization (e.g., videos, disciplinary methods, co‐operative group work and more intense and longer programs). Therefore, an important avenue for future research is to assess the differences in effectiveness of antibullying programs according to specific intervention components across the 100 evaluations included in our meta‐analysis. Such research would have important implications for policy and the development of future antibullying programs.

Additionally, it appears that since 2009 several large‐scale antibullying programs have been implemented and evaluated (e.g., KiVa; Kärnä et al., 2013; NoTrap!; Menesini et al., 2012; Palladino et al., 2016). Because there is typically more information available on the specific components of these programs, we may be able to code more specific details in future analyses. For example, many studies may fit the criteria for “parent training,” but there is a significant difference between the intensity of parental involvement. For example, some studies may include parents merely by sending letters home with participant children (e.g., Brown et al., 2011), while others include parents more actively by holding information evenings or requiring children to complete take‐home tasks with parental involvement (e.g., Berry & Hunt, 2009; Domino, 2013).

Earlier research highlighted how varying levels of implementation of each intervention component may explain variability in intervention outcomes (Bloom et al., 2003). Interestingly, a narrative review by Smith et al. (2003) reported that although 14 whole‐school antibullying programs obtained modest effects overall, those that monitored implementation obtained twice the mean effects on self‐reported rates of bullying and victimization than those that did not monitor implementation. Thus, additional analyses are required to better understand specifically *what works* in existing antibullying programs and the underlying mechanisms of behavioral change

#### 10.2.5 COI and publication type

Possibly the most conclusive results from our moderator analyses were observed in relation to COI and publication type. First, across both computational models and outcomes, studies that were categorized as being high‐risk for COI were associated with significantly larger reductions in bullying perpetration and victimization. Second, under the MVA model of meta‐analysis, non‐peer‐reviewed evaluations were associated with significantly larger reductions in both bullying perpetration and victimization outcomes. However, the same results were not observed under the random effects.

We examined COI in terms of the involvement of the program developer in the evaluation. Our results may indicate possible sources of biases. For example, it may be that when the individual, or team, that are credited with developing an antibullying program are also involved in the evaluation of said intervention, biases such as confirmation bias may impact the results. However, it may not be a perceivably “negative” source of bias. Perhaps, when the program developer is involved in the implementation of the program, the intervention is simply delivered better and more effectively. There are a number of other factors that could also be affected and in turn impact the effect size, such as teacher and staff efficacy and motivation to participate the in the program.

There are more sophisticated measures of COI (e.g., Eisner et al., 2012) that include elements such as whether or not the evaluator could potentially benefit financially from the intervention program. Further indicators of COI are thus needed to better understand the impact on evaluation results. For example, our findings in relation to COI and larger effect sizes may be explained as: evaluations in which the program developer was included appear to be more effective because of the expertise and intricate knowledge of the developer. Therefore, the results may reflect differences in the quality of program implementation rather than troublesome biases. Additional research is needed.

### 10.3 Limitations and avenues for future research

Like most meta‐analyses, the current report is largely limited by the lack of understanding as to what is the “true effect.” When comparing mean effect sizes between moderators for example, it is difficult to determine the validity of the result. Throughout our discussion of result we discuss that one subgroup of studies was *associated* with larger or smaller effect sizes than another, and the statistical significance of these differences. Thus, we avoid saying studies in subgroup A (e.g., evaluations conducted in Greece) are *more effective* than studies in subgroup B (e.g., evaluations conducted in Italy). Due to the correlational nature of our moderator analyses we cannot make causal inferences. In addition to this limitation, and those previously discussed (Section 9.2), the following section of this report discusses some further limitations.

#### 10.3.1 Measurement of bullying

Experts in the area of school‐bullying research have outlined how there still remain issues of comparability in the assessment of school‐bullying perpetration and victimization (Volk et al., 2017). Studies included in the present meta‐analysis used a wide variety of quantitative measures of school‐bullying behaviors, including self‐report measures (e.g., the Revised Olweus Bully/Victim Questionnaire—Olweus, 1986, 1996), or peer‐report measures (e.g., the Participant Role Questionnaire—Salmivalli et al., 1996). One issue that arises is that the timeframe within which participants are required to indicate the frequency of bullying can vary greatly. One scale may ask about bullying experiences within the last 3 months, while another may ask about ever having experienced, or participated in, school‐bullying. Moreover, included studies utilized a mixture of continuous or dichotomous measures of school‐bullying, and the cut‐off points used to categorize someone as either a bully, victim, or not‐involved also varied.

Furthermore, the majority of evaluations included in our analysis reported bullying outcomes at different time points, largely, before implementation, after implementation, with a possible additional follow‐up time point. However, we computed effect sizes using measures of bullying taken before implementation and immediately post implementation of the intervention. Therefore, we cannot generalize results to the long‐term effectiveness of antibullying programs, or any potential influence of dose‐response effect. Future research should aim to examine the longitudinal effectiveness of interventions to reduce bullying perpetration and victimization in the long‐term.

When conducting our systematic searches for the present review, we did not set restrictions based on measurement issues, other than including quantitative measures of school‐bullying behaviors. However, types of reports, for example, could influence the overall effectiveness effect size. This may possibly explain why our meta‐analysis found that programs are more effective in reducing bullying perpetration outcomes. For example, if programs are concerned with raising awareness about bullying and the associated negative impact on victims, participants who reported bullying perpetration before the intervention may be less likely to self‐report bullying behaviors after completing the program. As a result, the intervention may be perceived as being effective, but the change in reports of bullying may have been a result of social desirability responding (He et al., 2015; Rigby & Johnson, 2006). Conversely, raising awareness on the negative impact of school bullying may lead to increased reporting of victimization due to sensitization effects (Stevens et al., 2000). Notably, sensitization effects due to raised awareness may affect not only self‐report data but also peer nomination data and teacher reports (Smith et al., 2003, p. 597). Therefore, future research could aim to examine whether the style of report used, differing cut‐off points and varying timeframes affect estimations of intervention effectiveness.

#### 10.3.2 Cyberbullying behaviors

Another key limitation of the present review is the omission of cyberbullying behaviors. Prominent researchers in the area have argued that cyberbullying behaviors do not warrant a completely separate line of study, because of the significant overlap between offline and online bullying (Olweus & Limber, 2017). A recent meta‐analysis of cyberbullying intervention and prevention programs found that, out of studies assessing various facets of cyberbullying, a large number were concerned with this overlap (Gaffney et al., 2019). The Gaffney et al. (2019) meta‐analysis concluded that anticyberbullying programs were effective in reducing cyberbullying perpetration by roughly 9–15% and cyberbullying victimization by roughly 14–15%. As illustrated in that other review, there is a need for future research to assess the effectiveness of intervention programs that target both online and offline bullying concurrently. As a result of the significant overlap (e.g., Waasdorp & Bradshaw, 2015), it is important for policy makers, researchers, and program developers to know whether or not these forms of aggressive behaviors should be targeted together or individually. Future research should aim to examine the effectiveness of programs designed to reduce school‐bullying on cyberbullying outcomes, and vice versa. Additional analysis to examine the differences between programs that target offline and online behaviors concurrently in terms of effectiveness to reduce both school‐ and cyber‐bullying is also needed.

#### 10.3.3 Models of meta‐analyses

The current report presents findings using two computational models of meta‐analyses: the random effects model and the multiplicative variance adjustment model. While, the random effects model is often suggested as the preferred model for meta‐analyses in social sciences, for reasons already discussed (Section 7.3), this approach is also limited. However, even though many meta‐analyses in medical sciences (e.g., Ayieko et al., 2014; Dorjee et al., 2018; Woolf‐King et al., 2013) have used the MVA model as an alternative method of accounting for between‐study heterogeneity in weighted mean effect sizes, this model is yet to be widely accepted in behavioral sciences. A number of recent publications (e.g., Portnoy & Farrington, 2015; Zych et al., 2019) have begun to use the MVA model.

It is evident in the current report that the results are influenced by the computational model used. The overall mean effect sizes for bullying perpetration and victimization were not that different under both models but the results of moderator analyses were greatly influenced by how we accounted for the between‐study heterogeneity. Further research is needed in order to examine the reasons for this and also evaluate how best to choose an appropriate computational model when conducting a meta‐analysis.

### 10.4 Concluding remarks

This report presents an updated systematic and meta‐analytical review of the effectiveness of school‐bullying intervention and prevention programs. Overall, our review found that school‐based antibullying programs are effective in reducing both bullying perpetration and bullying victimization, and that effect sizes can vary according to several moderator variables. However, further research is needed to better understand the reasons for variation in observed effect sizes. Research is needed to investigate the specific components of antibullying programs that work best to reduce bullying behaviors. The results of our meta‐analysis have important implications for policy and the development of future antibullying programs, but future research should aim to better understand the effective mechanisms in bullying intervention and prevention.

## 11 TECHNICAL APPENDICES

### 11.1 Calculating the before‐after intervention effect

Williams et al. (2015) evaluated the effectiveness of the Start Strong program based on students' self‐reported experiences of bullying victimization. The primary study found that, at baseline, 23% of participants in the experimental group (*N* = 717) reported bullying victimization, while 23% of participants in the control group (*N* = 800) also reported bullying victimization at baseline. Hence, the baseline OR was calculated as follows (Table 17):

**Table 17:** Data used to estimate baseline odds ratio

	Nonvictims	Victims	*N*
Experimental	552	165	717
Control	616	184	800

Thus, the OR_before_ = 0.999, Ln OR_before_ = −0.002, and *var* Ln OR_before_ = 0.015. Williams et al. (2015) report that after implementation of the Start Strong program, bullying victimization was reported by 28% of experimental participants and 34% of control participants. Accordingly, the posttest OR was calculated as follows (Table 18):

**Table 18:** Data used to estimate postintervention odds ratio

	Nonvictims	Victims	*N*
Experimental	516	201	717
Control	526	272	800

Thus, the OR_after_ = 1.323; Ln OR_after_ = 0.28; and *var* Ln OR_after_ = 0.013. Employing these figures, the ln OR for the intervention effect of the Start Strong program was calculated as:

$$\mathrm{Ln}\;{\text{OR}}_{\text{change}}=\;\mathrm{Ln}\;{\text{OR}}_{\text{after}}-\;\mathrm{Ln}\;{\text{OR}}_{\text{before}},$$

$$\mathrm{Ln}\;{\text{OR}}_{\text{change}}=0.28–(-0.002)=0.282,$$

$$var\;\mathrm{Ln}\;{\text{OR}}_{\text{change}}=0.75x(0.015+0.013)=0.021,$$

$$\text{SE}\;\text{of}\;\mathrm{Ln}\;{\text{OR}}_{\text{change}}=\sqrt{0.021}=0.145.$$

The ln OR_change_ is computed as the difference between the before and after effect size and the variance of this new estimate is adjusted by multiplying the sum of the variances of before and after variances by 0.75. This is an approximation of the assumed correlation between before and after effect sizes. The ln OR_change_ and the SE of ln OR_change_ were then entered into CMA as an estimation of the intervention effect.

### 11.2 Multiplicative variance adjustment

In the present meta‐analysis, the summary effect size estimated for bullying perpetration was OR = 1.324 with 95% confidence intervals of 1.298–1.351 under a fixed effects model. The effect size in the MVA model is the same as the effect size in the fixed effects model. The variance of the effect size in the MVA model is calculated as follows:

$${\mathrm{MVA}}_{\mathrm{var}}={\mathrm{FE}}_{\mathrm{var}}\times \frac{Q}{\mathrm{df}}.$$

Therefore, in the above example of the summary effect size for bullying perpetration outcomes, the FE_var_ is 0.000104. Therefore, with *Q* = 458.555 and *df* = 109, the MVA adjustment for fixed effects is 0.02098, calculated as:

$${\mathrm{MVA}}_{\mathrm{var}}=0.000104\times \frac{458.555}{109}=0.000438.$$

Therefore, the adjusted standard error is 0.0209. In this example thus, the MVA fixed effect is OR = 1.324, and the 95% confidence intervals are 1.271–1.380.

### 11.3 Odds ratio to percentage conversion

The conversion from weighted mean odds ratio to percentage value is also described in the previous Campbell report (see Farrington & Ttofi, 2009). The formula involves assuming equal allocation of participants to experimental and control conditions and that the % of bullies and/or victims was lesser in the experimental condition than in the control condition (as supported by our overall positive mean effect size).

For example, if there are 200 participants in each experimental condition and approximately 30% of participants report bullying victimization in the control condition and 25% victims in the experimental condition, the numbers of victims and nonvictims would be as follows: (Table 19).

**Table 19:** Data used to convert odds ratio to percentage

	Nonvictims	Victims	*N*
Experimental	150	50	200
Control	140	60	200
Total	290	110	400

Therefore using the previously described formula for estimating an odds ratio, the following data would correspond to an odds ratio of 1.286 (i.e., [150 × 60]/[140 × 50]). Moreover, the percentage decrease would be approximately 16.67% (i.e., (10/60) × 100).

Using this basic formula, we can manipulate the % and number of victims in each experimental condition in order to achieve a odds ratio that corresponds to our weighted mean effect size (i.e., MVA: OR = 1.324 and RE: OR = 1.309 for bullying perpetration; MVA: OR = 1.248 and RE: OR = 1.242 for bullying victimization). Using the *n* values that give the closest possible mean effect size we can thus estimate the corresponding percentage reduction in either bullying perpetration or victimization outcomes.

## Footnotes

1 The authors regret that more detailed information concerning specific combinations of keywords and databases searched as per the Campbell MECCIR reporting standards. This information is held on restricted access computers and due to COVID‐19 pandemic, the closure of University buildings, this data could not be retrieved.

2 Web of Science Core Collection database.

3 Unfortunately detailed information about the datas of searches cannot be provided for this review, contrary to MECCIR R35.

4 We were unable to double code in this review. However, as some studies were included in the present review and an earlier review (Farrington & Ttofi, 2009), a proportion of the studies were double‐coded.

5 A worked example is provided in Technical Appendix 10.1.

6 Calculated as: total number of students/number of classrooms.

7 A worked example of this adjustment is provided in Technical Appendix 10.2.

8 Moderator analyses under the MVA model will be greatly affected by the presence of very large studies in the meta‐analysis. Unfortunately, we were not able to follow recommendations made by the methods editor to windsorize weights or conduct sensitivity analyses by removing these large studies. Due to the COVID‐19 pandemic the software to carry out these tests was not available to us. Thus, the reader should consider the impact of large studies when interepting the results of moderator analyses under the MVA model.

9 The procedure used to estimate approximate percentage values for weighted mean odds ratios is provided in Technical Appendix 10.3.

## APPENDIX A

### Appendix: Full Search Syntax

#### Database: Web of Science

Bully* AND Intervention AND Evaluation

Anti‐Bullying AND School AND Program* AND Evaluation

Anti‐Bully* AND Program* AND Outcome

Bully‐victim AND Prevention AND Evaluation

Bully* AND School AND Intervention

Bully* AND School AND Prevention

#### Database: Scopus

Bully* AND School AND Intervention

Bully* AND School AND Prevention

Bully* AND School AND Program*

Bully* AND School AND Evaluation

Bully* AND School AND Intervention AND Evaluation

Bully* AND School AND Prevention AND Evaluation

Anti‐bullying AND Program* AND Evaluation

#### Database: National Criminal Justice Reference Service

Bully* AND Intervention AND Evaluation

Bully* AND Prevention AND Evaluation

Anti‐bullying AND Program* AND Effect*

#### Database: PsycINFO

Bully* AND Intervention AND Program* AND Evaluation

Bully* AND Prevention AND Program* AND Effect*

Anti‐Bully* AND Program* AND Outcome

#### Database: Cochrane Controlled Trials Register

Bully* AND Intervention AND Program*

Bully* AND Intervention AND Program* AND Evaluation

Bully* AND Prevention AND Evaluation

Bully* AND Prevention AND Program AND Evaluation

Anti‐bullying AND Program* AND Effect*

Anti‐bullying AND Program* AND Evaluation

#### Database: British Education Index

Bully* AND Intervention AND Program* AND Evaluation

Bully* AND Prevention AND Program* AND Evaluation

Bully* AND Intervention AND Program* AND Effect*

Bully* AND Prevention AND Program* AND Effect*

Anti‐bullying AND Program* AND Evaluation

Anti‐bullying AND Program* AND Effect*

#### Database: Embase

Bully* AND Intervention AND Evaluation

Bully* AND Prevention AND Evaluation

Anti‐bullying AND Program* AND Evaluation

Anti‐bullying AND Program* AND Effect*

#### Database: MEDLINE

Bully* AND Intervention AND Program* AND Evaluation

Bully* AND Prevention AND Program* AND Evaluation

Anti‐bullying AND Program* AND Effect*

#### Database: ERIC & Criminal Justice Abstracts

Bully* AND Intervention AND Program* AND Evaluation

Bully* AND Prevention AND Program* AND Evaluation

Anti‐bullying AND Program* AND Effect*

www.scholar.google.co.uk

Bully* AND School AND Intervention AND Evaluation

## APPENDIX B

### Appendix: Risk of Bias results for included studies

**Table:**

Study	AS	AC	BE	BC	ID	BOA	CP	SOR	RiskBiasScore
Baldry and Farrington (2004)	U	U	L	L	L	L	H	L	7
Beran and Shapiro (2005)	U	U	U	L	L	L	H	L	9
Berry and Hunt (2009)	L	L	L	L	L	L	U	L	2
Bonell et al. (2015)	L	L	L	H	L	L	L	L	3
Boulton and Flemington (1996)	U	U	H	L	L	U	H	L	12
Brown et al. (2011)	L	U	H	L	L	U	U	L	9
Chaux et al. (2016)	H	H	L	L	L	U	H	L	11
Cissner and Ayoub (2014)	L	H	L	L	L	U	H	L	8
Connolly et al. (2015)	U	L	L	L	L	L	L	L	2
Cross et al. (2011)	U	U	L	H	H	L	L	L	10
DeRosier and Marcus (2005)	U	U	L	L	L	L	H	L	7
Domino (2013)	U	U	L	L	L	U	H	L	9
Espelage et al. (2015)	L	L	L	L	L	U	L	L	2
Fekkes et al. (2006)	U	U	U	U	U	U	L	L	12
Fekkes et al. (2016)	H	H	L	L	L	H	L	L	9
Fonagy et al. (2009)	L	L	L	L	L	U	L	L	2
Frey et al. (2005)	U	U	L	L	L	L	L	L	4
Garaigordobil and Martínez‐Valderrey (2015)	L	H	H	L	U	H	H	L	14
Holen et al. (2013)	L	U	L	L	L	H	L	L	5
Hunt (2007)	U	U	H	L	H	U	L	L	12
Jenson et al. (2013)	U	U	L	L	H	H	L	L	10
Ju et al. (2009)	U	U	L	L	H	U	H	L	9
Kaljee et al. (2017)	U	U	H	H	U	H	L	U	17
Karna et al. (2011b, 2013)	L	L	L	L	L	L	L	L	0
Knowler and Frederickson (2013)	U	U	L	L	L	H	H	L	10
Krueger (2010)	H	H	L	L	L	H	H	L	12
Li et al. (2011)	U	U	L	L	L	U	L	L	6
McLaughlin (2009)	L	U	H	L	L	L	H	L	8
Meyer and Lesch (2000)	U	U	L	L	L	U	H	L	9
Nocentini and Menesini (2016)	L	L	L	L	L	L	L	L	0
Ostrov et al. (2015)	L	L	L	H	L	U	L	L	5
Polanin (2015)	L	L	H	L	H	H	H	L	12
Rosenbluth et al. (2004)	U	U	U	L	H	U	L	L	11
Stallard et al. (2013)	L	L	L	L	L	U	U	L	4
Topper (2011); Preventure	L	H	L	H	H	L	H	U	14
Topper (2011); Adventure	U	U	H	L	H	U	L	U	14
Trip et al. (2015)	U	U	L	L	L	L	L	L	4
Tsiantis et al. (2013)	L	U	H	L	L	L	L	L	5
Waasdorp et al. (2012)	U	L	L	L	L	L	L	H	5
Wolfer and Scheithauer (2014)	U	U	U	L	U	U	H	L	13
Yanagida et al. (2019)	U	H	L	L	L	U	L	L	7
Alsaker and Valkanover (2001)	H	H	H	L	U	H	U	L	16
Andreou et al. (2007)	H	H	L	L	U	H	U	L	13
Battey (2009)	L	U	U	L	L	L	L	L	4
Bull et al. (2009)	U	U	H	L	U	L	H	L	12
Bauer et al. (2007)	H	H	L	H	U	L	H	L	14
Beran et al. (2004)	L	L	L	L	L	H	L	L	3
Elledge et al. (2010)	L	L	L	L	U	L	L	H	5
Evers et al. (2007)	L	L	L	H	H	L	U	L	8
Finn (2009)	U	U	L	U	L	H	L	L	9
Fox and Boulton (2003)	H	H	L	L	L	H	L	L	9
Gollwitzer et al. (2006)	H	H	L	L	L	H	H	L	12
Herrick (2012)	H	H	H	H	L	H	L	L	15
Jornonen et al. (2011)	L	H	L	L	U	H	L	L	8
Kimber et al. (2008)	U	H	L	L	L	H	L	L	8
Losey (2009)	H	H	H	H	L	H	L	L	15
Melton et al. (1998)	L	L	U	L	U	H	L	L	7
Menard and Grotpeter (2014)	H	H	L	L	U	H	L	L	11
Menesini et al. (2003)	H	H	L	L	L	L	H	L	9
Menesini et al. (2012)	H	H	L	L	L	U	H	L	11
Palladino et al. (2012)	H	H	L	L	L	U	H	L	11
Ortega‐Ruiz et al. (2012)	L	U	U	H	U	U	U	L	13
Palldino et al. (2016); Trial 1	H	H	L	L	L	U	H	L	11
Palladino et al. (2016); Trial 2	H	H	L	L	L	U	H	L	11
Pepler et al. (2004)	L	L	U	H	H	U	L	L	10
Pryce and Frederickson (2013)	H	H	U	L	U	U	L	L	12
Rahey and Craig (2002)	U	U	L	L	L	U	L	L	6
Rawana et al. (2011)	U	U	L	L	L	U	L	L	6
Rican et al. (1996)	U	U	U	U	L	U	H	L	13
Sapouna et al. (2010)	L	L	L	L	L	H	U	L	5
Silva et al. (2016)	U	U	L	L	H	H	H	L	13
Solomontous‐Kountouri et al. (2016)	L	L	L	H	H	U	L	L	8
Sutherland (2010)	L	L	U	L	U	U	L	L	6
Toner (2010)	H	H	U	H	L	H	L	L	14
Williams et al. (2015)	L	U	L	L	U	U	L	L	6
Wong et al. (2011)	H	U	L	L	U	U	L	L	9
Yaakub et al. (2010)	U	U	U	U	U	U	L	L	10
Busch et al. (2013)	U	U	U	H	U	U	U	L	15
Ertesvag and Vaaland (2007)	H	H	L	L	L	H	H	L	12
Karna et al. (2011a); Nationwide	L	H	U	L	U	H	L	L	10
Limber et al. (2018)	H	H	L	L	L	U	H	L	11
Olweus/Bergen 1	H	H	U	U	U	U	H	L	17
Olweus/New National	H	H	U	U	U	U	H	L	17
Olweus/Oslo 1	H	H	U	U	U	U	H	L	17
Olweus/Oslo 2	H	H	U	U	U	U	H	L	17
Pagliocca et al. (2007)	H	H	U	U	U	U	H	L	17
Purugulla (2011)	H	H	H	U	U	H	L	L	16
Roland et al. (2010)	H	H	L	L	U	U	L	L	10
Salmivalli et al. (2005)	H	H	L	H	U	U	L	L	13
Whitney et al. (1994)	H	H	U	U	U	U	H	L	17

*Note*: H, hig risk, score 3; L, low risk, score 0; U, unclear risk, score 2. Risk of bias score is estimated as sum of scores on individual risk of bias items.

Abbreviations: AC, Allocation concealment; AS, Allocation sequence; BC, Baseline Equivalence on Characteristics; BE, Baseline Equivalence of Outcome; BOA, Blind Outcome Assessment; CP, Contamination Protection; ID, Incomplete Data; SOR, Selected Outcome Reporting.
